# I can't hear you: effects of noise on auditory processing in mixed-species flocks

**DOI:** 10.1242/jeb.250033

**Published:** 2025-05-22

**Authors:** Trina L. Chou, Megan D. Gall

**Affiliations:** ^1^ Neuroscience and Behavior Program, Vassar College, Poughkeepsie, NY 12604, USA; ^2^ Biology Department, Vassar College, Poughkeepsie, NY 12604, USA

**Keywords:** Hearing, Critical ratios, Songbirds, Physiology

## Abstract

Animals have evolved complex auditory systems to extract acoustic information from natural environmental noise, yet they are challenged by rising levels of novel anthropogenic noise. Songbirds adjust their vocal production in response to increasing noise, but auditory processing of signals in noise remains understudied. Auditory processing characteristics, including auditory filter bandwidth, filter efficiency and critical ratios (level-independent signal-to-noise ratios at threshold), likely influence auditory and behavioral responses to noise. Here, we investigated the effects of noise on auditory processing in three songbird species (black-capped chickadees, tufted titmice and white-breasted nuthatches) that live in mixed-species flocks and rely on heterospecific communication to coordinate mobbing behaviors. We determined masked thresholds and critical ratios from 1 to 4 kHz using auditory evoked potentials. We predicted that nuthatches would have the lowest critical ratios given that they have narrowest filters, followed by titmice and then chickadees. We found that nuthatches had the greatest sensitivity in quiet conditions, but the highest critical ratios, suggesting their auditory sensitivity is highly susceptible to noise. Titmice had the lowest critical ratios, suggesting relatively minor impacts of noise on their auditory processing. This is not consistent with predictions based on auditory filter bandwidth, but is consistent with both recent behavioral findings and predictions made by auditory filter efficiency measures. Detrimental effects of noise were most prevalent in the 2–4 kHz range, frequencies produced in vocalizations. Our results using the critical ratio as a measure of processing in noise suggest that low levels of anthropogenic noise may influence these three species differently.

## Teaser

**Summary:** Songbird species differ in their ability to process signals in noise, mirroring patterns of auditory filter efficiency. Increasing anthropogenic noise may differentially impact species' acoustically mediated behaviors.

## INTRODUCTION

For many animals, detecting and discriminating among sounds is vital to their survival. Sounds can be divided into those of interest (i.e. provide information such as cues and communication signals) and those competing with signals of interest (i.e. noise; Wiley, 2015). Environmental noise can arise from abiotic (wind, moving water) and biotic (insects, birds) sources (Luther and Gentry, 2013). Importantly, noise can be filtered out by a receiver's auditory system (Lawley and Prather, 2022), with selection favoring auditory processing systems that can detect stimuli and extract information in acoustically complex environments. By gaining information from signals, receivers can mediate their behavior to respond to their environment in ways that promote survival. However, animals are increasingly confronted with anthropogenic noise that differs from natural environmental noise in its amplitude, spectral and temporal properties (Slabbekoorn et al., 2018). The effects of anthropogenic noise on communication have been well documented, particularly in songbirds, but the focus has largely been on changes in signal production and behavioral responses to signals (Slabbekoorn and Peet, 2003; Francis et al., 2011; Dowling et al., 2012; Arroyo-Solís et al., 2013; LaZerte et al., 2016, 2017). Our knowledge of how anthropogenic noise influences auditory processing is relatively limited in comparison (but see Pohl et al., 2009, 2012; Fossesca et al., 2023; Francis et al., 2023). A complete understanding of how anthropogenic noise affects acoustically mediated behaviors, such as communication or predator avoidance, requires us to understand not only how species differ in the ways that anthropogenic noise affects signal production, but also the ways in which species differ in their ability to extract relevant acoustic information from background noise (Erbe et al., 2016; Fossesca et al., 2023; Francis et al., 2023).

**Fig. 1.**
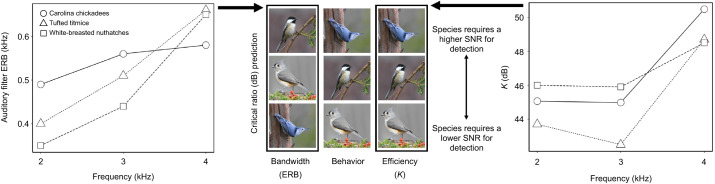
**Predictions for critical ratio patterns based on published auditory filter equivalent rectangular bandwidths (ERB), filter efficiencies (*K*) and behavioral results for chickadees, tufted titmice and white-breasted nuthatches.** Auditory filter ERBs and filter efficiency data obtained from Henry and Lucas (2010a), Henry and Lucas (2010b) and Henry et al. (2011). Behavioral data obtained from Chou et al. (2023). Photo credit: Jack Bulmer.

We chose to investigate auditory processing in noise in three songbird species: black-capped chickadees (*Poecile atricapillus*, hereafter chickadees), tufted titmice (*Baeolophus bicolor*, hereafter titmice) and white-breasted nuthatches (*Sitta carolinensis*, hereafter nuthatches). Not only have these three species long been models for acoustic communication (Otter, 2007), but there has also been significant recent interest in the effects of anthropogenic noise on their communication and antipredator behaviors (Proppe et al., 2012; LaZerte et al., 2016; Damsky and Gall, 2017; Courter et al., 2020; Jung et al., 2020), allowing us to link their auditory processing to their ecologically relevant behaviors in noise. Chickadees, titmice and nuthatches make up mixed-species flocks and jointly engage in predator mobbing, with effective mobbing behavior being dependent on the level of species' involvement in the group (Hurd, 1996; Nolen and Lucas, 2009). Recent work suggests that high-amplitude noise affects all three species in a similar way: behavioral responses to acoustic mobbing signals decreased in all three species when exposed to high-amplitude anthropogenic noise (Damsky and Gall, 2017). However, lower amplitude noise differentially impacts responses to acoustic mobbing signals in these three species: nuthatch behavioral responses to signals were significantly reduced in noise, whereas titmice showed little change in behavior under similar conditions, and chickadees showed an intermediate level of response (Chou et al., 2023). We hypothesized that these species-level differences in behavior could be partially explained by differences in auditory processing in noise. If this hypothesis is correct, we would expect nuthatches to have the poorest auditory processing in noise, followed by chickadees and then titmice (Fig. 1).

Critical ratios are likely to be a useful tool for comparing auditory processing in noise across species. A critical ratio is the signal-to-noise ratio (SNR in dB) at which a tone can just be detected in white noise (i.e. masked thresholds), independent of absolute noise levels (Long, 1994). Species with higher critical ratios are likely to have poorer processing in noise, as higher critical ratios indicate that the signal needs to exceed the noise by a greater amount in order to be detectable. Large-scale comparative approaches may benefit from the use of critical ratios that can be easily collected and compared across species (Francis et al., 2023), and these results can be particularly relevant for species that extract simple signals from noise (e.g. species that sing relatively tonal songs; Weisman et al., 1990; Nowicki et al., 1992; Rivera-Gutierrez et al., 2010) or species that participate in group behaviors that require detection of conspecific and heterospecific signals to coordinate behavior (Dutour et al., 2017). A comprehensive understanding of species differences in auditory processing will help us predict how communication systems and other acoustically mediated behaviors will respond to increasing anthropogenic noise (Fossesca et al., 2023; Francis et al., 2023). Although critical ratios do not capture the full complexity of auditory processing in noise (see Klump and Langemann, 1995; Hofer and Klump, 2003), they remain a useful and practical first approximation of auditory processing in noise, particularly for comparative work.

Critical ratios are most useful when we have a strong understanding of their relationship with other measurements of peripheral auditory processing, allowing us to compare new estimates of auditory processing with those that have already been collected. In the bandpass filter model of the auditory periphery, the detection of a pure tone (or other narrowband stimulus) by a single auditory filter is diminished by masking noise processed in the same filter (Fletcher, 1940; Glasberg and Moore, 1990). Critically, the width of the filter at any given frequency is thought to determine the overall level of masking produced by a broadband masker. Wider auditory filters process more noise concomitantly with the signal of interest relative to narrower auditory filters, effectively increasing the masking power of the noise relative to the signal (Moore, 2012). Based on this observation, we would predict that species with broader auditory filters would have poorer auditory processing in noise, and thus higher critical ratios.

Early estimates of auditory filter bandwidth using broadband maskers assumed that masking occurs when the energy in the noise and the energy of the tone are equal (i.e. equal power; Fletcher, 1940; reviewed in Erbe et al., 2016). Thus, filter bandwidths could be calculated directly from the masked threshold of a tone in noise and the spectrum level of that noise (Fletcher, 1940). However, the equal power assumption is violated in some species, leading to a mismatch between critical ratios and measurements of tuning properties directly from auditory neurons. More modern estimates of filter bandwidth, using notch-noise methods and a rounded exponential model (roex), include a parameter (*K*) that describes the efficiency of the filter (i.e. the signal level required to alter the output of a filter in a given level of noise in dB; Patterson, 1976; Patterson and Nimmo-Smith, 1980; Glasberg and Moore, 1990; Glasberg and Moore, 2000; Baker and Rosen, 2006). Based on these observations, we would predict that species with higher estimates of the filter efficiency parameter (*K*) would have poorer auditory processing in noise irrespective of filter bandwidth, and thus higher critical ratios.

Indeed, it turns out that both auditory filter bandwidth and filter efficiency estimates vary significantly across both frequencies and songbird species (Dooling and Saunders, 1975; Okanoya and Dooling, 1987; Langemann et al., 1995; Marean et al., 1998; Henry and Lucas, 2010a,b). Filter bandwidth and efficiency have been determined for titmice (Henry and Lucas, 2010a), nuthatches (Henry and Lucas, 2010a) and Carolina chickadees (*Poecile carolinensis*; Henry and Lucas, 2010b), but not for black-capped chickadees. Nuthatches have slightly narrower auditory filters than titmice, and chickadees have the broadest filters (Fig. 1). This is particularly true at 2 and 3 kHz, whereas at higher frequencies (e.g. 4 kHz) the species have more similar filter bandwidths (Fig. 1; Henry et al., 2011). Moreover, the bandwidth by frequency function differs across the species, with bandwidth increasing rather linearly in titmice and nuthatches, and chickadees having a much flatter function. If critical ratios are predicted by bandwidths in these three species, then we expected chickadees to have the highest critical ratios, followed by titmice and then nuthatches (Fig. 1, left). For filter efficiency, titmice have the lowest filter efficiency parameter estimates (i.e. *K*, the lowest SNR at threshold), whereas white-breasted nuthatches have the highest filter efficiency parameter estimates (Henry and Lucas, 2010a,b). For all three species, filter efficiency parameter estimates were higher (i.e. less good at extracting signals from noise) at 4 kHz than at 2 or 3 kHz. If efficiency is a better predictor of critical ratios, then we expected nuthatches to have the highest critical ratios, followed by chickadees and then titmice (Fig. 1, right).

At present, it remains unclear whether auditory processing metrics, such as critical ratios, can predict the effects of noise on ecologically relevant behaviors in free-living birds. Moreover, it remains unclear whether filter bandwidth or efficiency will be most closely correlated with estimates of critical ratios. Therefore, using three species of wild-caught songbirds (chickadees, titmice and nuthatches), we tested our hypotheses that critical ratios: (1) can predict patterns of ecologically relevant behaviors in noise, (2) are predicted by auditory filter bandwidth and (3) are predicted by auditory filter efficiency. In each of these species, we used auditory evoked potentials to determine thresholds for pure tones in quiet and in three levels of background noise (bandwidth-limited white noise), from which we then calculated critical ratios. We then compared these critical ratios with previously determined behavioral responses to signals in noise, as well as previously published estimates of filter bandwidths and filter efficiency. We predicted that: (1) if auditory processing in noise influences ecologically relevant behaviors, then nuthatches would have the highest critical ratios, chickadees would have intermediate critical ratios and titmice would have the lowest critical ratios; (2) if auditory filter bandwidth predicts critical ratios, then chickadees would have the highest critical ratios, titmice would have intermediate critical ratios and nuthatches would have the lowest critical ratios; and (3) if auditory filter efficiency predicts critical ratios, then nuthatches would have the highest critical ratios, chickadees would have intermediate critical ratios and titmice would have the lowest critical ratios (Fig. 1).

## MATERIALS AND METHODS

### Capture and housing

Seven black-capped chickadees [*Poecile atricapillus* (Linnaeus 1766)], six tufted titmice [*Baeolophus bicolor* (Linnaeus 1766)] and four white-breasted nuthatches (*Sitta carolinensis* Latham 1790) were captured using baited walk-in traps at the Vassar College Ecological Farm and Preserve in Poughkeepsie, NY, USA. Captures occurred between 7 February and 30 March 2023. Individuals were captured in the morning and transported to Vassar College in Poughkeepsie, NY, USA. Birds were singly housed and provided with perches and *ad libitum* access to black-oil sunflower seed and water. Each individual was weighed and banded with a USGS band. Chickadees and titmice were also banded with unique color band combinations. Average body masses before auditory evoked potential (AEP) collection were 10.73±0.87 g for chickadees, 22.27±1.56 g for titmice and 20.83±0.43 g for nuthatches. AEP experiments were run on the same day as capture between 10:00 and 14:00 h. After completion of the AEP experiments, birds were allowed to recover from anesthesia for at least 4 h. After the individuals were observed eating and eliminating waste, they were released at the capture site. All birds were in captivity for less than 12 h, and all procedures were approved under Vassar College IACUC protocol no. 20-06B and the appropriate state and federal permits.

### Auditory evoked potentials

All tests were performed in a 1.8×1.9×2 m IAC Acoustics (Naperville, IL, USA) audiology booth lined with pyramidal acoustic foam to provide sound deadening. At the start of the experiment, each bird was anesthetized with an intramuscular injection of ketamine (50±2.56 mg kg^−1^) and xylazine (10±0.51 mg kg^−1^), or a combination of ketamine (50±8.93 mg kg^−1^), xylazine (2±0.36 mg kg^−1^) and midazolam (5±0.89 mg kg^−1^). The bird was placed on a microwaveable heating pad topped with several layers of surgical towels to maintain body temperature. The temperature between the bird and the outermost towel was maintained at 39±1°C by adding or removing towel layers.

After the bird was fully sedated, its skin was cleaned with 70% isopropyl alcohol and three 27-gauge 12 mm subdermal needles (Rochester Electro-Medical Inc.; Lutz, FL, USA) were inserted: one inverting electrode directly below the auditory meatus of the right ear, one non-inverting electrode at the vertex of the head, and one grounding electrode at the nape of the neck. The electrodes were connected to a Tucker Davis Technologies (TDT; Alachua, FL, USA) RA4LI head stage with a RA4PA preamp, which then fed into a TDT RZ6 processor via a fiber optic cable. After the electrodes were inserted, the bird was moved into a small Faraday cage (157.5×73.7×116.8 cm) with the bird's right ear facing up.

We assessed auditory thresholds in quiet and in three noise conditions: 44, 54 and 64 dB_A_, corresponding to spectrum levels of 6, 16 and 26 dB Hz^−1^, respectively. The order of the noise presentations and frequencies was randomized across subjects. To determine auditory thresholds, each subject was presented with a set of 5-ms tones (1-ms Blackman–Harris gating) at five frequencies (1, 2, 2.5, 3.15 and 4 kHz) at a rate of 51.1 stimuli s^−1^. At each frequency, we played tones at 60 dB SPL to allow for suprathreshold comparisons across frequencies. Below 60 dB, different stimulus amplitude intervals were used depending on known thresholds for each frequency to assess thresholds more rapidly. Larger steps (10 or 20 dB) were used farther away from the threshold and smaller steps (5 dB) were used near the threshold. The exact set of stimuli amplitudes varied by frequency. Two sets of 400 stimuli were played in alternating phases for each combination of stimulus frequency and amplitude. Between each set of frequencies, we also assessed the bird's response to a click in both quiet and noise to (1) monitor the physiological stability of the subject and (2) ensure there was no adaptation to our noise presentations.

Tone stimuli were generated in SigGenRZ (v 5.6.0). White noise was generated in PRAAT (v 6.2.18), bandpass filtered between 0 and 6.3 kHz, and saved as an hour-long WAV file. The white noise was played back from Audacity (v 3.2.4) and flattened with a 31-band EQ351 ART equalizer. The tones and noise were then combined in a Radial Engineering two-channel passive mixer, amplified with a Crown XLi800 amplifier, and played through an Orb Mod2 satellite speaker located 0.5 m above the bird's head (Orb Audio; Sherman Oaks, CA, USA; frequency response: 0.12–15 kHz). Stimulus presentation and auditory evoked recordings were coordinated by BioSigRZ (v. 5.6.0), a POE_5_ signal processing card and an RZ6 processor. Prior to the experiment, the level of each tone coming out of the speaker was measured in 1/3 octave bands with a Larson Davis LXT sound level meter, and each frequency's gain was then adjusted using the gain function in SigGenRZ to equalize across the spectrum. The level of the white noise was also measured in 1/3 octave bands with the SLM and adjusted through the equalizer to ensure equal amplitudes across all frequencies (±<1 dB). Auditory evoked responses were band-pass filtered between 0.03 and 10 kHz and notch-filtered at 60 Hz.

### Auditory brainstem response analyses

**Fig. 2.**
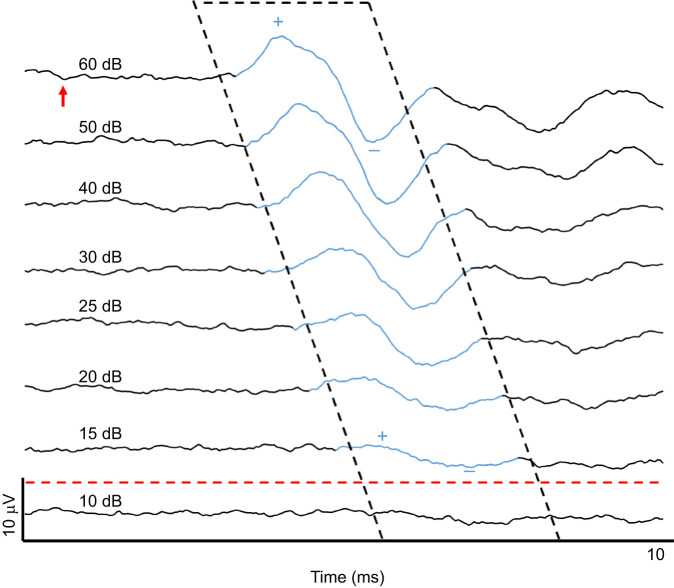
**Auditory brainstem response (ABR) voltage traces decrease in amplitude and increase in latency as stimulus levels decrease.** ABR traces from a single white-breasted nuthatch in response to a 2.5 kHz tone played at eight stimulus amplitudes, with the response colored blue. Thresholds were estimated as the sound pressure level halfway between that of the last detectable response and the next lowest stimulus, and in this example would be estimated as 12.5 dB. Traces depict the average of two measurements, and ABR responses were required in both measurements in order to be counted as present. The red arrow in the top trace indicates the time at which the sound reaches the bird's ear. Plus and minus signs indicate the first positive (+) and negative (−) peak for the trace to the 60 dB tone and the trace to the 15 dB tone, the last trace in which the ABR response was detectable.

**Fig. 3.**
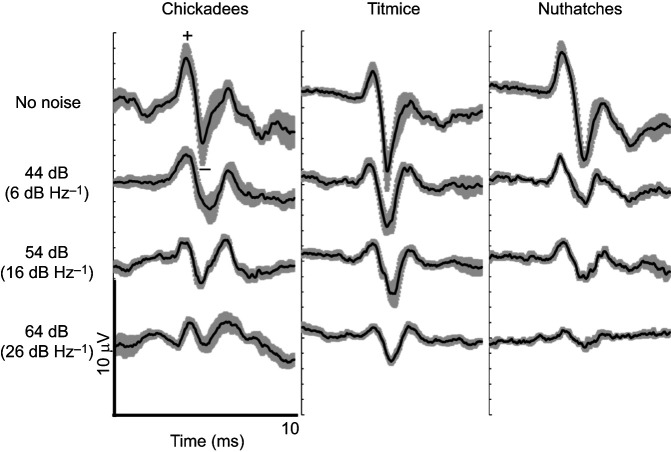
**In response to a loud tone (60 dB SPL), species experience decreased ABRs as noise increases, with nuthatches demonstrating the most suppressed responses.** Mean (±s.e.m) ABR voltage traces for black-capped chickadees (*N*=6), tufted titmice (*N*=7) and white-breasted nuthatches (*N*=4). ABRs were evoked by a 2.5 kHz tone played at 60 dB SPL in each of the four noise conditions. We measured ABR amplitude as the voltage difference between the first positive (+) and negative (−) peak, as well as the latencies to both peaks.

We analyzed the response of the animals to the onset of sound using a type of AEP known as the auditory brainstem response (ABR). In birds, the first positive and negative peaks are thought to represent the response of neurons in the auditory nerve to the onset of sound (Corwin et al., 1982; Møller and Jannetta, 1985). Auditory thresholds at each frequency were determined using visual detection, where two trained observers independently identified the lowest stimulus amplitude evoking a response. Each measurement was the average of two replicates in response to the same intensity, and an ABR response was classified as present if there was a clear positive and negative peak across both measurements – if one of the measurements had an ABR response but not the other, then no response was recorded. Thresholds were estimated as the sound pressure level halfway between that of the last detectable response and the next quietest stimulus (Table S1). Because stimulus intensities in threshold regions differed by 5 dB, ABR thresholds were defined as the intensity 2.5 dB below the lowest stimuli amplitude at which a response could be visually detected (Fig. 2). Critical ratios for the quiet and masking conditions were found by subtracting the spectrum level of the masking noise from the corresponding sound pressure level at the masked threshold for each stimulus frequency. We also analyzed ABR amplitudes and latencies for AEP suprathreshold responses to 60 dB SPL stimuli (Fig. 3; Table S2). ABR amplitude was defined as the voltage difference between the first positive peak and the first negative peak. We measured latency from the onset of sound at the ear to the first positive peak and the first negative peak of the ABR.

Our analysis of the responses to click stimuli suggested that titmice and nuthatch responses were quite stable over time (low coefficients of variation), with slight variability in chickadees, although no individuals were determined to be outliers. There were no signs of adaptation to noise in any of the three species. Thus, all individuals were included in our statistical models.

### Statistical analyses

Exploratory data and distribution analyses were conducted in R (v 4.2.0) using the fitdistrplus and MASS packages. Repeated-measures general linear mixed models (proc mixed) for all variables were created in SAS (v 9.3), resulting in five statistical models: one for thresholds, one for critical ratios, one for ABR amplitude, and one each for latency to the first positive and negative peak. Both critical ratio and ABR amplitude data were log-transformed to achieve normality. All models included the within-subject class variables of frequency (1, 2, 2.5, 3.15 and 4 kHz) and spectrum level (no noise, 6, 16 and 26 Hz^−1^), as well as the between-subject class variable of species (chickadee, titmouse or nuthatch) and all of their interactions. Subject ID was included as a subject factor in the repeated statement for all models. We used an autoregressive covariance structure, and degrees of freedom were calculated with the Kenward–Rogers algorithm as these resulted in the best model fit. We compared the AIC values from full models with those with non-significant interactions removed to determine the final model. Results of the full and reduced models were qualitatively quite similar. Significant effects were explored *post hoc* (Bonferroni corrected) using the diffs procedure in SAS, which explores the differences in least-squared means. Code can be found in the supplementary information (Script 1). Graphs were produced in R using the ggplot2 package.

## RESULTS

### Thresholds

**Fig. 4.**
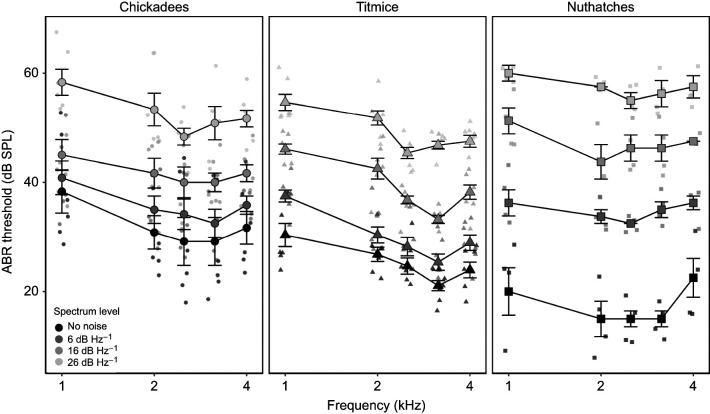
**Masked ABR thresholds increased with increasing levels of noise, particularly for nuthatches.** Masked ABR thresholds for black-capped chickadees (*N*=6), tufted titmice (*N*=7) and white-breasted nuthatches (*N*=4) are shown as a function of stimulus frequency and masking noise. Thresholds were determined using the visual detection method. Data are shown as means±s.e.m, with jittered points representing individual data points.

Thresholds were generally best at previously known frequencies of best sensitivity, being lowest at intermediate frequencies with increasing thresholds at higher and lower frequencies (Fig. 4). Overall, thresholds increased as noise levels increased (*F*_3,152_=167.63, *P*<0.001), but importantly there were differences in those increases across both species (*F*_2,38.7_=7.40, *P*=0.002) and frequencies (*F*_4,262_=49.91, *P*<0.001). This was seen in significant interaction effects of species×frequency (*F*_8,264_=5.56, *P*<0.001) and species×noise (*F*_6,158_=8.25, *P*<0.001). In the no noise condition, all three species' thresholds were significantly different from each other, with nuthatches having the lowest and chickadees having the highest thresholds (*t*_65.9_≥3.75, *P*≤0.001). However, this pattern changed as noise levels increased. In the 6 dB Hz^−1^ noise condition, chickadee thresholds were only significantly higher than titmice thresholds, which were the lowest (*t*_89_=3.22, *P*=0.002), whereas at 16 and 26 dB Hz^−1^, nuthatches had significantly higher thresholds than both chickadees (*t*_65.9_≥2.21, *P*≤0.031) and titmice (*t*_89_≥3.37, *P*≤0.001; Fig. 4). Titmice demonstrated significantly lower thresholds at higher frequencies than both chickadees (*t*_88.3_≥3.92, *P*<0.001) and nuthatches (*t*_88.3_≥3.69, *P*<0.001), with no differences between chickadees and nuthatches (*t*_88.3_≤0.21, *P*≥0.837) when averaged across noise levels. No other pairwise comparisons were statistically significant. Overall, titmice had the lowest thresholds averaged across all conditions, with chickadees having significantly higher thresholds (*t*_38.7_=3.78, *P*<0.001) and nuthatches having marginally higher thresholds (*t*_38.7_=2.21, *P*=0.033). The noise×frequency and noise×species×frequency interaction effects were not included in the final model.

### Critical ratios

**Fig. 5.**
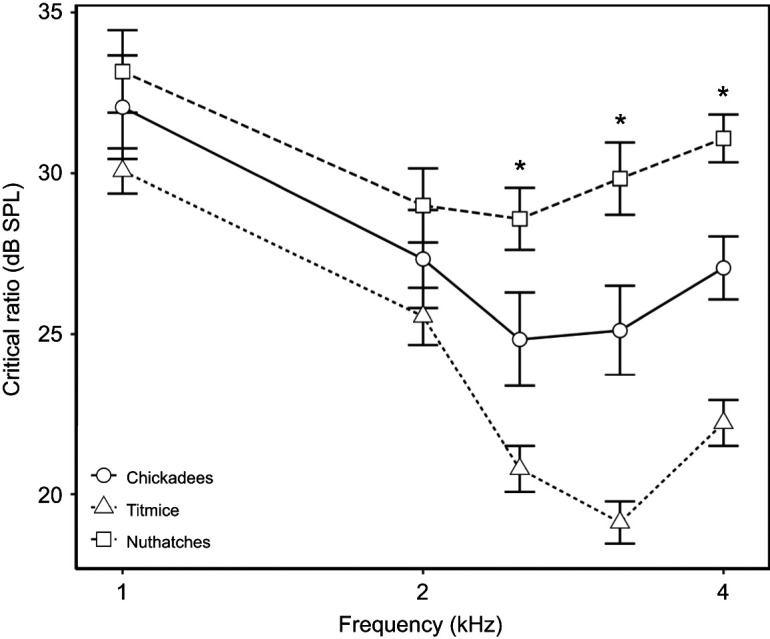
**Critical ratios differ across species, particularly in the 2–4 kHz range.** Critical ratios for black-capped chickadees (*N*=6), tufted titmice (*N*=7) and white-breasted nuthatches (*N*=4) are shown as a function of stimulus frequency. Critical ratios were independent of noise level; thus, each point represents the critical ratios averaged across noise levels. Data are shown as means±s.e.m. Frequencies at which all pairwise *post hoc* comparisons of the three species indicate significant differences in critical ratios are also shown (*P<0.017).

Critical ratios were generally best at the frequencies of best sensitivity, creating a U-shaped pattern as frequency increased (*F*_4,192_=39.18, *P*<0.001), but differences across species emerged at higher frequencies (*F*_2,40.3_=17.56, *P*<0.001). The main effect of noise was not significant (*F*_2,111_=0.91, *P*=0.41), indicating that critical ratios were independent of noise level. Overall, all three species had significantly different critical ratios, with nuthatches having the highest and titmice having the lowest critical ratios. Critical ratios were lowest at intermediate frequencies and increased at higher and lower frequencies across species (*t*_40.3_≥2.58, *P*≤0.014; Fig. 5). We also found a significant interaction effect of species×frequency (*F*_8,184_=5.06, *P*<0.001), with differences across species found only at higher frequencies. There were no differences across species at frequencies at or below 2 kHz (*t*_119_≤1.99, *P*≥0.049). However, nuthatch critical ratios were higher than those of chickadees (*t*_128_≥2.46, *P*≤0.015) and titmice (*t*_128_≥5, *P*<0.001) at frequencies at or above 2.5 kHz. Additionally, chickadee critical ratios were higher than titmice critical ratios (*t*_119_≥2.7, *P*≤0.008) at these frequencies. The species×noise, noise×frequency and noise×species×frequency interaction effects were not included in the final model.

### Amplitudes

**Fig. 6.**
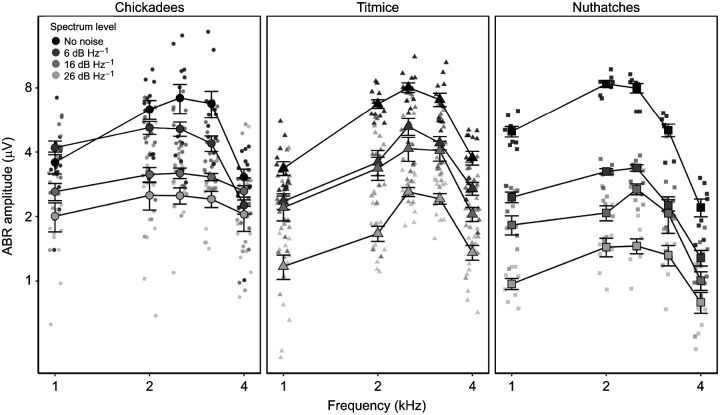
**ABR amplitudes demonstrate similar patterns to masked thresholds and critical ratios, with nuthatches demonstrating disproportionate decreases in amplitude as noise levels increase.** Amplitudes for black-capped chickadees (*N*=6), tufted titmice (*N*=7) and white-breasted nuthatches (*N*=4) are shown as a function of stimulus frequency and masking noise. Data are shown as means±s.e.m, with jittered points representing individual data points.

Amplitudes were greatest in response to intermediate frequencies of best sensitivity (*F*_4, 574_=133.48, *P*<0.001) and decreased as noise increased (*F*_3,136_=121.39, *P*<0.001), but the amount of decrease was different across species (*F*_2,83.3_=21.33, *P*<0.001) and frequencies (*F*_4,574_=133.48, *P*<0.001) (Fig. 6). Overall, nuthatches had smaller ABR amplitudes than both chickadees (t_82.9_=6.21 *P*<0.001) and titmice (*t*_83.4_=5.38, *P*<0.001), and there were no differences between chickadee and titmice amplitudes. The species×frequency (*F*_8,585_=9.66, *P*<0.001), species×noise (*F*_6,179_=3.92, *P*=0.001) and noise×frequency (*F*_12,559_=3.52, *P*<0.001) interactions were significant. Nuthatch ABR amplitudes were significantly smaller than chickadee ABR amplitudes (*t*_259_≥3.08, *P*≤0.002) across all frequencies, but were only smaller than titmice ABR amplitudes (*t*_270_≥6.41, *P*<0.001) at frequencies at or above 2.5 kHz. In the no noise condition, there was no difference across the three species in ABR amplitude (averaged across frequency; *t*_120_≤1.07, *P*≥0.29). However, in all three noise conditions, nuthatches had significantly smaller amplitudes than both chickadees (*t*_164_≥5.45, *P*<0.001) and titmice (*t*_164_≥5.45, *P*<0.001; Fig. 6), with no difference between chickadees and titmice (*t*_120_≤2.43, *P*≥0.017). The noise×frequency effect was significant but small, and pairwise comparisons resulted in minute differences in amplitude responses to frequencies across noise levels. The three-way interaction was not included in the final model.

### Latencies

Latencies to both positive and negative peaks decreased as frequency increased in the no noise and 6 dB Hz^−1^ conditions. Although this trend continued for nuthatches and titmice in the 16 and 26 dB Hz^−1^ conditions, chickadee latencies increase only in response to 4 kHz as noise levels increased.

**Fig. 7.**
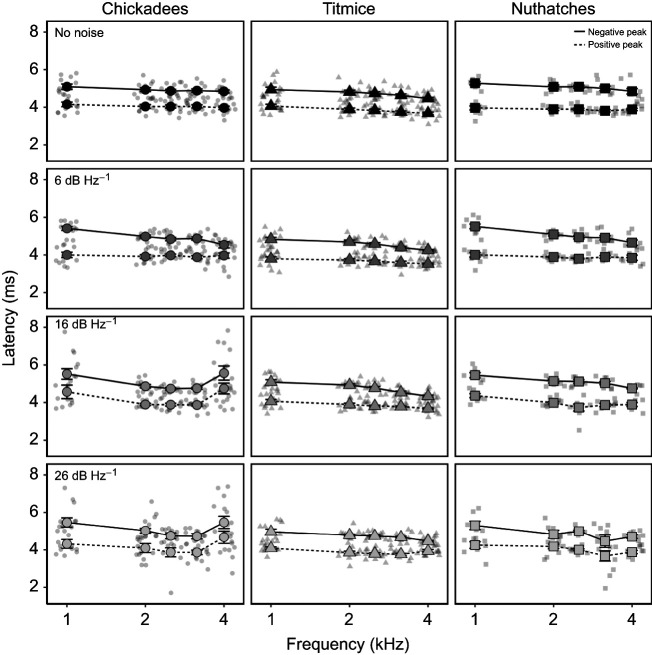
**Titmice had the shortest ABR latencies for both their positive and negative ABR peaks across noise conditions.** Latencies for black-capped chickadees (*N*=6), tufted titmice (*N*=7) and white-breasted nuthatches (*N*=4) are shown as a function of stimulus frequency and masking noise, with different line types indicating latencies to the positive and negative peaks and jittered points representing individual data points. Data are shown as means±s.e.m., with jittered points representing individual data points.

For latency to the positive peak, there were significant main effects of species (*F*_2,129_=13.43, *P*<0.001), noise (*F*_3,172_=3.67, *P*=0.014) and frequency (*F*_4,550_=9.63, *P*<0.001). Overall, chickadees had significantly greater latencies than both titmice (*t*_129_=5.18, *P*<0.001) and nuthatches (*t*_128_=2.49, *P*=0.014), whereas titmice had marginally shorter latencies than nuthatches (*t*_129_=2.12, *P*=0.036; Fig. 7). Latencies in the no noise and 6 dB Hz^−1^ noise conditions were shorter than in the 16 dB Hz^−1^ (*t*_191_≥2.68, *P*≤0.008) and 26 dB Hz^−1^ (*t*_159_≥3.04, *P*≤0.003) noise conditions. These patterns were complicated by significant interaction effects of species×frequency (*F*_8,564_=3.31, *P*=0.001) and noise×frequency (*F*_12, 531_=2.32, *P*=0.007). In the no noise and 6 dB Hz^−1^ noise conditions, there were no differences in latencies across frequencies (t_474_≤2.18, *P*≥0.030) when the three species were considered together. However, at 16 and 26 dB Hz^−1^, latencies were shortest at the frequencies of best sensitivity (2–3.15 kHz), with longer latencies at 1 kHz (*t*_605_≥3.61, *P*<0.001) and 4 kHz (*t*_605_≥3.14, *P*≤0.002). These interaction effects were driven by differences primarily in chickadees at 4 kHz, with chickadees having longer latencies than both titmice (*t*_368_=6.85, *P*<0.001) and nuthatches (*t*_386_=4.44, *P*<0.001) in the loudest noise conditions. Although the noise×species and noise×species×frequency interaction effects were not included in the final model, the increased latencies at 4 kHz in higher noise conditions seemed to be driven more by the patterns of the chickadees than those of titmice or nuthatches (Fig. 7).

For latency to the negative peak, there were significant main effects of species (*F*_2,98.9_=14.31, *P*<0.001) and frequency (*F*_4,578_=23.01, *P*<0.001). The main effect of noise was not significant (*F*_3,150_=1.41, *P*=0.243). Overall, titmice had significantly shorter latencies than both chickadees (*t*_99.1_=4.72, *P*<0.001) and nuthatches (*t*_99_=4.33, *P*<0.001; Fig. 7). For all three species, latencies were greatest in response to 1 kHz (*t*_605_≥5.34, *P*<0.001) and decreased as frequency increased (Fig. 7). There were also significant interaction effects of species×frequency (*F*_8,591_=5.95, *P*<0.001) and noise×frequency (*F*_12,563_=2.05, *P*=0.018). Chickadees had significantly longer latencies in response to 4 kHz stimuli than both titmice (*t*_307_=6.98, *P*<0.001) and nuthatches (*t*_328_=3.65, *P*<0.001). Despite the main noise effect not being significant, the significant noise×frequency effect was mainly seen in response differences across frequencies in the noise conditions. The noise×species (*F*_6,132_=0.95, *P*=0.463) and noise×species×frequency (*F*_24,537_=1.44, *P*=0.083) interaction effects were not significant.

## DISCUSSION

In three species of wild-caught songbirds (chickadees, titmice and nuthatches), we tested the hypotheses that critical ratios: (1) can predict patterns of ecologically relevant behaviors in noise, (2) are predicted by auditory filter bandwidth and (3) are predicted by auditory filter efficiency. Using AEPs to determine masked thresholds and critical ratios, we found that critical ratio patterns and behavioral patterns agree, and that for these species filter efficiency was a better predictor of critical ratios than filter bandwidths. Nuthatches had the highest critical ratios, followed by chickadees and then titmice, who had the best auditory processing abilities in noise. This finding was consistent with previously studied ecologically relevant behavioral patterns, where nuthatches were more susceptible to masking in low levels of noise than both chickadees and titmice (Chou et al., 2023). Differences in critical ratios across species were most prominent at frequencies of best sensitivity, which are used extensively for communication. Deficits in this range of hearing thus have important implications for resulting behavior and communication with other individuals. Species patterns were consistent across masked thresholds, critical ratios, ABR amplitudes and ABR latencies.

### Critical ratios and communication

Understanding species-level differences in sensory processing may help us understand how animals respond to environmental change, particularly sensory pollutants. Visual ecologists have long incorporated models of visual processing when considering visual signaling (Endler et al., 2005; Osorio and Vorobyev, 2008; Kelley and Kelley, 2014). Visual processing, for instance the light gathering capabilities of the eye, have even been shown to predict the effects of anthropogenic light on reproductive success in a variety of songbirds (Senzaki et al., 2020). However, models of auditory processing are rarely incorporated into work on acoustic signals. More surprisingly, an understanding of auditory processing in noise is nearly absence from the huge body of literature on the effects of anthropogenic noise on communication and other acoustically mediated behaviors (Fossesca et al., 2023; Francis et al., 2023). Therefore, understanding the differences in critical ratios across species may help us predict how species respond to changes in anthropogenic noise in their environment (Fossesca et al., 2023; Francis et al., 2023).

Critical ratios may be particularly effective at making predictions about behavioral responses to signals that are relatively tonal in noise, as they represent the ability to extract tonal information from background noise. The songs of chickadees and titmice, and to some extent nuthatches, are relatively tonal. Both chickadee and titmouse song consists of several tonal elements between 2 and 3 kHz (Gaddis, 1983; Weisman et al., 1990) whereas the nuthatch song consists of several repeated tonal elements with dominant frequencies between 1.9 and 2.5 kHz (Ritchison, 1983). Nuthatch calls also tend to contain the lowest dominant frequencies of the three species, making them potentially more vulnerable to the effects of masking owing to the low frequencies of anthropogenic noise (Proppe et al., 2013). Even low levels of noise may effectively mask the vocalizations of their own species, making them harder to detect. Because of this, nuthatches may seek out the quieter parts of their habitats. Across the country, white-breasted nuthatches tend to have negative associations with more urbanized areas of development (White, 2013), and even within national parks are approximately 10 times less likely to be found in noisy areas (greater SPL) compared with quieter areas (Goodwin and Shriver, 2010; White, 2013). Nuthatches are less successful in areas of increased urbanization than chickadees and titmice, as seen in parts of Ohio, California and Pennsylvania (Blair, 2004; Reale and Blair, 2005; Paton et al., 2019), and have been shown to have decreased reproductive success with increasing levels of anthropogenic pollutants (Senzaki et al., 2020). In contrast, the effect of these pollutants on titmice have been shown to have minimal effects on reproductive success compared with nuthatches (Senzaki et al., 2020). Some of these differences in urban species presence may be explained by differences in auditory capabilities in the songbirds' physiologies.

We could also use critical ratios to make predictions about the behavior of species that engage in interspecific communication. For instance, differences in auditory processing may lead to asymmetry in the ability of species to engage in mixed-species mobbing behavior as anthropogenic noise levels increase. The three species tested here produce calls within a mobbing context to reduce predation risk (Templeton and Greene, 2007; Courter and Ritchison, 2010; Hetrick and Sieving, 2012), but differences in the perception of noise could alter the propensity of each species to produce or respond to vocalizations. The production of alarm and mobbing calls must be temporally synchronized with the presence of a predator to be effective (Dutour et al., 2016). Therefore, this anti-predator strategy is susceptible to any disruption in the production, propagation or auditory processing of these calls. Differences between species in response to stimuli in noise may thus have unexpectedly complicated effects on interspecific group dynamics. Nuthatches are typically initiators of group mobbing behaviors, and along with chickadees have significantly longer mob durations than titmice in response to predator models (Nolen and Lucas, 2009). However, we found that nuthatches are the most susceptible to masking by anthropogenic noise, which could reduce their ability to initiate mobbing responses. We have previously found noise has the greatest behavioral impact on nuthatch vocal responses but have not recorded how the intensity or duration is affected. However, we could predict that the duration and intensity of nuthatch mobbing may be the most impacted by noise if auditory processing drives behavioral responses. Moreover, these three species tend to show a mutual escalation of mobbing intensity that is dependent on the responses and behaviors of the others, which noise may interfere with (Nolen and Lucas, 2009; Brooks and Freeberg, 2024). Therefore, reduced mobbing behavior by nuthatches, or by nuthatches and chickadees, may have negative downstream effects on the ability of titmice to properly engage in mobbing behavior. Noise thus has the potential to alter the initiation of mobbing events and even the intensity of mobbing, even if the auditory processing of some species in the flock is minimally influenced by noise, ultimately affecting the outcomes of lethal and sublethal predation events. These effects may be compounded by the content of the noise itself, which is also largely ignored in many studies. Embedded in much of the lower amplitude suburban anthropogenic noise is the sound of human speech, which has been shown to have significant impacts on many mammalian species (Clinchy et al., 2016; Suraci et al., 2019; Zanette et al., 2023), although it is less clear the degree to which human speech triggers anti-predator responses in songbirds. Small amounts of noise thus seem to affect both individuals' abilities to perceive sound and the way species interact with one another. Understanding that these differences exist warrants further research into how noise affects animal communication and, consequently, behavior.

### Critical ratio comparative patterns

Auditory processing capabilities are often not considered when discussing the effects of anthropogenic noise on animal communication. When they are considered, it is typically in the broader context that wider auditory filters are mainly responsible for poorer perceptions of signals in noise (Witte et al., 2005; Henry and Lucas, 2010a; Branstetter and Sills, 2022). Additionally, many of these assumptions are based on within-species comparisons rather than across-species comparisons. In humans, for example, wider bandwidths are typically associated with individuals with cochlear damage and impaired hearing (Leek and Summers, 1993; Davies-Venn et al., 2015). In quiet conditions, listeners with broader bandwidths do not appear to have a significant deficit in their thresholds of ability to comprehend speech compared with normal listeners (Sommers and Humes, 1993). However, significant differences emerge in speech perception under noisy conditions, with broader bandwidths corresponding to decreased abilities to process speech (Badri et al., 2011). Within a species, broader filters may be indicative of auditory processing in noise owing to similarities in filter efficiency. However, when making predictions about non-human animals, it is important to consider that across species, both bandwidth and efficiency are changing. Our results suggest that filter efficiency patterns are reflective measures of understanding the effects of noise on auditory processing, at least for these three species. The work presented here is focused on a relatively small sample of species; thus, we avoid making broad claims across all species. However, our data suggest that at a species level, differences in bandwidth alone do not always predict differences in an individual's ability to extract tonal stimuli from noise. Rather, differences in the efficiency of the auditory filters corresponded well with these signal extraction abilities. In birds, hearing loss from peripheral auditory system damage seems to be temporary, as several species have been shown to regenerate and reinnervate hair cells lost to damage, particularly those associated with the lower frequencies used for communication (Marean et al., 1993, 1998; Smolders, 1999; Woolley et al., 2001). Work on budgerigars has similarly suggested that hearing loss due to auditory nerve damage in birds may not result in the same decrements in hearing in noise as found in mammals (Henry and Abrams, 2021; Henry, 2022). These results suggest that the differences in hearing in noise across avian species may not follow the same patterns we would expect based on largely mammalian literature. However, if all species have similar filter efficiencies, it is possible that bandwidth may explain the residual differences in critical ratios. These ideas could, ideally, be tested with a large comparative dataset, both with and without phylogenetic corrections.

The critical ratio by frequency functions of our three species resembled the U-shaped patterns seen in audiograms, contrary to a typical increasing or linear pattern that is seen in most vertebrates. However, critical ratio by frequency functions vary substantially across bird species (reviewed in Fossesca et al., 2023). Within the range of best sensitivity (2–4 kHz), the U-shape was most pronounced in titmice, with chickadees and nuthatches demonstrating relatively shallower patterns. AEP critical ratios of budgerigars, canaries (*Serius canaria*) and zebra finches (*Taeniopygia guttata*) demonstrate similar patterns with the lowest critical ratios and frequencies of best sensitivity (Noirot et al., 2011). Filter efficiency patterns were also mirrored in ABR latencies, which were also shortest in response to higher frequencies in all three species. In birds, ABR latency is typically shortest at best frequencies and increases as you move away from those frequencies (Brittan-Powell et al., 2002; Gall et al., 2012). Additionally, higher frequencies are closest to the base of the membrane, meaning it takes less time for sound to reach these areas and be processed (Geisler, 1998; Rasetshwane et al., 2013). Increasing levels of noise lead to increased latencies in all three species, although chickadees in particular experienced a greater increase in latency in response to 4 kHz tones. This also corresponds with filter efficiency patterns, where chickadees tended to have the worst efficiencies at 4 kHz. Overall, patterns in ABR latencies closely reflected those found in other songbird AEP studies and reflect the general species patterns seen in the other ABR measurements (Henry and Lucas, 2008; Gall et al., 2012; Henry et al., 2016). Filter efficiency patterns best predicted the patterns seen in critical ratios and measurements of hearing in noise, and future work could continue to examine this relationship by testing more songbird species.

We used ABRs, which are non-invasive gross electrophysiological measurements of the peripheral auditory system to the onset of sound (Noirot et al., 2011; Lohr et al., 2013), to estimate critical ratios. This is in contrast to most measurements of songbird critical ratios from psychophysical experiments of captive subjects, which allow for whole-organism responses to sounds the integration of signals over time (Dooling et al., 2000; Brittan-Powell et al., 2002). Careful measurement of the ability to behaviorally detect or discriminate among conspecific vocalizations in noise likely provides the best estimate of the effects on noise on communication for any given species (Pohl et al., 2009, 2012). Psychophysical determination of critical ratios can be more easily compared across species, but are also quite time consuming. Although data are limited, it seems that psychophysical approaches result in critical ratios that are approximately 25–30 dB lower than ABR thresholds and critical ratios (Woolley and Rubel, 1999; Brittan-Powell et al., 2002, 2005). For instance, Noirot et al. (2011) found that zebra finch and budgerigar ABR critical ratios were approximately 40–60 dB, whereas those determined psychophysically were 20–35 dB. However, we found that that our ABR critical ratios were quite similar to the psychophysically determined critical ratios of great tits (20–30 dB; Langemann et al., 1998), the closest relative of chickadees and titmice for which psychophysical critical ratios have been determined. This suggests that ABR critical ratios can provide a first approximation of species-level differences in auditory processing noise and are particularly useful for comparative studies (Fossesca et al., 2023; Francis et al., 2023), even if they remain an imperfect tool for determining the absolute ability to process signals in noise. ABR critical ratios can be determined fairly rapidly and consistently across a wide range of species and wild-caught individuals, which has significant utility in the field of comparative animal hearing. In the future, more studies could combine both psychophysical and AEP studies to both better understand the utilities of the techniques and elucidate the connections between peripheral processing and behavior.

### Conclusions

Species are impacted by anthropogenic stressors in species-specific ways, which is reflected in their sensory systems (Wright et al., 2007; Halfwerk and Slabbekoorn, 2015; Kelley et al., 2018; Michaiel and Bernard, 2022). Yet, our understanding of the ways in which auditory processing mediates responses to anthropogenic noise lags behind our understanding of how visual processing may mediate responses to light pollution (e.g. artificial light at night; see Grubisic et al., 2019; Alaasam et al., 2021; Hussein et al., 2021). However, our results suggest that species, even those engaged in interspecific communication, can differ significantly in their ability to extract information from noise and that these abilities are mirrored in the effects of noise on ecologically relevant behaviors. These data suggest that critical ratios provide a first approximation of hearing in noise that can be used to both compare species with one another and make predictions about the responses of these species to changing anthropogenic environments.

Critical ratios represent a first step for understanding how auditory processing in noise may affect animal behavior. Yet, there is evidence in some model species to suggest that the structure of the noise itself may play an important role in determining how it influences auditory processing and behavior (Langemann and Klump, 2001; Bee et al., 2007; Dooling and Blumenrath, 2016; Pohl et al., 2009). Moreover, signals are very often more complex than pure tones, including in amplitude or temporal modulation, which in turn can affect auditory processing both in quiet and in noise (Woolley and Casseday, 2005; Lucas et al., 2015; Vélez et al., 2015). Building a large comparative dataset of critical ratios can help us make and test predictions about the effects of noise on behavior. However, we should also seek to understand how species differ in other aspects of auditory processing in noise, such as comodulation masking release (Nelken et al., 1999; Erbe, 2008) and modulation filter bank models (Henry, 2023). Although this work might provide more insight into underlying mechanisms responsible for signal processing in variable noise conditions, it is unlikely to be feasible to produce large comparative datasets in these areas at this moment in time. In summary, a thorough understanding of the effects of noise on communication and other acoustically mediated behaviors requires research on both signaler and receiver properties across multiple species. ABR estimates of critical ratios may provide an ideal first step for building comparative datasets to approach these problems.

## Supplementary Material

10.1242/jexbio.250033_sup1 Supplementary information
