# Supplementary material for: I can't hear you: effects of noise on auditory processing in mixed-species flocks
Source: J Exp Biol. 2025 May 22;228(10):jeb250033. doi: 10.1242/jeb.250033 (PMC12148034; doi:10.1242/jeb.250033)
Supplement: Supplementary information [file jexbio-228-250033-s1.pdf]

Table S1. Data for thresholds, critical ratios, amplitudes, and latency minimums and maximums

Date	ID	Species	Sex	Weight	Noise_Level	Frequency	Threshold	Spectrum_Level	Critical_Ratio	Amplitude	Latency_Min	Latency_Max
2/23/23	02594	BCCH	U	12.2	0	1000	32.5	0		3.10366	4.5345	4.0015
2/23/23	02594	BCCH	U	12.2	0	2000	22.5	0		7.010285	4.6575	3.694
2/23/23	02594	BCCH	U	12.2	0	2500	22.5	0		9.66981	4.637	3.817
2/23/23	02594	BCCH	U	12.2	0	3150	17.5	0		6.929195	4.5755	3.776
2/23/23	02594	BCCH	U	12.2	0	4000	22.5	0		3.8672	4.514	3.7555
2/23/23	02594	BCCH	U	12.2	44	1000	37.5	6	31.5	4.934295	5.8055	4.6575
2/23/23	02594	BCCH	U	12.2	44	2000	32.5	6	26.5	6.799245	5.3135	4.391
2/23/23	02594	BCCH	U	12.2	44	2500	32.5	6	26.5	7.10551	5.2725	4.3295
2/23/23	02594	BCCH	U	12.2	44	3150	27.5	6	21.5	6.46587	5.0675	4.2065
2/23/23	02594	BCCH	U	12.2	44	4000	32.5	6	26.5	3.006395	4.9035	4.2475
2/23/23	02594	BCCH	U	12.2	54	1000	42.5	16	26.5	1.71641	4.801	4.063
2/23/23	02594	BCCH	U	12.2	54	2000	37.5	16	21.5	3.1954045	4.5755	3.776
2/23/23	02594	BCCH	U	12.2	54	2500	37.5	16	21.5	3.7002125	4.4115	3.776
2/23/23	02594	BCCH	U	12.2	54	3150	37.5	16	21.5	2.789487	4.514	3.981
2/23/23	02594	BCCH	U	12.2	54	4000	37.5	16	21.5	2.525254	4.473	3.694
2/23/23	02594	BCCH	U	12.2	64	1000	57.5	26	31.5	1.319006	4.5755	3.94
2/23/23	02594	BCCH	U	12.2	64	2000	47.5	26	21.5	3.0006925	4.842	3.7965
2/23/23	02594	BCCH	U	12.2	64	2500	47.5	26	21.5	2.793987	4.6985	4.0015
2/23/23	02594	BCCH	U	12.2	64	3150	42.5	26	16.5	2.7077575	4.76	4.0015
2/23/23	02594	BCCH	U	12.2	64	4000	52.5	26	26.5	1.517846	4.719	3.9195
2/23/23	02595	BCCH	U	21.3	0	1000	32.5	0		4.243595	5.129	3.817
2/23/23	02595	BCCH	U	21.3	0	2000	27.5	0		6.52995	4.8625	3.9195
2/23/23	02595	BCCH	U	21.3	0	2500	27.5	0		4.5649955	4.7395	4.022
2/23/23	02595	BCCH	U	21.3	0	3150	22.5	0		4.984507	4.8215	4.186
2/23/23	02595	BCCH	U	21.3	0	4000	27.5	0		3.106344	4.596	4.0015
2/23/23	02595	BCCH	U	21.3	44	1000	37.5	6	31.5	4.8191685	5.3955	3.53
2/23/23	02595	BCCH	U	21.3	44	2000	32.5	6	26.5	4.44423	5.088	3.8785
2/23/23	02595	BCCH	U	21.3	44	2500	27.5	6	21.5	5.168835	5.129	3.9195
2/23/23	02595	BCCH	U	21.3	44	3150	27.5	6	21.5	4.588706	5.2315	3.858
2/23/23	02595	BCCH	U	21.3	44	4000	32.5	6	26.5	1.7107785	4.391	3.94
2/23/23	02595	BCCH	U	21.3	54	1000	42.5	16	26.5	3.7019415	4.924	3.8375
2/23/23	02595	BCCH	U	21.3	54	2000	37.5	16	21.5			
2/23/23	02595	BCCH	U	21.3	54	2500	32.5	16	16.5	3.25708	5.1085	3.8375
2/23/23	02595	BCCH	U	21.3	54	3150	37.5	16	21.5	3.4984435	4.8625	3.899
2/23/23	02595	BCCH	U	21.3	54	4000	42.5	16	26.5	3.9179095	7.1585	5.7645
2/23/23	02595	BCCH	U	21.3	64	1000	57.5	26	31.5	3.2509825	5.2315	3.981
2/23/23	02595	BCCH	U	21.3	64	2000	52.5	26	26.5	2.4025	4.5755	3.817
2/23/23	02595	BCCH	U	21.3	64	2500	47.5	26	21.5			
2/23/23	02595	BCCH	U	21.3	64	3150	52.5	26	26.5	2.705737	4.0015	3.858
2/23/23	02595	BCCH	U	21.3	64	4000	52.5	26	26.5	2.0306166	6.4615	6.2565
2/28/23	02596	BCCH	U	10	0	1000	37.5	0		5.298905	5.006	3.981
2/28/23	02596	BCCH	U	10	0	2000	27.5	0		9.25869	4.842	4.063
2/28/23	02596	BCCH	U	10	0	2500	22.5	0		13.46102	4.801	3.9195
2/28/23	02596	BCCH	U	10	0	3150	22.5	0		13.342865	5.1905	4.0015
2/28/23	02596	BCCH	U	10	0	4000	42.5	0		4.086265	5.2315	3.858
2/28/23	02596	BCCH	U	10	44	1000	37.5	6	31.5	4.1584105	5.375	3.776
2/28/23	02596	BCCH	U	10	44	2000	27.5	6	21.5	5.36747	4.842	3.7145
2/28/23	02596	BCCH	U	10	44	2500	32.5	6	26.5	5.809945	4.5345	3.817
2/28/23	02596	BCCH	U	10	44	3150	27.5	6	21.5	4.065073	4.6165	3.8785
2/28/23	02596	BCCH	U	10	44	4000	32.5	6	26.5	1.914676	4.4115	3.4685
2/28/23	02596	BCCH	U	10	54	1000	37.5	16	21.5	3.1989635	6.851	6.687
2/28/23	02596	BCCH	U	10	54	2000	32.5	16	16.5	3.6121	5.3135	3.899
2/28/23	02596	BCCH	U	10	54	2500	32.5	16	16.5	3.12937	4.596	4.0015
2/28/23	02596	BCCH	U	10	54	3150	37.5	16	21.5	3.17903	4.6985	3.8375
2/28/23	02596	BCCH	U	10	54	4000	37.5	16	21.5	4.3419595	7.4045	6.1335
2/28/23	02596	BCCH	U	10	64	1000	52.5	26	26.5	1.8287945	5.457	4.1245
2/28/23	02596	BCCH	U	10	64	2000	47.5	26	21.5	4.3372955	5.5595	3.6735

2/28/23	02596	BCCH	U	10	64	2500	42.5	26	16.5	2.9475789	4.022	2.7305
2/28/23	02596	BCCH	U	10	64	3150	42.5	26	16.5	2.976124	4.514	3.325
2/28/23	02596	BCCH	U	10	64	4000	47.5	26	21.5	4.22621	6.236	4.514
2/28/23	02597	BCCH	U	11.3	0	1000	27.5	0		3.81113	4.555	3.9195
2/28/23	02597	BCCH	U	11.3	0	2000	27.5	0		7.95006	4.514	3.7145
2/28/23	02597	BCCH	U	11.3	0	2500	17.5	0		8.511155	4.5345	3.6735
2/28/23	02597	BCCH	U	11.3	0	3150	27.5	0		6.1615	4.4935	3.694
2/28/23	02597	BCCH	U	11.3	0	4000	27.5	0		2.611839	4.6985	3.612
2/28/23	02597	BCCH	U	11.3	44	1000	32.5	6	26.5	3.643925	4.7395	3.489
2/28/23	02597	BCCH	U	11.3	44	2000	32.5	6	26.5	5.459595	4.678	3.4685
2/28/23	02597	BCCH	U	11.3	44	2500	27.5	6	21.5	4.43382	4.432	3.694
2/28/23	02597	BCCH	U	11.3	44	3150	32.5	6	26.5	3.6170245	4.4525	3.3865
2/28/23	02597	BCCH	U	11.3	44	4000	37.5	6	31.5	1.9341305	3.9195	3.5505
2/28/23	02597	BCCH	U	11.3	54	1000	42.5	16	26.5	2.7287895	5.0265	4.0425
2/28/23	02597	BCCH	U	11.3	54	2000	47.5	16	31.5	3.85362	4.5755	3.735
2/28/23	02597	BCCH	U	11.3	54	2500	42.5	16	26.5	3.419655	4.637	3.612
2/28/23	02597	BCCH	U	11.3	54	3150	37.5	16	21.5	2.6750835	4.7805	3.571
2/28/23	02597	BCCH	U	11.3	54	4000	42.5	16	26.5	1.4876815	4.883	3.9605
2/28/23	02597	BCCH	U	11.3	64	1000	52.5	26	26.5	2.411406	5.375	3.94
2/28/23	02597	BCCH	U	11.3	64	2000	47.5	26	21.5	2.78408	4.76	3.9605
2/28/23	02597	BCCH	U	11.3	64	2500	47.5	26	21.5	2.69864	4.8215	3.981
2/28/23	02597	BCCH	U	11.3	64	3150	52.5	26	26.5	2.504443	5.17	3.9195
2/28/23	02597	BCCH	U	11.3	64	4000	47.5	26	21.5	1.6847821	5.662	4.76
3/2/23	02598	BCCH	U	10.1	0	1000	47.5	0		3.2332205	5.539	4.2885
3/2/23	02598	BCCH	U	10.1	0	2000	37.5	0		3.8217555	5.3545	4.35
3/2/23	02598	BCCH	U	10.1	0	2500	42.5	0		3.743725	5.129	4.432
3/2/23	02598	BCCH	U	10.1	0	3150	42.5	0		4.997955	5.1495	4.309
3/2/23	02598	BCCH	U	10.1	0	4000	37.5	0		3.070803	4.924	4.227
3/2/23	02598	BCCH	U	10.1	44	1000	47.5	6	41.5	2.8663495	5.5185	4.555
3/2/23	02598	BCCH	U	10.1	44	2000	42.5	6	36.5	4.543512	5.1495	4.2475
3/2/23	02598	BCCH	U	10.1	44	2500	42.5	6	36.5	4.73998	4.924	4.227
3/2/23	02598	BCCH	U	10.1	44	3150	37.5	6	31.5	4.697255	4.924	4.0835
3/2/23	02598	BCCH	U	10.1	44	4000	37.5	6	31.5	3.130743	4.76	4.0835
3/2/23	02598	BCCH	U	10.1	54	1000	47.5	16	31.5	2.6876157	5.498	3.8785
3/2/23	02598	BCCH	U	10.1	54	2000	47.5	16	31.5	2.465871	4.924	4.2475
3/2/23	02598	BCCH	U	10.1	54	2500	47.5	16	31.5	2.732105	4.9035	4.3295
3/2/23	02598	BCCH	U	10.1	54	3150	42.5	16	26.5	3.388225	4.924	4.0015
3/2/23	02598	BCCH	U	10.1	54	4000	42.5	16	26.5	2.3749105	4.637	4.063
3/2/23	02598	BCCH	U	10.1	64	1000	62.5	26	36.5	1.8333795	6.9945	5.867
3/2/23	02598	BCCH	U	10.1	64	2000	62.5	26	36.5	1.392395	5.58	5.416
3/2/23	02598	BCCH	U	10.1	64	2500	52.5	26	26.5	1.6598182	5.088	4.4115
3/2/23	02598	BCCH	U	10.1	64	3150	52.5	26	26.5	1.6336845	4.883	4.4115
3/2/23	02598	BCCH	U	10.1	64	4000	52.5	26	26.5	1.853716	5.047	4.1245
3/21/23	02599	BCCH	M	10.9	0	1000	52.5	0		1.7773425	5.785	4.9035
3/21/23	02599	BCCH	M	10.9	0	2000	42.5	0		3.456445	5.375	4.4935
3/21/23	02599	BCCH	M	10.9	0	2500	42.5	0		3.097335	5.3545	4.35
3/21/23	02599	BCCH	M	10.9	0	3150	42.5	0		4.081145	5.0675	4.309
3/21/23	02599	BCCH	M	10.9	0	4000	32.5	0		1.5978695	5.088	4.35
3/21/23	02599	BCCH	M	10.9	44	1000	52.5	6	46.5	4.73616	5.6415	4.0015
3/21/23	02599	BCCH	M	10.9	44	2000	42.5	6	36.5	4.59778	4.8215	3.7965
3/21/23	02599	BCCH	M	10.9	44	2500	42.5	6	36.5	3.5378125	4.7805	3.8785
3/21/23	02599	BCCH	M	10.9	44	3150	42.5	6	36.5	2.9003565	4.965	3.8785
3/21/23	02599	BCCH	M	10.9	44	4000	42.5	6	36.5	1.848562	4.842	4.514
3/21/23	02599	BCCH	M	10.9	54	1000	57.5	16	41.5	2.72931	5.416	4.1655
3/21/23	02599	BCCH	M	10.9	54	2000	47.5	16	31.5	2.0311435	4.883	3.8785
3/21/23	02599	BCCH	M	10.9	54	2500	47.5	16	31.5	2.88881	4.76	3.7555
3/21/23	02599	BCCH	M	10.9	54	3150	47.5	16	31.5	2.8164	4.76	3.9195
3/21/23	02599	BCCH	M	10.9	54	4000	47.5	16	31.5	1.140085	4.842	4.8625

3/21/23	02599	BCCH	M	10.9	64	1000	67.5	26	41.5	1.387613	5.17	4.063
3/21/23	02599	BCCH	M	10.9	64	2000	62.5	26	36.5	1.143783	4.8625	3.899
3/21/23	02599	BCCH	M	10.9	64	2500	52.5	26	26.5	2.415173	5.129	4.2065
3/21/23	02599	BCCH	M	10.9	64	3150	62.5	26	36.5	1.9365575	5.0265	3.653
3/21/23	02599	BCCH	M	10.9	64	4000	57.5	26	31.5	0.9697452	4.6985	4.4935
2/7/23	06664	TUTI	U	21.9	0	1000	37.5	0		2.5656115	5.8875	4.8215
2/7/23	06664	TUTI	U	21.9	0	2000	32.5	0		5.45426	5.457	4.309
2/7/23	06664	TUTI	U	21.9	0	2500	32.5	0		6.24062	5.293	4.268
2/7/23	06664	TUTI	U	21.9	0	3150	22.5	0		5.277765	5.006	3.9605
2/7/23	06664	TUTI	U	21.9	0	4000	27.5	0		2.5158545	4.842	4.063
2/7/23	06664	TUTI	U	21.9	44	1000	42.5	6	36.5	1.728343	4.555	3.612
2/7/23	06664	TUTI	U	21.9	44	2000	32.5	6	26.5	3.5116605	4.76	3.448
2/7/23	06664	TUTI	U	21.9	44	2500	37.5	6	31.5	4.303605	4.5755	3.3865
2/7/23	06664	TUTI	U	21.9	44	3150	32.5	6	26.5	3.492969	4.3295	3.407
2/7/23	06664	TUTI	U	21.9	44	4000	32.5	6	26.5	2.767007	4.227	3.407
2/7/23	06664	TUTI	U	21.9	54	1000	47.5	16	31.5	1.808515	5.129	4.0425
2/7/23	06664	TUTI	U	21.9	54	2000	42.5	16	26.5	2.631374	4.883	3.7555
2/7/23	06664	TUTI	U	21.9	54	2500	37.5	16	21.5			
2/7/23	06664	TUTI	U	21.9	54	3150	32.5	16	16.5			
2/7/23	06664	TUTI	U	21.9	54	4000	32.5	16	16.5	1.6602175	4.309	3.6735
2/7/23	06664	TUTI	U	21.9	64	1000	62.5	26	36.5	0.7140074	4.6985	3.981
2/7/23	06664	TUTI	U	21.9	64	2000	52.5	26	26.5	1.4032315	4.924	3.53
2/7/23	06664	TUTI	U	21.9	64	2500	42.5	26	16.5	1.955476	4.596	3.489
2/7/23	06664	TUTI	U	21.9	64	3150	47.5	26	21.5	1.753894	4.678	3.5095
2/7/23	06664	TUTI	U	21.9	64	4000	47.5	26	21.5	1.2631625	4.3705	3.817
2/7/23	06665	TUTI	U	23.9	0	1000	27.5	0		3.703515	5.0675	3.94
2/7/23	06665	TUTI	U	23.9	0	2000	22.5	0				
2/7/23	06665	TUTI	U	23.9	0	2500	22.5	0				
2/7/23	06665	TUTI	U	23.9	0	3150	22.5	0				
2/7/23	06665	TUTI	U	23.9	0	4000	27.5	0		2.935915	4.5755	3.8375
2/7/23	06665	TUTI	U	23.9	44	1000	37.5	6	31.5	2.471484	5.006	3.653
2/7/23	06665	TUTI	U	23.9	44	2000	27.5	6	21.5			
2/7/23	06665	TUTI	U	23.9	44	2500	27.5	6	21.5			
2/7/23	06665	TUTI	U	23.9	44	3150	27.5	6	21.5			
2/7/23	06665	TUTI	U	23.9	44	4000	27.5	6	21.5	2.0527995	4.2885	3.5915
2/7/23	06665	TUTI	U	23.9	54	1000	42.5	16	26.5			
2/7/23	06665	TUTI	U	23.9	54	2000	42.5	16	26.5			
2/7/23	06665	TUTI	U	23.9	54	2500	37.5	16	21.5			
2/7/23	06665	TUTI	U	23.9	54	3150	32.5	16	16.5			
2/7/23	06665	TUTI	U	23.9	54	4000	42.5	16	26.5	1.3206678	4.2475	3.5915
2/7/23	06665	TUTI	U	23.9	64	1000	57.5	26	31.5			
2/7/23	06665	TUTI	U	23.9	64	2000	57.5	26	31.5	2.2592155	4.5755	3.653
2/7/23	06665	TUTI	U	23.9	64	2500	47.5	26	21.5			
2/7/23	06665	TUTI	U	23.9	64	3150	47.5	26	21.5			
2/7/23	06665	TUTI	U	23.9	64	4000	52.5	26	26.5	0.9700095	4.309	3.7555
2/14/23	06666	TUTI	U	23.5	0	1000	37.5	0		2.7757025	4.4115	3.6735
2/14/23	06666	TUTI	U	23.5	0	2000	27.5	0		6.27423	4.473	3.612
2/14/23	06666	TUTI	U	23.5	0	2500	22.5	0		9.127645	4.4935	3.6325
2/14/23	06666	TUTI	U	23.5	0	3150	17.5	0		7.42508	4.391	3.4685
2/14/23	06666	TUTI	U	23.5	0	4000	17.5	0		4.90313	4.1655	3.284
2/14/23	06666	TUTI	U	23.5	44	1000	37.5	6	31.5	2.373549	4.883	3.735
2/14/23	06666	TUTI	U	23.5	44	2000	27.5	6	21.5	7.041875	4.4935	3.5915
2/14/23	06666	TUTI	U	23.5	44	2500	27.5	6	21.5			
2/14/23	06666	TUTI	U	23.5	44	3150	22.5	6	16.5			
2/14/23	06666	TUTI	U	23.5	44	4000	22.5	6	16.5	3.162045	4.309	3.407
2/14/23	06666	TUTI	U	23.5	54	1000	47.5	16	31.5	3.63835	5.58	4.473
2/14/23	06666	TUTI	U	23.5	54	2000	42.5	16	26.5	5.114895	5.088	4.063
2/14/23	06666	TUTI	U	23.5	54	2500	37.5	16	21.5	7.113025	5.006	3.899

2/14/23	06666	TUTI	U	23.5	54	3150	32.5	16	16.5	6.472775	4.842	3.817
2/14/23	06666	TUTI	U	23.5	54	4000	37.5	16	21.5	2.6352475	4.6985	3.817
2/14/23	06666	TUTI	U	23.5	64	1000	52.5	26	26.5	1.429601	5.3955	4.3705
2/14/23	06666	TUTI	U	23.5	64	2000	52.5	26	26.5	1.4333515	4.6985	4.0425
2/14/23	06666	TUTI	U	23.5	64	2500	47.5	26	21.5	3.0459905	4.842	3.981
2/14/23	06666	TUTI	U	23.5	64	3150	47.5	26	21.5	2.8157748	4.883	3.776
2/14/23	06666	TUTI	U	23.5	64	4000	47.5	26	21.5	1.879113	4.678	3.858
2/14/23	06667	TUTI	U	21.4	0	1000	22.5	0		5.17858	5.088	3.8375
2/14/23	06667	TUTI	U	21.4	0	2000	27.5	0		8.51516	4.9855	3.899
2/14/23	06667	TUTI	U	21.4	0	2500	22.5	0		7.755565	4.883	3.8785
2/14/23	06667	TUTI	U	21.4	0	3150	22.5	0		6.09146	4.9035	3.94
2/14/23	06667	TUTI	U	21.4	0	4000	27.5	0		3.685725	4.637	3.735
2/14/23	06667	TUTI	U	21.4	44	1000	32.5	6	26.5	2.37858	4.678	3.325
2/14/23	06667	TUTI	U	21.4	44	2000	27.5	6	21.5	3.961625	4.8625	3.6325
2/14/23	06667	TUTI	U	21.4	44	2500	27.5	6	21.5	4.393155	4.7805	3.571
2/14/23	06667	TUTI	U	21.4	44	3150	22.5	6	16.5	4.73733	4.5345	3.5505
2/14/23	06667	TUTI	U	21.4	44	4000	27.5	6	21.5	2.79369	4.268	3.53
2/14/23	06667	TUTI	U	21.4	54	1000	42.5	16	26.5	1.8314615	4.883	3.776
2/14/23	06667	TUTI	U	21.4	54	2000	47.5	16	31.5	2.393377	4.924	3.735
2/14/23	06667	TUTI	U	21.4	54	2500	37.5	16	21.5	3.62689	4.7805	3.6325
2/14/23	06667	TUTI	U	21.4	54	3150	37.5	16	21.5	3.44429	4.473	3.7145
2/14/23	06667	TUTI	U	21.4	54	4000	37.5	16	21.5	2.436493	4.2065	3.4685
2/14/23	06667	TUTI	U	21.4	64	1000	52.5	26	26.5	1.4438765	5.1085	3.899
2/14/23	06667	TUTI	U	21.4	64	2000	52.5	26	26.5	1.7665955	4.7805	3.653
2/14/23	06667	TUTI	U	21.4	64	2500	47.5	26	21.5	2.60274	4.9445	3.776
2/14/23	06667	TUTI	U	21.4	64	3150	47.5	26	21.5	2.70017	4.7395	3.9195
2/14/23	06667	TUTI	U	21.4	64	4000	47.5	26	21.5	1.5955835	4.5755	4.0015
2/16/23	06668	TUTI	U	21.3	0	1000	27.5	0		3.137025	4.678	3.9195
2/16/23	06668	TUTI	U	21.3	0	2000	27.5	0		5.966365	4.76	3.817
2/16/23	06668	TUTI	U	21.3	0	2500	22.5	0		7.01012	4.5755	3.7555
2/16/23	06668	TUTI	U	21.3	0	3150	22.5	0		5.68984	4.678	3.858
2/16/23	06668	TUTI	U	21.3	0	4000	22.5	0		2.6687785	4.391	3.694
2/16/23	06668	TUTI	U	21.3	44	1000	37.5	6	31.5	1.6498915	4.5755	3.858
2/16/23	06668	TUTI	U	21.3	44	2000	27.5	6	21.5	1.3474501	4.145	3.653
2/16/23	06668	TUTI	U	21.3	44	2500	27.5	6	21.5	3.361829	4.2885	3.694
2/16/23	06668	TUTI	U	21.3	44	3150	27.5	6	21.5	3.43319	4.2065	3.53
2/16/23	06668	TUTI	U	21.3	44	4000	32.5	6	26.5	1.6752555	3.981	3.5095
2/16/23	06668	TUTI	U	21.3	54	1000	47.5	16	31.5	1.5851185	4.7395	3.7145
2/16/23	06668	TUTI	U	21.3	54	2000	47.5	16	31.5	2.0780645	4.7395	3.6735
2/16/23	06668	TUTI	U	21.3	54	2500	37.5	16	21.5	2.5775815	4.4525	3.858
2/16/23	06668	TUTI	U	21.3	54	3150	32.5	16	16.5	3.266705	4.35	3.694
2/16/23	06668	TUTI	U	21.3	54	4000	42.5	16	26.5	1.561662	4.104	3.5915
2/16/23	06668	TUTI	U	21.3	64	1000	52.5	26	26.5	0.8274225	4.76	4.063
2/16/23	06668	TUTI	U	21.3	64	2000	52.5	26	26.5	1.4971185	4.637	3.7965
2/16/23	06668	TUTI	U	21.3	64	2500	42.5	26	16.5	2.9608978	4.473	3.7965
2/16/23	06668	TUTI	U	21.3	64	3150	47.5	26	21.5	2.5127795	4.514	3.694
2/16/23	06668	TUTI	U	21.3	64	4000	47.5	26	21.5	1.1394695	4.4935	4.3295
2/16/23	06669	TUTI	U	19.9	0	1000	32.5	0		3.4521745	4.514	4.186
2/16/23	06669	TUTI	U	19.9	0	2000	27.5	0		6.53789	4.6165	4.0425
2/16/23	06669	TUTI	U	19.9	0	2500	27.5	0		10.066085	4.555	3.7965
2/16/23	06669	TUTI	U	19.9	0	3150	22.5	0		9.98462	4.432	3.6735
2/16/23	06669	TUTI	U	19.9	0	4000	22.5	0		4.922484	4.309	3.612
2/16/23	06669	TUTI	U	19.9	44	1000	37.5	6	31.5	2.2846315	4.6985	3.9195
2/16/23	06669	TUTI	U	19.9	44	2000	32.5	6	26.5	3.34579	4.6575	3.899
2/16/23	06669	TUTI	U	19.9	44	2500	22.5	6	16.5	4.9324935	4.473	3.7965
2/16/23	06669	TUTI	U	19.9	44	3150	22.5	6	16.5	4.4749065	4.268	3.694
2/16/23	06669	TUTI	U	19.9	44	4000	32.5	6	26.5	3.3047485	4.2065	3.5915
2/16/23	06669	TUTI	U	19.9	54	1000	47.5	16	31.5	1.171015	4.596	3.94

2/16/23	06669	TUTI	U	19.9	54	2000	42.5	16	26.5	2.688246	4.7395	3.8375
2/16/23	06669	TUTI	U	19.9	54	2500	37.5	16	21.5	2.948965	4.391	3.694
2/16/23	06669	TUTI	U	19.9	54	3150	32.5	16	16.5	2.839707	4.309	3.7555
2/16/23	06669	TUTI	U	19.9	54	4000	37.5	16	21.5	2.385706	4.227	3.7555
2/16/23	06669	TUTI	U	19.9	64	1000	52.5	26	26.5	1.069959	4.637	4.0425
2/16/23	06669	TUTI	U	19.9	64	2000	47.5	26	21.5	2.2347255	4.8625	4.0425
2/16/23	06669	TUTI	U	19.9	64	2500	47.5	26	21.5	2.3537666	4.883	3.981
2/16/23	06669	TUTI	U	19.9	64	3150	47.5	26	21.5	2.610384	4.637	3.858
2/16/23	06669	TUTI	U	19.9	64	4000	47.5	26	21.5	0.9569075	4.5345	4.0425
2/21/23	06670	TUTI	U	21.3	0	1000	27.5	0		2.677782	4.924	4.0425
2/21/23	06670	TUTI	U	21.3	0	2000	22.5	0		7.607485	4.555	3.6325
2/21/23	06670	TUTI	U	21.3	0	2500	22.5	0		7.99771	4.6165	3.694
2/21/23	06670	TUTI	U	21.3	0	3150	17.5	0		7.809125	4.391	3.5915
2/21/23	06670	TUTI	U	21.3	0	4000	22.5	0		4.646285	4.3295	3.571
2/21/23	06670	TUTI	U	21.3	44	1000	37.5	6	31.5	3.5914905	5.4365	4.473
2/21/23	06670	TUTI	U	21.3	44	2000	37.5	6	31.5	5.768395	5.0265	4.0015
2/21/23	06670	TUTI	U	21.3	44	2500	27.5	6	21.5	7.572385	4.9035	3.94
2/21/23	06670	TUTI	U	21.3	44	3150	22.5	6	16.5	5.902895	4.637	3.7555
2/21/23	06670	TUTI	U	21.3	44	4000	27.5	6	21.5	3.15239	4.473	3.612
2/21/23	06670	TUTI	U	21.3	54	1000	47.5	16	31.5	3.171478	5.6005	4.4525
2/21/23	06670	TUTI	U	21.3	54	2000	32.5	16	16.5	4.537265	5.1495	4.1655
2/21/23	06670	TUTI	U	21.3	54	2500	32.5	16	16.5	5.99113	5.088	4.022
2/21/23	06670	TUTI	U	21.3	54	3150	32.5	16	16.5	4.27247	4.6985	3.94
2/21/23	06670	TUTI	U	21.3	54	4000	37.5	16	21.5	2.347105	4.5755	3.8375
2/21/23	06670	TUTI	U	21.3	64	1000	52.5	26	26.5	1.88868	5.4365	4.1655
2/21/23	06670	TUTI	U	21.3	64	2000	47.5	26	21.5	1.659	4.8215	4.063
2/21/23	06670	TUTI	U	21.3	64	2500	42.5	26	16.5	3.05097	4.883	3.8785
2/21/23	06670	TUTI	U	21.3	64	3150	42.5	26	16.5	2.1267075	4.6165	3.7965
2/21/23	06670	TUTI	U	21.3	64	4000	42.5	26	16.5	1.7029125	4.3295	3.7555
3/23/23	06663	WBNU	M	20.6	0	1000	22.5	0		5.19189	5.17	4.104
3/23/23	06663	WBNU	M	20.6	0	2000	7.5	0		9.35908	4.8625	3.7145
3/23/23	06663	WBNU	M	20.6	0	2500	12.5	0		8.871695	4.9855	3.7555
3/23/23	06663	WBNU	M	20.6	0	3150	17.5	0		5.68224	4.842	3.612
3/23/23	06663	WBNU	M	20.6	0	4000	22.5	0		2.94743	4.7395	3.776
3/23/23	06663	WBNU	M	20.6	44	1000	32.5	6	26.5	2.715185	5.334	4.063
3/23/23	06663	WBNU	M	20.6	44	2000	32.5	6	26.5	3.42808	5.17	3.7555
3/23/23	06663	WBNU	M	20.6	44	2500	32.5	6	26.5	3.396615	5.0265	3.7145
3/23/23	06663	WBNU	M	20.6	44	3150	37.5	6	31.5	2.073716	4.883	3.7965
3/23/23	06663	WBNU	M	20.6	44	4000	37.5	6	31.5	1.0165143	4.4935	3.735
3/23/23	06663	WBNU	M	20.6	54	1000	52.5	16	36.5	2.208562	5.1905	4.104
3/23/23	06663	WBNU	M	20.6	54	2000	52.5	16	36.5	2.3833415	5.0265	3.899
3/23/23	06663	WBNU	M	20.6	54	2500	52.5	16	36.5	2.404201	5.1495	3.7145
3/23/23	06663	WBNU	M	20.6	54	3150	52.5	16	36.5	1.513441	4.801	3.8375
3/23/23	06663	WBNU	M	20.6	54	4000	47.5	16	31.5	1.056999	4.801	3.94
3/23/23	06663	WBNU	M	20.6	64	1000	57.5	26	31.5	1.114063	5.375	4.3295
3/23/23	06663	WBNU	M	20.6	64	2000	57.5	26	31.5	1.1507555	4.7805	4.145
3/23/23	06663	WBNU	M	20.6	64	2500	57.5	26	31.5	1.164966	4.965	4.1655
3/23/23	06663	WBNU	M	20.6	64	3150	62.5	26	36.5	0.959227	4.596	4.1245
3/23/23	06663	WBNU	M	20.6	64	4000	57.5	26	31.5	0.6545365	4.883	4.104
3/2/23	06671	WBNU	F	21.5	0	1000	27.5	0		4.28141	4.9855	3.981
3/2/23	06671	WBNU	F	21.5	0	2000	17.5	0		8.48479	4.7805	3.8785
3/2/23	06671	WBNU	F	21.5	0	2500	17.5	0		8.41885	4.7805	3.817
3/2/23	06671	WBNU	F	21.5	0	3150	12.5	0		5.796085	4.719	3.7965
3/2/23	06671	WBNU	F	21.5	0	4000	17.5	0		2.482728	4.4115	3.8375
3/2/23	06671	WBNU	F	21.5	44	1000	42.5	6	36.5	2.6214665	5.1085	3.96
3/2/23	06671	WBNU	F	21.5	44	2000	32.5	6	26.5	3.3710735	4.9855	3.92
3/2/23	06671	WBNU	F	21.5	44	2500	32.5	6	26.5	3.7486	4.637	3.8
3/2/23	06671	WBNU	F	21.5	44	3150	32.5	6	26.5	2.8226345	4.637	3.78

3/2/23	06671	WBNU	F	21.5	44	4000	37.5	6	31.5	1.3665089	4.35	3.612
3/2/23	06671	WBNU	F	21.5	54	1000	57.5	16	41.5	2.232535	5.1085	4.7
3/2/23	06671	WBNU	F	21.5	54	2000	42.5	16	26.5	2.5685815	5.211	4.06
3/2/23	06671	WBNU	F	21.5	54	2500	47.5	16	31.5	3.308964	4.801	3.2
3/2/23	06671	WBNU	F	21.5	54	3150	47.5	16	31.5	3.564579	5.1085	3.899
3/2/23	06671	WBNU	F	21.5	54	4000	47.5	16	31.5	1.172579	4.6165	3.8
3/2/23	06671	WBNU	F	21.5	64	1000	62.5	26	36.5	1.1072605	5.2725	4.51
3/2/23	06671	WBNU	F	21.5	64	2000	57.5	26	31.5	1.9490655	4.7395	3.98
3/2/23	06671	WBNU	F	21.5	64	2500	52.5	26	26.5	1.2818015	4.9445	3.9
3/2/23	06671	WBNU	F	21.5	64	3150	57.5	26	31.5	1.8105795	4.8625	4.21
3/2/23	06671	WBNU	F	21.5	64	4000	62.5	26	36.5	1.0079815	4.637	3.88
3/21/23	06672	WBNU	M	20.6	0	1000	7.5	0		6.099635	5.375	3.5505
3/21/23	06672	WBNU	M	20.6	0	2000	12.5	0		8.292375	5.4365	3.817
3/21/23	06672	WBNU	M	20.6	0	2500	12.5	0		8.428835	5.293	3.8785
3/21/23	06672	WBNU	M	20.6	0	3150	17.5	0		5.173575	5.5185	3.9605
3/21/23	06672	WBNU	M	20.6	0	4000	32.5	0		1.6949384	5.457	3.8785
3/21/23	06672	WBNU	M	20.6	44	1000	37.5	6	31.5	2.1748775	5.785	3.653
3/21/23	06672	WBNU	M	20.6	44	2000	32.5	6	26.5	2.9816035	5.375	3.858
3/21/23	06672	WBNU	M	20.6	44	2500	32.5	6	26.5	3.45832	5.2725	3.8375
3/21/23	06672	WBNU	M	20.6	44	3150	37.5	6	31.5	2.0754405	5.3955	4.104
3/21/23	06672	WBNU	M	20.6	44	4000	37.5	6	31.5	1.64687	5.293	4.268
3/21/23	06672	WBNU	M	20.6	54	1000	47.5	16	31.5	1.7959715	5.8875	4.5755
3/21/23	06672	WBNU	M	20.6	54	2000	37.5	16	21.5	1.5633905	5.334	3.8785
3/21/23	06672	WBNU	M	20.6	54	2500	42.5	16	26.5	2.743345	5.498	4.0015
3/21/23	06672	WBNU	M	20.6	54	3150	42.5	16	26.5	1.5421185	5.1085	3.858
3/21/23	06672	WBNU	M	20.6	54	4000	47.5	16	31.5	0.550829	4.7395	4.2885
3/21/23	06672	WBNU	M	20.6	64	1000	57.5	26	31.5	0.770531	4.5345	3.5505
3/21/23	06672	WBNU	M	20.6	64	2000	57.5	26	31.5	1.3731875	4.3705	4.186
3/21/23	06672	WBNU	M	20.6	64	2500	57.5	26	31.5	1.658774	4.9855	4.0015
3/21/23	06672	WBNU	M	20.6	64	3150	52.5	26	26.5	1.379389	3.0995	2.546
3/21/23	06672	WBNU	M	20.6	64	4000	57.5	26	31.5	0.8394865	4.391	3.7145
3/30/23	06673	WBNU	F	20.6	0	1000	22.5	0		4.390445	5.58	4.227
3/30/23	06673	WBNU	F	20.6	0	2000	22.5	0		7.263915	5.252	4.1655
3/30/23	06673	WBNU	F	20.6	0	2500	17.5	0		6.19344	5.293	4.1245
3/30/23	06673	WBNU	F	20.6	0	3150	12.5	0		3.591925	4.924	3.8785
3/30/23	06673	WBNU	F	20.6	0	4000	17.5	0		1.6863733	4.7805	4.0425
3/30/23	06673	WBNU	F	20.6	44	1000	32.5	6	26.5	2.329129	5.8465	4.35
3/30/23	06673	WBNU	F	20.6	44	2000	37.5	6	31.5	3.206755	4.842	4.022
3/30/23	06673	WBNU	F	20.6	44	2500	32.5	6	26.5	2.893165	4.842	3.858
3/30/23	06673	WBNU	F	20.6	44	3150	32.5	6	26.5	2.055761	4.7395	3.899
3/30/23	06673	WBNU	F	20.6	44	4000	32.5	6	26.5	1.0954188	4.473	3.776
3/30/23	06673	WBNU	F	20.6	54	1000	47.5	16	31.5	1.273264	5.457	4.2475
3/30/23	06673	WBNU	F	20.6	54	2000	42.5	16	26.5	1.7915505	4.9855	4.104
3/30/23	06673	WBNU	F	20.6	54	2500	42.5	16	26.5	2.271335	5.0265	4.0015
3/30/23	06673	WBNU	F	20.6	54	3150	42.5	16	26.5	1.660605	5.1085	3.8785
3/30/23	06673	WBNU	F	20.6	54	4000	47.5	16	31.5	1.0926545	4.7805	3.694
3/30/23	06673	WBNU	F	20.6	64	1000	62.5	26	36.5	0.8876515	6.0515	4.6165
3/30/23	06673	WBNU	F	20.6	64	2000	57.5	26	31.5	1.284671	5.457	4.4115
3/30/23	06673	WBNU	F	20.6	64	2500	52.5	26	26.5	1.720284	5.1085	3.94
3/30/23	06673	WBNU	F	20.6	64	3150	52.5	26	26.5	1.1301655	5.293	3.817
3/30/23	06673	WBNU	F	20.6	64	4000	52.5	26	26.5	0.678815	4.924	3.817

Development • Supplementary information

**Date**	Date
**ID**	Bird ID Number
**Species**	Species (BCCH, TUTI, WBNU)
**Sex**	Male, Female, Unknown
**Weight**	Weight at capture (g)
**Noise_Level**	Noise Level (0, 44, 54, 64 dB SPL)
**Frequency**	Tone Frequency (1, 2, 2.5, 3.15, 4 kHz)
**Threshold**	Threshold (dB SPL)
**Spectrum_Level**	Noise Spectrum Level (6, 16, 26 db/Hz)
**Critical_Ratio**	Difference between Threshold and Spectrum_Level
**Amplitude**	Amplitude (voltage difference in µv)
**Latency_Max**	Time to Positive Peak since onset of sound at the ear (ms)
**Latency_Min**	Time to Negative Peak since onset of sound at the ear (ms)

Table S2. Data for calculating latency minimums and maximums for suprathreshold responses. Each stimulus was repeated twice to obtain two replicates, which were then averaged to determine responses.

ID	Date	Species	Frequency	Noise	Rep	Intensity	Latency_Max	Latency_Min	Max	Min	Amp	Latency_Max2	Latency_Min2	InterLatency
bcch_02594	22323	bcch	4000	cr00	a_	60	2.9345	3.6725	1.97E-06	-2.28E-06	4.24E-06	3.7145	4.4525	0.738
bcch_02594	22323	bcch	4000	cr00	b_	60	3.0165	3.7955	2.01E-06	-1.48E-06	3.49E-06	3.7965	4.5755	0.779
bcch_02594	22323	bcch	2500	cr00	a_	60	3.0165	3.8775	4.21E-06	-5.54E-06	9.75E-06	3.7965	4.6575	0.861
bcch_02594	22323	bcch	2500	cr00	b_	60	3.0575	3.8365	3.64E-06	-5.95E-06	9.59E-06	3.8375	4.6165	0.779
bcch_02594	22323	bcch	2000	cr00	a_	60	2.9755	3.8775	2.44E-06	-4.43E-06	6.86E-06	3.7555	4.6575	0.902
bcch_02594	22323	bcch	2000	cr00	b_	60	2.8525	3.8775	3.59E-06	-3.56E-06	7.16E-06	3.6325	4.6575	1.025
bcch_02594	22323	bcch	3150	cr00	a_	60	2.9345	3.7955	2.68E-06	-4.26E-06	6.94E-06	3.7145	4.5755	0.861
bcch_02594	22323	bcch	3150	cr00	b_	60	3.0575	3.7955	4.21E-06	-2.71E-06	6.92E-06	3.8375	4.5755	0.738
bcch_02594	22323	bcch	1000	cr00	a_	60	3.0985	3.6725	1.15E-06	-2.31E-06	3.46E-06	3.8785	4.4525	0.574
bcch_02594	22323	bcch	1000	cr00	b_	60	3.3445	3.8365	6.06E-07	-2.14E-06	2.75E-06	4.1245	4.6165	0.492
bcch_02594	22323	bcch	4000	cr44	a_	60	3.5905	4.1235	2.24E-06	-7.56E-07	2.99E-06	4.3705	4.9035	0.533
bcch_02594	22323	bcch	4000	cr44	b_	60	3.3445	4.1235	2.48E-06	-5.38E-07	3.02E-06	4.1245	4.9035	0.779
bcch_02594	22323	bcch	2500	cr44	a_	60	3.5495	4.5335	3.25E-06	-4.46E-06	7.71E-06	4.3295	5.3135	0.984
bcch_02594	22323	bcch	2500	cr44	b_	60	3.5495	4.4515	3.25E-06	-3.25E-06	6.50E-06	4.3295	5.2315	0.902
bcch_02594	22323	bcch	1000	cr44	a_	60	3.8365	4.9845	8.15E-07	-3.23E-06	4.05E-06	4.6165	5.7645	1.148
bcch_02594	22323	bcch	1000	cr44	b_	60	3.9185	5.0665	1.62E-06	-4.20E-06	5.82E-06	4.6985	5.8465	1.148
bcch_02594	22323	bcch	2000	cr44	a_	60	3.5905	4.5745	2.40E-06	-4.82E-06	7.22E-06	4.3705	5.3545	0.984
bcch_02594	22323	bcch	2000	cr44	b_	60	3.6315	4.4925	2.11E-06	-4.26E-06	6.38E-06	4.4115	5.2725	0.861
bcch_02594	22323	bcch	3150	cr44	a_	60	3.5085	4.3285	4.23E-06	-2.33E-06	6.56E-06	4.2885	5.1085	0.82
bcch_02594	22323	bcch	3150	cr44	b_	60	3.3445	4.2465	3.18E-06	-3.19E-06	6.37E-06	4.1245	5.0265	0.902
bcch_02594	22323	bcch	4000	cr54	a_	60	2.8525	3.7955	2.05E-06	-2.02E-07	2.26E-06	3.6325	4.5755	0.943
bcch_02594	22323	bcch	4000	cr54	b_	60	2.9755	3.5905	2.12E-06	-6.78E-07	2.79E-06	3.7555	4.3705	0.615
bcch_02594	22323	bcch	2500	cr54	a_	60	2.8935	3.6315	1.86E-06	-2.28E-06	4.15E-06	3.6735	4.4115	0.738
bcch_02594	22323	bcch	2500	cr54	b_	60	3.0985	3.6315	2.60E-06	-6.53E-07	3.25E-06	3.8785	4.4115	0.533
bcch_02594	22323	bcch	1000	cr54	a_	60	3.1805	4.0005	1.11E-06	-9.26E-07	2.04E-06	3.9605	4.7805	0.82
bcch_02594	22323	bcch	1000	cr54	b_	60	3.3855	4.0415	3.13E-07	-1.08E-06	1.39E-06	4.1655	4.8215	0.656
bcch_02594	22323	bcch	2000	cr54	a_	60	3.0165	3.7955	9.58E-07	-2.29E-06	3.25E-06	3.7965	4.5755	0.779
bcch_02594	22323	bcch	2000	cr54	b_	60	2.9755	3.7955	1.46E-06	-1.68E-06	3.14E-06	3.7555	4.5755	0.82
bcch_02594	22323	bcch	3150	cr54	a_	60	3.1395	3.7135	1.91E-06	-9.73E-07	2.88E-06	3.9195	4.4935	0.574
bcch_02594	22323	bcch	3150	cr54	b_	60	3.2625	3.7545	3.17E-06	4.71E-07	2.70E-06	4.0425	4.5345	0.492
bcch_02594	22323	bcch	4000	cr64	a_	60	3.3445	3.8775	2.33E-06	9.58E-07	1.37E-06	4.1245	4.6575	0.533
bcch_02594	22323	bcch	4000	cr64	b_	60	2.9345	4.0005	5.16E-07	-1.14E-06	1.66E-06	3.7145	4.7805	1.066
bcch_02594	22323	bcch	2500	cr64	a_	60	3.1805	3.8365	1.64E-06	-4.59E-07	2.09E-06	3.9605	4.6165	0.656
bcch_02594	22323	bcch	2500	cr64	b_	60	3.2625	4.0005	2.68E-06	-8.18E-07	3.49E-06	4.0425	4.7805	0.738
bcch_02594	22323	bcch	1000	cr64	a_	60	3.4675	4.3285	3.36E-07	-1.33E-06	1.66E-06	4.2475	5.1085	0.861
bcch_02594	22323	bcch	1000	cr64	b_	60	2.8525	3.2625	1.88E-07	-7.87E-07	9.75E-07	3.6325	4.0425	0.41
bcch_02594	22323	bcch	2000	cr64	a_	60	3.0985	4.0415	1.64E-06	-1.82E-06	3.46E-06	3.8785	4.8215	0.943
bcch_02594	22323	bcch	2000	cr64	b_	60	2.9345	4.0825	7.88E-07	-1.75E-06	2.54E-06	3.7145	4.8625	1.148
bcch_02594	22323	bcch	3150	cr64	a_	60	3.1805	4.0005	1.28E-06	-1.51E-06	2.79E-06	3.9605	4.7805	0.82
bcch_02594	22323	bcch	3150	cr64	b_	60	3.2625	3.9595	2.11E-06	-5.12E-07	2.62E-06	4.0425	4.7395	0.697
bcch_02595	22323	bcch	4000	cr00	a_	60	3.2215	3.8775	4.51E-06	8.90E-07	3.62E-06	4.0015	4.6575	0.656
bcch_02595	22323	bcch	4000	cr00	b_	60	3.2215	3.7545	1.34E-06	-1.25E-06	2.59E-06	4.0015	4.5345	0.533
bcch_02595	22323	bcch	2500	cr00	a_	60	3.3855	4.0005	4.93E-06	2.02E-07	4.73E-06	4.1655	4.7805	0.615
bcch_02595	22323	bcch	2500	cr00	b_	60	3.0985	3.9185	3.09E-06	-1.32E-06	4.40E-06	3.8785	4.6985	0.82
bcch_02595	22323	bcch	1000	cr00	a_	60	2.9345	4.2055	1.34E-06	-3.37E-06	4.70E-06	3.7145	4.9855	1.271
bcch_02595	22323	bcch	1000	cr00	b_	60	3.1395	4.4925	1.82E-06	-1.96E-06	3.78E-06	3.9195	5.2725	1.353
bcch_02595	22323	bcch	2000	cr00	a_	60	3.1395	4.2055	2.71E-06	-3.57E-06	6.28E-06	3.9195	4.9855	1.066
bcch_02595	22323	bcch	2000	cr00	b_	60	3.1395	3.9595	4.09E-06	-2.69E-06	6.78E-06	3.9195	4.7395	0.82
bcch_02595	22323	bcch	3150	cr00	a_	60	3.3855	4.0005	5.52E-06	-4.87E-07	6.01E-06	4.1655	4.7805	0.615
bcch_02595	22323	bcch	3150	cr00	b_	60	3.4265	4.0825	4.19E-06	2.24E-07	3.96E-06	4.2065	4.8625	0.656
bcch_02595	22323	bcch	4000	cr44	a_	60	3.1805	3.5905	3.25E-06	8.09E-07	2.44E-06	3.9605	4.3705	0.41
bcch_02595	22323	bcch	4000	cr44	b_	60	3.1395	3.6315	2.23E-06	1.25E-06	9.82E-07	3.9195	4.4115	0.492
bcch_02595	22323	bcch	2500	cr44	a_	60	3.0165	4.2875	4.13E-06	-2.20E-06	6.33E-06	3.7965	5.0675	1.271
bcch_02595	22323	bcch	2500	cr44	b_	60	3.2625	4.4105	4.11E-06	1.10E-07	4.00E-06	4.0425	5.1905	1.148
bcch_02595	22323	bcch	1000	cr44	a_	60	2.8115	4.4925	4.62E-07	-3.23E-06	3.69E-06	3.5915	5.2725	1.681
bcch_02595	22323	bcch	1000	cr44	b_	60	2.6885	4.7385	5.61E-07	-5.39E-06	5.95E-06	3.4685	5.5185	2.05
bcch_02595	22323	bcch	2000	cr44	a_	60	3.2215	4.3285	-1.66E-06	-4.05E-06	2.39E-06	4.0015	5.1085	1.107
bcch_02595	22323	bcch	2000	cr44	b_	60	2.9755	4.2875	3.82E-06	-2.68E-06	6.50E-06	3.7555	5.0675	1.312

bcch_02595	22323	bcch	3150	cr44	a_	60	3.0165	4.2875	3.02E-06	-6.38E-07	3.66E-06	3.7965	5.0675	1.271
bcch_02595	22323	bcch	3150	cr44	b_	60	3.1395	4.6155	3.90E-06	-1.62E-06	5.52E-06	3.9195	5.3955	1.476
bcch_02595	22323	bcch	4000	cr54	a_	60	4.8615	6.2965	2.42E-06	-2.47E-06	4.89E-06	5.6415	7.0765	1.435
bcch_02595	22323	bcch	4000	cr54	b_	60	5.1075	6.4605	2.43E-06	-5.12E-07	2.94E-06	5.8875	7.2405	1.353
bcch_02595	22323	bcch	2500	cr54	a_	60	3.1395	4.3285	2.23E-06	-1.51E-06	3.74E-06	3.9195	5.1085	1.189
bcch_02595	22323	bcch	2500	cr54	b_	60	2.9755	4.3285	1.43E-06	-1.35E-06	2.78E-06	3.7555	5.1085	1.353
bcch_02595	22323	bcch	2000	cr54	a_	60	2.8935	3.7545	8.59E-07	-2.25E-06	3.11E-06	3.6735	4.5345	0.861
bcch_02595	22323	bcch	2000	cr54	b_	60	3.2215	4.5335	2.79E-06	-1.51E-06	4.29E-06	4.0015	5.3135	1.312
bcch_02595	22323	bcch	3150	cr54	a_	60	3.0165	3.8365	1.84E-06	-1.37E-06	3.22E-06	3.7965	4.6165	0.82
bcch_02595	22323	bcch	3150	cr54	b_	60	3.2215	4.3285	3.55E-06	-2.30E-07	3.78E-06	4.0015	5.1085	1.107
bcch_02595	22323	bcch	4000	cr64	a_	60	5.5585	6.5425	1.03E-06	-1.18E-06	2.22E-06	6.3385	7.3225	0.984
bcch_02595	22323	bcch	4000	cr64	b_	60	5.3945	4.8205	1.91E-06	6.34E-08	1.85E-06	6.1745	5.6005	-0.574
bcch_02595	22323	bcch	1000	cr64	a_	60	3.0575	4.5335	1.01E-06	-3.77E-06	4.78E-06	3.8375	5.3135	1.476
bcch_02595	22323	bcch	1000	cr64	b_	60	3.3445	4.3695	1.13E-06	-5.88E-07	1.72E-06	4.1245	5.1495	1.025
bcch_02595	22323	bcch	2000	cr64	a_	60	3.2625	3.7955	1.23E-06	-7.14E-07	1.94E-06	4.0425	4.5755	0.533
bcch_02595	22323	bcch	2000	cr64	b_	60	2.8115	3.7955	2.65E-07	-2.60E-06	2.87E-06	3.5915	4.5755	0.984
bcch_02595	22323	bcch	3150	cr64	a_	60	2.9755	3.7955	1.17E-06	-1.52E-06	2.68E-06	3.7555	4.5755	0.82
bcch_02595	22323	bcch	3150	cr64	b_	60	3.1805	2.6475	2.60E-06	-1.29E-07	2.73E-06	3.9605	3.4275	-0.533
bcch_02596	22823	bcch	4000	cr00	a_	60	3.0165	4.4515	2.54E-06	-1.59E-06	4.13E-06	3.7965	5.2315	1.435
bcch_02596	22823	bcch	4000	cr00	b_	60	3.1395	4.4515	2.76E-06	-1.28E-06	4.04E-06	3.9195	5.2315	1.312
bcch_02596	22823	bcch	2500	cr00	a_	60	3.0985	4.0415	6.15E-06	-7.91E-06	1.41E-05	3.8785	4.8215	0.943
bcch_02596	22823	bcch	2500	cr00	b_	60	3.1805	4.0005	7.31E-06	-5.55E-06	1.29E-05	3.9605	4.7805	0.82
bcch_02596	22823	bcch	1000	cr00	a_	60	2.8935	4.0825	2.81E-06	-4.40E-06	7.20E-06	3.6735	4.8625	1.189
bcch_02596	22823	bcch	1000	cr00	b_	60	3.5085	4.3695	1.51E-06	-1.88E-06	3.39E-06	4.2885	5.1495	0.861
bcch_02596	22823	bcch	2000	cr00	a_	60	3.5085	4.0415	4.18E-06	-5.58E-06	9.76E-06	4.2885	4.8215	0.533
bcch_02596	22823	bcch	2000	cr00	b_	60	3.0575	4.0825	2.04E-06	-6.72E-06	8.76E-06	3.8375	4.8625	1.025
bcch_02596	22823	bcch	3150	cr00	a_	60	2.9345	4.0825	9.04E-06	-5.59E-06	1.46E-05	3.7145	4.8625	1.148
bcch_02596	22823	bcch	3150	cr00	b_	60	3.5085	4.7385	6.17E-06	-5.88E-06	1.20E-05	4.2885	5.5185	1.23
bcch_02596	22823	bcch	4000	cr44	a_	60	2.9755	3.5495	1.45E-06	-3.08E-07	1.76E-06	3.7555	4.3295	0.574
bcch_02596	22823	bcch	4000	cr44	b_	60	2.4015	3.7135	1.49E-06	-5.75E-07	2.07E-06	3.1815	4.4935	1.312
bcch_02596	22823	bcch	2500	cr44	a_	60	3.0985	3.7135	2.78E-06	-2.56E-06	5.34E-06	3.8785	4.4935	0.615
bcch_02596	22823	bcch	2500	cr44	b_	60	2.9755	3.7955	3.02E-06	-3.26E-06	6.28E-06	3.7555	4.5755	0.82
bcch_02596	22823	bcch	1000	cr44	a_	60	3.0575	4.6155	7.24E-07	-3.43E-06	4.15E-06	3.8375	5.3955	1.558
bcch_02596	22823	bcch	1000	cr44	b_	60	2.9345	4.5745	7.45E-07	-3.42E-06	4.16E-06	3.7145	5.3545	1.64
bcch_02596	22823	bcch	2000	cr44	a_	60	3.0165	4.3695	2.96E-06	-2.87E-06	5.83E-06	3.7965	5.1495	1.353
bcch_02596	22823	bcch	2000	cr44	b_	60	2.8525	3.7545	2.63E-06	-2.28E-06	4.90E-06	3.6325	4.5345	0.902
bcch_02596	22823	bcch	3150	cr44	a_	60	3.1395	3.8365	2.72E-06	-1.79E-06	4.51E-06	3.9195	4.6165	0.697
bcch_02596	22823	bcch	3150	cr44	b_	60	3.0575	3.8365	2.66E-06	-9.68E-07	3.62E-06	3.8375	4.6165	0.779
bcch_02596	22823	bcch	4000	cr54	a_	60	5.3535	6.0095	2.78E-06	-5.09E-07	3.29E-06	6.1335	6.7895	0.656
bcch_02596	22823	bcch	4000	cr54	b_	60	5.3535	7.2395	2.90E-06	-2.50E-06	5.40E-06	6.1335	8.0195	1.886
bcch_02596	22823	bcch	2500	cr54	a_	60	3.1395	3.7955	1.35E-06	-2.20E-06	3.55E-06	3.9195	4.5755	0.656
bcch_02596	22823	bcch	2500	cr54	b_	60	3.3035	3.8365	1.45E-06	-1.25E-06	2.71E-06	4.0835	4.6165	0.533
bcch_02596	22823	bcch	1000	cr54	a_	60	5.8455	5.1485	2.75E-06	-1.10E-06	3.85E-06	6.6255	5.9285	-0.697
bcch_02596	22823	bcch	1000	cr54	b_	60	5.9685	6.9935	2.35E-06	-2.01E-07	2.55E-06	6.7485	7.7735	1.025
bcch_02596	22823	bcch	2000	cr54	a_	60	2.9755	4.4515	1.66E-06	-2.20E-06	3.87E-06	3.7555	5.2315	1.476
bcch_02596	22823	bcch	2000	cr54	b_	60	3.2625	4.6155	1.31E-06	-2.05E-06	3.36E-06	4.0425	5.3955	1.353
bcch_02596	22823	bcch	3150	cr54	a_	60	3.3035	3.8365	1.58E-06	-1.14E-06	2.72E-06	4.0835	4.6165	0.533
bcch_02596	22823	bcch	3150	cr54	b_	60	2.8115	4.0005	1.77E-06	-1.87E-06	3.64E-06	3.5915	4.7805	1.189
bcch_02596	22823	bcch	4000	cr64	a_	60	2.0325	4.1235	2.54E-06	-2.72E-06	5.26E-06	2.8125	4.9035	2.091
bcch_02596	22823	bcch	4000	cr64	b_	60	5.4355	6.7885	1.61E-06	-1.57E-06	3.19E-06	6.2155	7.5685	1.353
bcch_02596	22823	bcch	2500	cr64	a_	60	1.0485	2.6475	9.81E-07	-1.50E-06	2.48E-06	1.8285	3.4275	1.599
bcch_02596	22823	bcch	2500	cr64	b_	60	2.8525	3.8365	7.46E-08	-3.34E-06	3.41E-06	3.6325	4.6165	0.984
bcch_02596	22823	bcch	1000	cr64	a_	60	3.1395	4.7795	1.42E-06	-6.37E-07	2.06E-06	3.9195	5.5595	1.64
bcch_02596	22823	bcch	1000	cr64	b_	60	3.5495	4.5745	-1.10E-07	-1.71E-06	1.60E-06	4.3295	5.3545	1.025
bcch_02596	22823	bcch	2000	cr64	a_	60	2.5655	4.9435	9.67E-07	-2.19E-06	3.16E-06	3.3455	5.7235	2.378
bcch_02596	22823	bcch	2000	cr64	b_	60	3.2215	4.6155	4.53E-06	-9.86E-07	5.51E-06	4.0015	5.3955	1.394
bcch_02596	22823	bcch	3150	cr64	a_	60	2.5655	3.7135	8.19E-07	-2.68E-06	3.50E-06	3.3455	4.4935	1.148
bcch_02596	22823	bcch	3150	cr64	b_	60	2.5245	3.7545	8.79E-07	-1.57E-06	2.45E-06	3.3045	4.5345	1.23
bcch_02597	22823	bcch	4000	cr00	a_	60	2.5655	3.7955	2.30E-06	-1.90E-07	2.49E-06	3.3455	4.5755	1.23

bcch_02597	22823	bcch	4000	cr00	b_	60	3.0985	4.0415	2.05E-06	-6.79E-07	2.73E-06	3.8785	4.8215	0.943
bcch_02597	22823	bcch	2500	cr00	a_	60	2.9755	3.7135	4.14E-06	-3.92E-06	8.05E-06	3.7555	4.4935	0.738
bcch_02597	22823	bcch	2500	cr00	b_	60	2.8115	3.7955	4.02E-06	-4.95E-06	8.97E-06	3.5915	4.5755	0.984
bcch_02597	22823	bcch	1000	cr00	a_	60	2.9755	3.7135	1.17E-06	-3.22E-06	4.39E-06	3.7555	4.4935	0.738
bcch_02597	22823	bcch	1000	cr00	b_	60	3.3035	3.8365	7.53E-07	-2.48E-06	3.23E-06	4.0835	4.6165	0.533
bcch_02597	22823	bcch	2000	cr00	a_	60	3.0165	3.7135	3.60E-06	-3.75E-06	7.35E-06	3.7965	4.4935	0.697
bcch_02597	22823	bcch	2000	cr00	b_	60	2.8525	3.7545	3.99E-06	-4.56E-06	8.55E-06	3.6325	4.5345	0.902
bcch_02597	22823	bcch	3150	cr00	a_	60	2.9345	3.6725	3.43E-06	-2.56E-06	5.99E-06	3.7145	4.4525	0.738
bcch_02597	22823	bcch	3150	cr00	b_	60	2.8935	3.7545	3.65E-06	-2.68E-06	6.33E-06	3.6735	4.5345	0.861
bcch_02597	22823	bcch	4000	cr44	a_	60	2.6885	2.0735	2.09E-06	2.18E-07	1.87E-06	3.4685	2.8535	-0.615
bcch_02597	22823	bcch	4000	cr44	b_	60	2.8525	4.2055	2.51E-06	5.05E-07	2.00E-06	3.6325	4.9855	1.353
bcch_02597	22823	bcch	2500	cr44	a_	60	2.8935	3.6315	2.27E-06	-2.12E-06	4.39E-06	3.6735	4.4115	0.738
bcch_02597	22823	bcch	2500	cr44	b_	60	2.9345	3.6725	3.13E-06	-1.35E-06	4.48E-06	3.7145	4.4525	0.738
bcch_02597	22823	bcch	1000	cr44	a_	60	2.7705	4.1645	1.45E-06	-2.79E-06	4.25E-06	3.5505	4.9445	1.394
bcch_02597	22823	bcch	1000	cr44	b_	60	2.6475	3.7545	1.38E-06	-1.66E-06	3.04E-06	3.4275	4.5345	1.107
bcch_02597	22823	bcch	2000	cr44	a_	60	2.6475	3.8775	2.53E-06	-3.00E-06	5.52E-06	3.4275	4.6575	1.23
bcch_02597	22823	bcch	2000	cr44	b_	60	2.7295	3.9185	2.44E-06	-2.96E-06	5.40E-06	3.5095	4.6985	1.189
bcch_02597	22823	bcch	3150	cr44	a_	60	2.6065	3.6725	3.04E-06	-8.94E-07	3.94E-06	3.3865	4.4525	1.066
bcch_02597	22823	bcch	3150	cr44	b_	60	2.6065	3.6725	2.88E-06	-4.20E-07	3.30E-06	3.3865	4.4525	1.066
bcch_02597	22823	bcch	4000	cr54	a_	60	3.1805	3.8775	1.91E-06	4.61E-07	1.45E-06	3.9605	4.6575	0.697
bcch_02597	22823	bcch	4000	cr54	b_	60	3.1805	4.3285	1.98E-06	4.54E-07	1.52E-06	3.9605	5.1085	1.148
bcch_02597	22823	bcch	2500	cr54	a_	60	2.7295	3.8775	1.77E-06	-2.15E-06	3.92E-06	3.5095	4.6575	1.148
bcch_02597	22823	bcch	2500	cr54	b_	60	2.9345	3.8365	1.91E-06	-1.00E-06	2.91E-06	3.7145	4.6165	0.902
bcch_02597	22823	bcch	1000	cr54	a_	60	3.5495	4.3285	5.41E-08	-1.90E-06	1.95E-06	4.3295	5.1085	0.779
bcch_02597	22823	bcch	1000	cr54	b_	60	2.9755	4.1645	7.59E-07	-2.75E-06	3.51E-06	3.7555	4.9445	1.189
bcch_02597	22823	bcch	2000	cr54	a_	60	2.9755	3.7955	1.65E-06	-2.11E-06	3.76E-06	3.7555	4.5755	0.82
bcch_02597	22823	bcch	2000	cr54	b_	60	2.9345	3.7955	1.62E-06	-2.33E-06	3.95E-06	3.7145	4.5755	0.861
bcch_02597	22823	bcch	3150	cr54	a_	60	2.9345	4.0415	2.08E-06	-5.91E-07	2.67E-06	3.7145	4.8215	1.107
bcch_02597	22823	bcch	3150	cr54	b_	60	2.6475	3.9595	1.88E-06	-7.92E-07	2.68E-06	3.4275	4.7395	1.312
bcch_02597	22823	bcch	4000	cr64	a_	60	4.8615	6.0095	1.27E-06	-8.14E-07	2.08E-06	5.6415	6.7895	1.148
bcch_02597	22823	bcch	4000	cr64	b_	60	3.0985	3.7545	1.38E-06	8.79E-08	1.29E-06	3.8785	4.5345	0.656
bcch_02597	22823	bcch	2500	cr64	a_	60	3.1805	3.9595	1.26E-06	-1.28E-06	2.54E-06	3.9605	4.7395	0.779
bcch_02597	22823	bcch	2500	cr64	b_	60	3.2215	4.1235	1.66E-06	-1.20E-06	2.86E-06	4.0015	4.9035	0.902
bcch_02597	22823	bcch	1000	cr64	a_	60	3.2625	4.6155	3.61E-07	-1.40E-06	1.76E-06	4.0425	5.3955	1.353
bcch_02597	22823	bcch	1000	cr64	b_	60	3.0575	4.5745	6.60E-07	-2.40E-06	3.06E-06	3.8375	5.3545	1.517
bcch_02597	22823	bcch	2000	cr64	a_	60	3.3855	4.0005	1.50E-06	-1.31E-06	2.81E-06	4.1655	4.7805	0.615
bcch_02597	22823	bcch	2000	cr64	b_	60	2.9755	3.9595	1.30E-06	-1.46E-06	2.76E-06	3.7555	4.7395	0.984
bcch_02597	22823	bcch	3150	cr64	a_	60	3.0575	4.2055	1.97E-06	-1.53E-06	3.50E-06	3.8375	4.9855	1.148
bcch_02597	22823	bcch	3150	cr64	b_	60	3.2215	4.5745	8.41E-07	-6.68E-07	1.51E-06	4.0015	5.3545	1.353
bcch_02598	30223	bcch	4000	cr00	a_	60	3.5085	4.2055	2.42E-06	-9.53E-07	3.37E-06	4.2885	4.9855	0.697
bcch_02598	30223	bcch	4000	cr00	b_	60	3.3855	4.0825	2.16E-06	-6.11E-07	2.77E-06	4.1655	4.8625	0.697
bcch_02598	30223	bcch	2500	cr00	a_	60	3.5905	4.3695	1.65E-06	-1.99E-06	3.63E-06	4.3705	5.1495	0.779
bcch_02598	30223	bcch	2500	cr00	b_	60	3.7135	4.3285	1.67E-06	-2.19E-06	3.86E-06	4.4935	5.1085	0.615
bcch_02598	30223	bcch	1000	cr00	a_	60	3.3035	4.7385	4.79E-07	-2.83E-06	3.31E-06	4.0835	5.5185	1.435
bcch_02598	30223	bcch	1000	cr00	b_	60	3.7135	4.7795	6.24E-07	-2.53E-06	3.15E-06	4.4935	5.5595	1.066
bcch_02598	30223	bcch	2000	cr00	a_	60	3.7135	4.4925	1.28E-06	-2.48E-06	3.75E-06	4.4935	5.2725	0.779
bcch_02598	30223	bcch	2000	cr00	b_	60	3.4265	4.6565	9.60E-07	-2.93E-06	3.89E-06	4.2065	5.4365	1.23
bcch_02598	30223	bcch	3150	cr00	a_	60	3.5495	4.4105	2.69E-06	-2.61E-06	5.30E-06	4.3295	5.1905	0.861
bcch_02598	30223	bcch	3150	cr00	b_	60	3.5085	4.3285	3.07E-06	-1.62E-06	4.70E-06	4.2885	5.1085	0.82
bcch_02598	30223	bcch	4000	cr44	a_	60	3.3445	4.0005	2.34E-06	-6.24E-07	2.96E-06	4.1245	4.7805	0.656
bcch_02598	30223	bcch	4000	cr44	b_	60	3.2625	3.9595	2.73E-06	-5.75E-07	3.30E-06	4.0425	4.7395	0.697
bcch_02598	30223	bcch	2500	cr44	a_	60	3.4675	4.1235	2.70E-06	-2.46E-06	5.15E-06	4.2475	4.9035	0.656
bcch_02598	30223	bcch	2500	cr44	b_	60	3.4265	4.1645	2.04E-06	-2.29E-06	4.33E-06	4.2065	4.9445	0.738
bcch_02598	30223	bcch	1000	cr44	a_	60	3.8365	4.6975	4.26E-07	-2.15E-06	2.58E-06	4.6165	5.4775	0.861
bcch_02598	30223	bcch	1000	cr44	b_	60	3.7135	4.7795	6.24E-07	-2.53E-06	3.15E-06	4.4935	5.5595	1.066
bcch_02598	30223	bcch	2000	cr44	a_	60	3.5495	4.3285	1.12E-06	-3.31E-06	4.44E-06	4.3295	5.1085	0.779
bcch_02598	30223	bcch	2000	cr44	b_	60	3.3855	4.4105	9.13E-07	-3.74E-06	4.65E-06	4.1655	5.1905	1.025
bcch_02598	30223	bcch	3150	cr44	a_	60	3.2215	4.2055	2.65E-06	-2.30E-06	4.95E-06	4.0015	4.9855	0.984
bcch_02598	30223	bcch	3150	cr44	b_	60	3.3855	4.0825	2.55E-06	-1.89E-06	4.44E-06	4.1655	4.8625	0.697

bcch_02598	30223	bcch	4000	cr54	a_	60	3.4265	3.7955	1.60E-06	-3.31E-07	1.93E-06	4.2065	4.5755	0.369
bcch_02598	30223	bcch	4000	cr54	b_	60	3.1395	3.9185	1.58E-06	-1.24E-06	2.82E-06	3.9195	4.6985	0.779
bcch_02598	30223	bcch	2500	cr54	a_	60	3.5905	4.0825	1.27E-06	-7.50E-07	2.02E-06	4.3705	4.8625	0.492
bcch_02598	30223	bcch	2500	cr54	b_	60	3.5085	4.1645	1.24E-06	-2.21E-06	3.45E-06	4.2885	4.9445	0.656
bcch_02598	30223	bcch	1000	cr54	a_	60	3.1395	4.6565	7.49E-07	-2.44E-06	3.19E-06	3.9195	5.4365	1.517
bcch_02598	30223	bcch	1000	cr54	b_	60	3.0575	4.7795	-7.17E-08	-2.26E-06	2.18E-06	3.8375	5.5595	1.722
bcch_02598	30223	bcch	2000	cr54	a_	60	3.4675	4.2055	3.34E-07	-1.28E-06	1.61E-06	4.2475	4.9855	0.738
bcch_02598	30223	bcch	2000	cr54	b_	60	3.4675	4.0825	1.87E-06	-1.45E-06	3.32E-06	4.2475	4.8625	0.615
bcch_02598	30223	bcch	3150	cr54	a_	60	3.2215	4.0415	1.50E-06	-1.69E-06	3.18E-06	4.0015	4.8215	0.82
bcch_02598	30223	bcch	3150	cr54	b_	60	3.2215	4.2465	1.13E-06	-2.47E-06	3.59E-06	4.0015	5.0265	1.025
bcch_02598	30223	bcch	4000	cr64	a_	60	3.4265	4.4105	1.41E-06	-2.48E-07	1.66E-06	4.2065	5.1905	0.984
bcch_02598	30223	bcch	4000	cr64	b_	60	3.2625	4.1235	5.63E-07	-1.48E-06	2.05E-06	4.0425	4.9035	0.861
bcch_02598	30223	bcch	2500	cr64	a_	60	3.5495	4.3695	1.86E-07	-2.04E-06	2.23E-06	4.3295	5.1495	0.82
bcch_02598	30223	bcch	2500	cr64	b_	60	3.7135	4.2465	1.09E-06	-7.09E-09	1.09E-06	4.4935	5.0265	0.533
bcch_02598	30223	bcch	1000	cr64	a_	60	5.8455	6.5425	2.34E-07	-1.17E-06	1.40E-06	6.6255	7.3225	0.697
bcch_02598	30223	bcch	1000	cr64	b_	60	4.3285	5.8865	7.00E-07	-1.57E-06	2.27E-06	5.1085	6.6665	1.558
bcch_02598	30223	bcch	2000	cr64	a_	60	3.5905	4.5745	5.46E-07	-1.21E-06	1.76E-06	4.3705	5.3545	0.984
bcch_02598	30223	bcch	2000	cr64	b_	60	5.6815	5.0255	8.81E-07	-1.45E-07	1.03E-06	6.4615	5.8055	-0.656
bcch_02598	30223	bcch	3150	cr64	a_	60	3.5495	4.0825	1.28E-06	-4.72E-07	1.75E-06	4.3295	4.8625	0.533
bcch_02598	30223	bcch	3150	cr64	b_	60	3.7135	4.1235	2.26E-06	7.43E-07	1.51E-06	4.4935	4.9035	0.41
bcch_02599	32123	bcch	4000	cr00	a_	60	3.8365	4.2875	6.72E-07	-3.37E-07	1.01E-06	4.6165	5.0675	0.451
bcch_02599	32123	bcch	4000	cr00	b_	60	3.3035	4.3285	1.36E-06	-8.26E-07	2.19E-06	4.0835	5.1085	1.025
bcch_02599	32123	bcch	2500	cr00	a_	60	3.6725	4.5745	1.50E-06	-1.38E-06	2.89E-06	4.4525	5.3545	0.902
bcch_02599	32123	bcch	2500	cr00	b_	60	3.4675	4.5745	1.73E-06	-1.58E-06	3.31E-06	4.2475	5.3545	1.107
bcch_02599	32123	bcch	1000	cr00	a_	60	4.0825	4.9025	6.20E-07	-7.77E-07	1.40E-06	4.8625	5.6825	0.82
bcch_02599	32123	bcch	1000	cr00	b_	60	4.1645	5.1075	7.80E-07	-1.38E-06	2.16E-06	4.9445	5.8875	0.943
bcch_02599	32123	bcch	2000	cr00	a_	60	3.8775	4.7385	1.45E-06	-1.55E-06	3.00E-06	4.6575	5.5185	0.861
bcch_02599	32123	bcch	2000	cr00	b_	60	3.5495	4.4515	1.59E-06	-2.33E-06	3.91E-06	4.3295	5.2315	0.902
bcch_02599	32123	bcch	3150	cr00	a_	60	3.6315	4.3695	1.63E-06	-1.89E-06	3.52E-06	4.4115	5.1495	0.738
bcch_02599	32123	bcch	3150	cr00	b_	60	3.4265	4.2055	2.02E-06	-2.62E-06	4.64E-06	4.2065	4.9855	0.779
bcch_02599	32123	bcch	4000	cr44	a_	60	4.3285	3.9595	1.53E-06	1.23E-07	1.40E-06	5.1085	4.7395	-0.369
bcch_02599	32123	bcch	4000	cr44	b_	60	3.1395	4.1645	1.94E-06	-3.58E-07	2.29E-06	3.9195	4.9445	1.025
bcch_02599	32123	bcch	2500	cr44	a_	60	3.0985	4.0005	1.51E-06	-2.77E-06	4.28E-06	3.8785	4.7805	0.902
bcch_02599	32123	bcch	2500	cr44	b_	60	3.0985	4.0005	9.77E-07	-1.82E-06	2.79E-06	3.8785	4.7805	0.902
bcch_02599	32123	bcch	1000	cr44	a_	60	3.0985	4.8615	1.95E-06	-3.45E-06	5.40E-06	3.8785	5.6415	1.763
bcch_02599	32123	bcch	1000	cr44	b_	60	3.3445	4.8615	1.56E-06	-2.52E-06	4.07E-06	4.1245	5.6415	1.517
bcch_02599	32123	bcch	2000	cr44	a_	60	2.8525	4.0415	1.74E-06	-3.02E-06	4.76E-06	3.6325	4.8215	1.189
bcch_02599	32123	bcch	2000	cr44	b_	60	3.1805	4.0415	2.31E-06	-2.12E-06	4.43E-06	3.9605	4.8215	0.861
bcch_02599	32123	bcch	3150	cr44	a_	60	3.2625	4.0825	2.16E-06	-2.27E-07	2.39E-06	4.0425	4.8625	0.82
bcch_02599	32123	bcch	3150	cr44	b_	60	2.9345	4.2875	2.24E-06	-1.17E-06	3.41E-06	3.7145	5.0675	1.353
bcch_02599	32123	bcch	4000	cr54	a_	60	3.5905	3.9595	1.09E-06	1.82E-07	9.05E-07	4.3705	4.7395	0.369
bcch_02599	32123	bcch	4000	cr54	b_	60	4.5745	4.1645	9.68E-07	-4.08E-07	1.38E-06	5.3545	4.9445	-0.41
bcch_02599	32123	bcch	2500	cr54	a_	60	3.0985	3.9595	1.55E-06	-1.68E-06	3.23E-06	3.8785	4.7395	0.861
bcch_02599	32123	bcch	2500	cr54	b_	60	2.8525	4.0005	1.43E-06	-1.12E-06	2.55E-06	3.6325	4.7805	1.148
bcch_02599	32123	bcch	1000	cr54	a_	60	3.1395	4.2055	1.69E-06	-1.20E-06	2.89E-06	3.9195	4.9855	1.066
bcch_02599	32123	bcch	1000	cr54	b_	60	3.6315	5.0665	1.35E-06	-1.21E-06	2.57E-06	4.4115	5.8465	1.435
bcch_02599	32123	bcch	2000	cr54	a_	60	3.0165	4.1235	9.73E-07	-9.68E-07	1.94E-06	3.7965	4.9035	1.107
bcch_02599	32123	bcch	2000	cr54	b_	60	3.1805	4.0825	7.24E-07	-1.40E-06	2.12E-06	3.9605	4.8625	0.902
bcch_02599	32123	bcch	3150	cr54	a_	60	3.2215	3.9185	1.59E-06	-1.31E-06	2.90E-06	4.0015	4.6985	0.697
bcch_02599	32123	bcch	3150	cr54	b_	60	3.0575	4.0415	1.33E-06	-1.40E-06	2.73E-06	3.8375	4.8215	0.984
bcch_02599	32123	bcch	4000	cr64	a_	60	3.9595	3.5495	1.21E-06	3.67E-07	8.42E-07	4.7395	4.3295	-0.41
bcch_02599	32123	bcch	4000	cr64	b_	60	3.4675	4.2875	1.10E-06	3.23E-09	1.10E-06	4.2475	5.0675	0.82
bcch_02599	32123	bcch	2500	cr64	a_	60	3.5495	4.3695	1.75E-06	-4.86E-07	2.23E-06	4.3295	5.1495	0.82
bcch_02599	32123	bcch	2500	cr64	b_	60	3.3035	4.3285	8.23E-07	-1.77E-06	2.60E-06	4.0835	5.1085	1.025
bcch_02599	32123	bcch	1000	cr64	a_	60	3.4265	4.8205	8.49E-07	-1.30E-06	2.15E-06	4.2065	5.6005	1.394
bcch_02599	32123	bcch	1000	cr64	b_	60	3.1395	3.9595	-1.75E-08	-6.44E-07	6.27E-07	3.9195	4.7395	0.82
bcch_02599	32123	bcch	2000	cr64	a_	60	3.7955	4.1235	5.28E-07	-1.59E-07	6.88E-07	4.5755	4.9035	0.328
bcch_02599	32123	bcch	2000	cr64	b_	60	2.4425	4.0415	1.03E-06	-5.71E-07	1.60E-06	3.2225	4.8215	1.599
bcch_02599	32123	bcch	3150	cr64	a_	60	2.3605	4.5335	9.98E-07	-1.46E-06	2.46E-06	3.1405	5.3135	2.173

bcch_02599	32123	bcch	3150	cr64	b_	60	3.3855	3.9595	8.08E-07	-6.08E-07	1.42E-06	4.1655	4.7395	0.574
tuti_06664	20723	tuti	4000	cr00	a_	60	3.3855	4.0825	8.22E-07	-1.82E-06	2.64E-06	4.1655	4.8625	0.697
tuti_06664	20723	tuti	4000	cr00	b_	60	3.1805	4.0415	6.04E-07	-1.79E-06	2.39E-06	3.9605	4.8215	0.861
tuti_06664	20723	tuti	2500	cr00	a_	60	3.5085	4.5335	1.36E-06	-5.03E-06	6.39E-06	4.2885	5.3135	1.025
tuti_06664	20723	tuti	2500	cr00	b_	60	3.4675	4.4925	1.26E-06	-4.83E-06	6.09E-06	4.2475	5.2725	1.025
tuti_06664	20723	tuti	1000	cr00	a_	60	3.9185	5.1075	-1.57E-07	-2.93E-06	2.77E-06	4.6985	5.8875	1.189
tuti_06664	20723	tuti	1000	cr00	b_	60	4.1645	5.1075	5.04E-07	-1.86E-06	2.36E-06	4.9445	5.8875	0.943
tuti_06664	20723	tuti	2000	cr00	a_	60	3.5085	4.6565	1.34E-06	-4.12E-06	5.46E-06	4.2885	5.4365	1.148
tuti_06664	20723	tuti	2000	cr00	b_	60	3.5495	4.6975	1.00E-06	-4.45E-06	5.45E-06	4.3295	5.4775	1.148
tuti_06664	20723	tuti	3150	cr00	a_	60	3.1395	4.2055	1.12E-06	-4.41E-06	5.53E-06	3.9195	4.9855	1.066
tuti_06664	20723	tuti	3150	cr00	b_	60	3.2215	4.2465	1.49E-06	-3.53E-06	5.02E-06	4.0015	5.0265	1.025
tuti_06664	20723	tuti	4000	cr44	a_	60	2.5245	3.3445	9.30E-07	-1.23E-06	2.16E-06	3.3045	4.1245	0.82
tuti_06664	20723	tuti	4000	cr44	b_	60	2.7295	3.5495	2.03E-06	-1.35E-06	3.37E-06	3.5095	4.3295	0.82
tuti_06664	20723	tuti	2500	cr44	a_	60	2.6475	3.7545	1.41E-06	-2.12E-06	3.54E-06	3.4275	4.5345	1.107
tuti_06664	20723	tuti	2500	cr44	b_	60	2.5655	3.8365	2.01E-06	-3.06E-06	5.07E-06	3.3455	4.6165	1.271
tuti_06664	20723	tuti	1000	cr44	a_	60	2.4425	3.8365	6.13E-07	-1.48E-06	2.09E-06	3.2225	4.6165	1.394
tuti_06664	20723	tuti	1000	cr44	b_	60	3.2215	3.7135	3.60E-07	-1.00E-06	1.36E-06	4.0015	4.4935	0.492
tuti_06664	20723	tuti	2000	cr44	a_	60	2.6475	3.9185	7.15E-07	-3.18E-06	3.89E-06	3.4275	4.6985	1.271
tuti_06664	20723	tuti	2000	cr44	b_	60	2.6885	4.0415	8.53E-07	-2.28E-06	3.13E-06	3.4685	4.8215	1.353
tuti_06664	20723	tuti	3150	cr44	a_	60	2.1965	3.5085	1.18E-06	-2.39E-06	3.57E-06	2.9765	4.2885	1.312
tuti_06664	20723	tuti	3150	cr44	b_	60	3.0575	3.5905	7.15E-07	-2.71E-06	3.42E-06	3.8375	4.3705	0.533
tuti_06664	20723	tuti	4000	cr54	a_	60	2.8935	3.5495	7.51E-07	-7.56E-07	1.51E-06	3.6735	4.3295	0.656
tuti_06664	20723	tuti	4000	cr54	b_	60	2.8935	3.5085	7.79E-07	-1.03E-06	1.81E-06	3.6735	4.2885	0.615
tuti_06664	20723	tuti	2500	cr54	a_	60	2.9755	4.0825	6.44E-07	-1.70E-06	2.34E-06	3.7555	4.8625	1.107
tuti_06664	20723	tuti	2500	cr54	b_	60	2.9755	4.1235	7.78E-07	-2.14E-06	2.92E-06	3.7555	4.9035	1.148
tuti_06664	20723	tuti	1000	cr54	a_	60	3.1395	4.2875	5.07E-07	-1.27E-06	1.78E-06	3.9195	5.0675	1.148
tuti_06664	20723	tuti	1000	cr54	b_	60	3.3855	4.4105	6.09E-07	-1.23E-06	1.84E-06	4.1655	5.1905	1.025
tuti_06664	20723	tuti	4000	cr64	a_	60	3.1805	3.6315	9.17E-07	-5.37E-07	1.45E-06	3.9605	4.4115	0.451
tuti_06664	20723	tuti	4000	cr64	b_	60	2.8935	3.5495	2.61E-07	-8.11E-07	1.07E-06	3.6735	4.3295	0.656
tuti_06664	20723	tuti	2500	cr64	a_	60	2.7295	3.8365	6.25E-07	-1.19E-06	1.81E-06	3.5095	4.6165	1.107
tuti_06664	20723	tuti	2500	cr64	b_	60	2.6885	3.7955	6.76E-07	-1.43E-06	2.10E-06	3.4685	4.5755	1.107
tuti_06664	20723	tuti	1000	cr64	a_	60	3.1395	3.7135	2.87E-07	-3.68E-07	6.54E-07	3.9195	4.4935	0.574
tuti_06664	20723	tuti	1000	cr64	b_	60	3.2625	4.1235	6.55E-08	-7.08E-07	7.74E-07	4.0425	4.9035	0.861
tuti_06664	20723	tuti	2000	cr64	a_	60	3.0575	3.8365	4.43E-07	-6.60E-07	1.10E-06	3.8375	4.6165	0.779
tuti_06664	20723	tuti	2000	cr64	b_	60	2.4425	4.4515	1.07E-06	-6.33E-07	1.70E-06	3.2225	5.2315	2.009
tuti_06664	20723	tuti	3150	cr64	a_	60	2.8935	3.7955	3.47E-07	-1.23E-06	1.58E-06	3.6735	4.5755	0.902
tuti_06664	20723	tuti	3150	cr64	b_	60	2.5655	4.0005	9.81E-07	-9.49E-07	1.93E-06	3.3455	4.7805	1.435
tuti_06665	20723	tuti	4000	cr00	a_	60	3.1805	3.8365	1.24E-06	-1.61E-06	2.85E-06	3.9605	4.6165	0.656
tuti_06665	20723	tuti	4000	cr00	b_	60	2.9345	3.7545	1.43E-06	-1.60E-06	3.02E-06	3.7145	4.5345	0.82
tuti_06665	20723	tuti	1000	cr00	a_	60	3.1395	4.3285	1.69E-06	-2.46E-06	4.15E-06	3.9195	5.1085	1.189
tuti_06665	20723	tuti	1000	cr00	b_	60	3.1805	4.2465	1.70E-06	-1.55E-06	3.26E-06	3.9605	5.0265	1.066
tuti_06665	20723	tuti	4000	cr44	a_	60	2.8935	3.5905	1.71E-06	-4.62E-07	2.18E-06	3.6735	4.3705	0.697
tuti_06665	20723	tuti	4000	cr44	b_	60	2.7295	3.4265	1.05E-06	-8.83E-07	1.93E-06	3.5095	4.2065	0.697
tuti_06665	20723	tuti	1000	cr44	a_	60	2.6885	4.2465	6.81E-07	-1.71E-06	2.40E-06	3.4685	5.0265	1.558
tuti_06665	20723	tuti	1000	cr44	b_	60	3.0575	4.2055	8.03E-07	-1.74E-06	2.55E-06	3.8375	4.9855	1.148
tuti_06665	20723	tuti	4000	cr54	a_	60	2.7705	3.4675	1.07E-06	3.70E-08	1.03E-06	3.5505	4.2475	0.697
tuti_06665	20723	tuti	4000	cr54	b_	60	2.8525	3.4675	1.24E-06	-3.66E-07	1.61E-06	3.6325	4.2475	0.615
tuti_06665	20723	tuti	4000	cr64	a_	60	2.8525	3.5495	1.04E-07	-7.09E-07	8.13E-07	3.6325	4.3295	0.697
tuti_06665	20723	tuti	4000	cr64	b_	60	3.0985	3.5085	9.17E-07	-2.10E-07	1.13E-06	3.8785	4.2885	0.41
tuti_06665	20723	tuti	2500	cr64	a_	60	2.7705	3.9185	5.88E-07	-1.29E-06	1.88E-06	3.5505	4.6985	1.148
tuti_06665	20723	tuti	2500	cr64	b_	60	2.9755	3.6725	8.11E-07	-1.83E-06	2.64E-06	3.7555	4.4525	0.697
tuti_06666	21423	tuti	4000	cr00	a_	60	2.5245	3.4675	1.27E-06	-3.50E-06	4.77E-06	3.3045	4.2475	0.943
tuti_06666	21423	tuti	4000	cr00	b_	60	2.4835	3.3035	1.33E-06	-3.70E-06	5.03E-06	3.2635	4.0835	0.82
tuti_06666	21423	tuti	2500	cr00	a_	60	2.9345	3.7545	3.07E-06	-6.30E-06	9.37E-06	3.7145	4.5345	0.82
tuti_06666	21423	tuti	2500	cr00	b_	60	2.7705	3.6725	2.65E-06	-6.24E-06	8.89E-06	3.5505	4.4525	0.902
tuti_06666	21423	tuti	1000	cr00	a_	60	2.8525	3.6725	1.94E-06	-9.59E-07	2.90E-06	3.6325	4.4525	0.82
tuti_06666	21423	tuti	1000	cr00	b_	60	2.9345	3.5905	1.60E-06	-1.05E-06	2.65E-06	3.7145	4.3705	0.656
tuti_06666	21423	tuti	2000	cr00	a_	60	2.8115	3.7545	2.26E-06	-3.75E-06	6.01E-06	3.5915	4.5345	0.943
tuti_06666	21423	tuti	2000	cr00	b_	60	2.8525	3.6315	2.72E-06	-3.82E-06	6.54E-06	3.6325	4.4115	0.779

tuti_06666	21423	tuti	3150	cr00	a_	60	2.6475	3.5905	1.16E-06	-5.38E-06	6.54E-06	3.4275	4.3705	0.943
tuti_06666	21423	tuti	3150	cr00	b_	60	2.7295	3.6315	1.80E-06	-6.52E-06	8.31E-06	3.5095	4.4115	0.902
tuti_06666	21423	tuti	4000	cr44	a_	60	2.6475	3.4675	1.14E-06	-2.47E-06	3.61E-06	3.4275	4.2475	0.82
tuti_06666	21423	tuti	4000	cr44	b_	60	2.6065	3.5905	1.05E-06	-1.66E-06	2.72E-06	3.3865	4.3705	0.984
tuti_06666	21423	tuti	2500	cr44	a_	60	2.8115	3.7135	2.34E-06	-5.24E-06	7.58E-06	3.5915	4.4935	0.902
tuti_06666	21423	tuti	2500	cr44	b_	60	2.8115	3.7135	2.22E-06	-4.28E-06	6.50E-06	3.5915	4.4935	0.902
tuti_06666	21423	tuti	1000	cr44	a_	60	3.0575	4.0825	1.41E-06	-9.31E-07	2.34E-06	3.8375	4.8625	1.025
tuti_06666	21423	tuti	1000	cr44	b_	60	2.8525	4.1235	1.05E-06	-1.36E-06	2.41E-06	3.6325	4.9035	1.271
tuti_06666	21423	tuti	4000	cr54	a_	60	3.0985	3.9185	3.45E-07	-1.83E-06	2.17E-06	3.8785	4.6985	0.82
tuti_06666	21423	tuti	4000	cr54	b_	60	2.9755	3.9185	1.26E-06	-1.84E-06	3.10E-06	3.7555	4.6985	0.943
tuti_06666	21423	tuti	2500	cr54	a_	60	3.0575	4.2055	2.05E-06	-5.20E-06	7.25E-06	3.8375	4.9855	1.148
tuti_06666	21423	tuti	2500	cr54	b_	60	3.1805	4.2465	2.00E-06	-4.98E-06	6.98E-06	3.9605	5.0265	1.066
tuti_06666	21423	tuti	1000	cr54	a_	60	3.7955	4.7385	1.83E-06	-2.35E-06	4.18E-06	4.5755	5.5185	0.943
tuti_06666	21423	tuti	1000	cr54	b_	60	3.5905	4.8615	1.48E-06	-1.62E-06	3.10E-06	4.3705	5.6415	1.271
tuti_06666	21423	tuti	2000	cr54	a_	60	3.2215	4.3695	2.10E-06	-3.20E-06	5.30E-06	4.0015	5.1495	1.148
tuti_06666	21423	tuti	2000	cr54	b_	60	3.3445	4.2465	1.83E-06	-3.10E-06	4.93E-06	4.1245	5.0265	0.902
tuti_06666	21423	tuti	3150	cr54	a_	60	2.9755	4.1235	1.40E-06	-4.89E-06	6.29E-06	3.7555	4.9035	1.148
tuti_06666	21423	tuti	3150	cr54	b_	60	3.0985	4.0005	1.24E-06	-5.42E-06	6.66E-06	3.8785	4.7805	0.902
tuti_06666	21423	tuti	4000	cr64	a_	60	3.1395	3.8365	9.30E-07	-8.26E-07	1.76E-06	3.9195	4.6165	0.697
tuti_06666	21423	tuti	4000	cr64	b_	60	3.0165	3.9595	1.13E-06	-8.75E-07	2.00E-06	3.7965	4.7395	0.943
tuti_06666	21423	tuti	2500	cr64	a_	60	3.2625	4.0415	9.57E-07	-2.16E-06	3.12E-06	4.0425	4.8215	0.779
tuti_06666	21423	tuti	2500	cr64	b_	60	3.1395	4.0825	1.03E-06	-1.94E-06	2.97E-06	3.9195	4.8625	0.943
tuti_06666	21423	tuti	1000	cr64	a_	60	3.5495	4.5745	7.59E-07	-6.40E-07	1.40E-06	4.3295	5.3545	1.025
tuti_06666	21423	tuti	1000	cr64	b_	60	3.6315	4.6565	6.34E-07	-8.26E-07	1.46E-06	4.4115	5.4365	1.025
tuti_06666	21423	tuti	2000	cr64	a_	60	3.2215	3.8365	9.15E-07	-5.55E-07	1.47E-06	4.0015	4.6165	0.615
tuti_06666	21423	tuti	2000	cr64	b_	60	3.3035	4.0005	6.92E-07	-7.04E-07	1.40E-06	4.0835	4.7805	0.697
tuti_06666	21423	tuti	3150	cr64	a_	60	3.0575	4.0825	-4.63E-08	-2.87E-06	2.82E-06	3.8375	4.8625	1.025
tuti_06666	21423	tuti	3150	cr64	b_	60	2.9345	4.1235	6.09E-07	-2.20E-06	2.81E-06	3.7145	4.9035	1.189
tuti_06667	21423	tuti	4000	cr00	a_	60	3.0165	3.8365	1.73E-06	-1.88E-06	3.61E-06	3.7965	4.6165	0.82
tuti_06667	21423	tuti	4000	cr00	b_	60	2.8935	3.8775	1.88E-06	-1.87E-06	3.76E-06	3.6735	4.6575	0.984
tuti_06667	21423	tuti	2500	cr00	a_	60	3.0985	4.0825	3.22E-06	-4.68E-06	7.91E-06	3.8785	4.8625	0.984
tuti_06667	21423	tuti	2500	cr00	b_	60	3.0985	4.1235	3.04E-06	-4.56E-06	7.61E-06	3.8785	4.9035	1.025
tuti_06667	21423	tuti	1000	cr00	a_	60	3.0985	4.4105	2.07E-06	-3.50E-06	5.58E-06	3.8785	5.1905	1.312
tuti_06667	21423	tuti	1000	cr00	b_	60	3.0165	4.2055	1.84E-06	-2.95E-06	4.78E-06	3.7965	4.9855	1.189
tuti_06667	21423	tuti	2000	cr00	a_	60	3.0985	4.2055	3.06E-06	-5.37E-06	8.43E-06	3.8785	4.9855	1.107
tuti_06667	21423	tuti	2000	cr00	b_	60	3.1395	4.2055	3.13E-06	-5.47E-06	8.60E-06	3.9195	4.9855	1.066
tuti_06667	21423	tuti	3150	cr00	a_	60	2.9755	4.0825	2.67E-06	-3.96E-06	6.63E-06	3.7555	4.8625	1.107
tuti_06667	21423	tuti	3150	cr00	b_	60	3.3445	4.1645	2.61E-06	-2.94E-06	5.55E-06	4.1245	4.9445	0.82
tuti_06667	21423	tuti	4000	cr44	a_	60	2.4835	3.5495	1.47E-06	-1.14E-06	2.60E-06	3.2635	4.3295	1.066
tuti_06667	21423	tuti	4000	cr44	b_	60	3.0165	3.4265	1.43E-06	-1.55E-06	2.99E-06	3.7965	4.2065	0.41
tuti_06667	21423	tuti	2500	cr44	a_	60	2.6885	4.0415	2.73E-06	-1.72E-06	4.45E-06	3.4685	4.8215	1.353
tuti_06667	21423	tuti	2500	cr44	b_	60	2.8935	3.9595	2.36E-06	-1.97E-06	4.34E-06	3.6735	4.7395	1.066
tuti_06667	21423	tuti	1000	cr44	a_	60	2.4835	3.4675	8.66E-07	-1.05E-06	1.91E-06	3.2635	4.2475	0.984
tuti_06667	21423	tuti	1000	cr44	b_	60	2.6065	4.3285	8.76E-07	-1.97E-06	2.85E-06	3.3865	5.1085	1.722
tuti_06667	21423	tuti	2000	cr44	a_	60	2.8525	4.0415	1.70E-06	-2.13E-06	3.83E-06	3.6325	4.8215	1.189
tuti_06667	21423	tuti	2000	cr44	b_	60	2.8525	4.1235	1.85E-06	-2.24E-06	4.10E-06	3.6325	4.9035	1.271
tuti_06667	21423	tuti	3150	cr44	a_	60	2.8935	3.7545	2.10E-06	-2.14E-06	4.23E-06	3.6735	4.5345	0.861
tuti_06667	21423	tuti	3150	cr44	b_	60	2.6475	3.7545	2.44E-06	-2.80E-06	5.24E-06	3.4275	4.5345	1.107
tuti_06667	21423	tuti	4000	cr54	a_	60	2.8115	3.4265	7.94E-07	-1.34E-06	2.13E-06	3.5915	4.2065	0.615
tuti_06667	21423	tuti	4000	cr54	b_	60	2.5655	3.4265	1.11E-06	-1.63E-06	2.74E-06	3.3455	4.2065	0.861
tuti_06667	21423	tuti	2500	cr54	a_	60	2.7705	3.9595	1.90E-06	-1.79E-06	3.70E-06	3.5505	4.7395	1.189
tuti_06667	21423	tuti	2500	cr54	b_	60	2.9345	4.0415	1.77E-06	-1.78E-06	3.56E-06	3.7145	4.8215	1.107
tuti_06667	21423	tuti	1000	cr54	a_	60	3.0985	3.7955	5.45E-07	-1.21E-06	1.76E-06	3.8785	4.5755	0.697
tuti_06667	21423	tuti	1000	cr54	b_	60	2.8935	4.4105	5.54E-07	-1.35E-06	1.91E-06	3.6735	5.1905	1.517
tuti_06667	21423	tuti	2000	cr54	a_	60	2.9345	4.1645	1.01E-06	-1.48E-06	2.50E-06	3.7145	4.9445	1.23
tuti_06667	21423	tuti	2000	cr54	b_	60	2.9755	4.1235	5.83E-07	-1.71E-06	2.29E-06	3.7555	4.9035	1.148
tuti_06667	21423	tuti	3150	cr54	a_	60	2.9345	3.6725	1.55E-06	-1.58E-06	3.13E-06	3.7145	4.4525	0.738
tuti_06667	21423	tuti	3150	cr54	b_	60	2.9345	3.7135	2.01E-06	-1.75E-06	3.76E-06	3.7145	4.4935	0.779
tuti_06667	21423	tuti	4000	cr64	a_	60	3.2215	3.7955	1.07E-06	-5.18E-07	1.58E-06	4.0015	4.5755	0.574

Development • Supplementary information

tuti_06667	21423	tuti	4000	cr64	b_	60	3.2215	3.7955	9.45E-07	-6.61E-07	1.61E-06	4.0015	4.5755	0.574
tuti_06667	21423	tuti	2500	cr64	a_	60	2.9345	4.1645	1.26E-06	-1.22E-06	2.48E-06	3.7145	4.9445	1.23
tuti_06667	21423	tuti	2500	cr64	b_	60	3.0575	4.1645	1.29E-06	-1.43E-06	2.72E-06	3.8375	4.9445	1.107
tuti_06667	21423	tuti	1000	cr64	a_	60	2.9755	4.0005	5.18E-07	-1.06E-06	1.57E-06	3.7555	4.7805	1.025
tuti_06667	21423	tuti	1000	cr64	b_	60	3.2625	4.6565	5.44E-07	-7.70E-07	1.31E-06	4.0425	5.4365	1.394
tuti_06667	21423	tuti	3150	cr64	a_	60	3.0165	3.7955	1.01E-06	-1.54E-06	2.55E-06	3.7965	4.5755	0.779
tuti_06667	21423	tuti	3150	cr64	b_	60	3.2625	4.1235	1.26E-06	-1.59E-06	2.85E-06	4.0425	4.9035	0.861
tuti_06667	21423	tuti	2000	cr64	a_	60	2.8525	4.0825	4.43E-07	-1.86E-06	2.31E-06	3.6325	4.8625	1.23
tuti_06667	21423	tuti	2000	cr64	b_	60	2.8935	3.9185	-1.11E-07	-1.34E-06	1.23E-06	3.6735	4.6985	1.025
tuti_06668	21623	tuti	4000	cr00	a_	60	2.9345	3.5495	1.17E-06	-1.45E-06	2.63E-06	3.7145	4.3295	0.615
tuti_06668	21623	tuti	4000	cr00	b_	60	2.8935	3.6725	9.63E-07	-1.75E-06	2.71E-06	3.6735	4.4525	0.779
tuti_06668	21623	tuti	2500	cr00	a_	60	2.9755	3.7955	1.83E-06	-5.11E-06	6.94E-06	3.7555	4.5755	0.82
tuti_06668	21623	tuti	2500	cr00	b_	60	2.9755	3.7955	2.60E-06	-4.47E-06	7.08E-06	3.7555	4.5755	0.82
tuti_06668	21623	tuti	1000	cr00	a_	60	3.2215	3.8775	1.45E-06	-1.34E-06	2.79E-06	4.0015	4.6575	0.656
tuti_06668	21623	tuti	1000	cr00	b_	60	3.0575	3.9185	1.00E-06	-2.48E-06	3.49E-06	3.8375	4.6985	0.861
tuti_06668	21623	tuti	2000	cr00	a_	60	2.9345	4.0005	1.21E-06	-4.82E-06	6.03E-06	3.7145	4.7805	1.066
tuti_06668	21623	tuti	2000	cr00	b_	60	3.1395	3.9595	1.87E-06	-4.03E-06	5.90E-06	3.9195	4.7395	0.82
tuti_06668	21623	tuti	3150	cr00	a_	60	3.0575	3.8775	1.79E-06	-3.81E-06	5.60E-06	3.8375	4.6575	0.82
tuti_06668	21623	tuti	3150	cr00	b_	60	3.0985	3.9185	2.01E-06	-3.77E-06	5.78E-06	3.8785	4.6985	0.82
tuti_06668	21623	tuti	4000	cr44	a_	60	2.7295	3.1805	2.69E-07	-1.41E-06	1.68E-06	3.5095	3.9605	0.451
tuti_06668	21623	tuti	4000	cr44	b_	60	2.7295	3.2215	1.04E-06	-6.36E-07	1.67E-06	3.5095	4.0015	0.492
tuti_06668	21623	tuti	2500	cr44	a_	60	2.9345	3.4265	4.70E-07	-2.78E-06	3.25E-06	3.7145	4.2065	0.492
tuti_06668	21623	tuti	2500	cr44	b_	60	2.8935	3.5905	1.44E-06	-2.04E-06	3.48E-06	3.6735	4.3705	0.697
tuti_06668	21623	tuti	1000	cr44	a_	60	3.1395	4.0415	3.24E-07	-1.60E-06	1.92E-06	3.9195	4.8215	0.902
tuti_06668	21623	tuti	1000	cr44	b_	60	3.0165	3.5495	4.27E-07	-9.48E-07	1.38E-06	3.7965	4.3295	0.533
tuti_06668	21623	tuti	2000	cr44	a_	60	2.8935	3.3445	5.29E-07	-8.69E-07	1.40E-06	3.6735	4.1245	0.451
tuti_06668	21623	tuti	2000	cr44	b_	60	2.8525	3.3855	-8.75E-08	-1.38E-06	1.30E-06	3.6325	4.1655	0.533
tuti_06668	21623	tuti	3150	cr44	a_	60	2.7705	3.3445	1.74E-06	-1.80E-06	3.53E-06	3.5505	4.1245	0.574
tuti_06668	21623	tuti	3150	cr44	b_	60	2.7295	3.5085	1.08E-06	-2.25E-06	3.33E-06	3.5095	4.2885	0.779
tuti_06668	21623	tuti	4000	cr54	a_	60	2.7295	3.3445	1.07E-06	-7.08E-07	1.78E-06	3.5095	4.1245	0.615
tuti_06668	21623	tuti	4000	cr54	b_	60	2.8935	3.3035	7.61E-07	-5.87E-07	1.35E-06	3.6735	4.0835	0.41
tuti_06668	21623	tuti	2500	cr54	a_	60	3.0985	3.8775	7.46E-07	-2.09E-06	2.83E-06	3.8785	4.6575	0.779
tuti_06668	21623	tuti	2500	cr54	b_	60	3.0575	3.4675	3.09E-07	-2.01E-06	2.32E-06	3.8375	4.2475	0.41
tuti_06668	21623	tuti	2000	cr54	a_	60	2.7705	3.9185	9.40E-07	-1.38E-06	2.32E-06	3.5505	4.6985	1.148
tuti_06668	21623	tuti	2000	cr54	b_	60	3.0165	4.0005	5.41E-07	-1.30E-06	1.84E-06	3.7965	4.7805	0.984
tuti_06668	21623	tuti	3150	cr54	a_	60	2.8935	3.7545	1.08E-06	-2.26E-06	3.35E-06	3.6735	4.5345	0.861
tuti_06668	21623	tuti	3150	cr54	b_	60	2.9345	3.3855	1.33E-06	-1.85E-06	3.19E-06	3.7145	4.1655	0.451
tuti_06668	21623	tuti	1000	cr54	a_	60	2.7705	4.1645	5.41E-07	-1.76E-06	2.31E-06	3.5505	4.9445	1.394
tuti_06668	21623	tuti	1000	cr54	b_	60	3.0985	3.7545	-1.58E-07	-1.02E-06	8.64E-07	3.8785	4.5345	0.656
tuti_06668	21623	tuti	4000	cr64	a_	60	4.0005	3.6315	8.16E-07	-5.66E-07	1.38E-06	4.7805	4.4115	-0.369
tuti_06668	21623	tuti	4000	cr64	b_	60	3.0985	3.7955	5.72E-07	-3.24E-07	8.96E-07	3.8785	4.5755	0.697
tuti_06668	21623	tuti	2500	cr64	a_	60	3.0575	3.7135	1.22E-06	-1.41E-06	2.63E-06	3.8375	4.4935	0.656
tuti_06668	21623	tuti	2500	cr64	b_	60	2.9755	3.6725	7.16E-08	-3.22E-06	3.29E-06	3.7555	4.4525	0.697
tuti_06668	21623	tuti	1000	cr64	a_	60	2.9755	3.2625	-6.37E-07	-1.08E-06	4.39E-07	3.7555	4.0425	0.287
tuti_06668	21623	tuti	1000	cr64	b_	60	3.5905	4.6975	4.60E-07	-7.55E-07	1.22E-06	4.3705	5.4775	1.107
tuti_06668	21623	tuti	2000	cr64	a_	60	2.9345	3.7135	4.57E-07	-6.75E-07	1.13E-06	3.7145	4.4935	0.779
tuti_06668	21623	tuti	2000	cr64	b_	60	3.0985	4.0005	3.15E-07	-1.55E-06	1.86E-06	3.8785	4.7805	0.902
tuti_06668	21623	tuti	3150	cr64	a_	60	2.8935	3.7135	8.07E-07	-1.23E-06	2.03E-06	3.6735	4.4935	0.82
tuti_06668	21623	tuti	3150	cr64	b_	60	2.9345	3.7545	9.98E-07	-1.99E-06	2.99E-06	3.7145	4.5345	0.82
tuti_06669	21623	tuti	4000	cr00	a_	60	2.8525	3.5495	7.61E-07	-4.01E-06	4.78E-06	3.6325	4.3295	0.697
tuti_06669	21623	tuti	4000	cr00	b_	60	2.8115	3.5085	2.76E-06	-2.31E-06	5.07E-06	3.5915	4.2885	0.697
tuti_06669	21623	tuti	2500	cr00	a_	60	3.0165	3.7955	4.25E-06	-6.95E-06	1.12E-05	3.7965	4.5755	0.779
tuti_06669	21623	tuti	2500	cr00	b_	60	3.0165	3.7545	3.75E-06	-5.19E-06	8.93E-06	3.7965	4.5345	0.738
tuti_06669	21623	tuti	1000	cr00	a_	60	3.3855	3.7135	1.51E-06	-2.24E-06	3.76E-06	4.1655	4.4935	0.328
tuti_06669	21623	tuti	1000	cr00	b_	60	3.4265	3.7545	-1.74E-07	-3.32E-06	3.15E-06	4.2065	4.5345	0.328
tuti_06669	21623	tuti	2000	cr00	a_	60	3.2625	3.7545	2.05E-06	-4.80E-06	6.86E-06	4.0425	4.5345	0.492
tuti_06669	21623	tuti	2000	cr00	b_	60	3.2625	3.9185	1.82E-06	-4.40E-06	6.22E-06	4.0425	4.6985	0.656
tuti_06669	21623	tuti	3150	cr00	a_	60	2.8935	3.6315	3.19E-06	-6.27E-06	9.45E-06	3.6735	4.4115	0.738
tuti_06669	21623	tuti	3150	cr00	b_	60	2.8935	3.6725	4.12E-06	-6.40E-06	1.05E-05	3.6735	4.4525	0.779

tuti_06669	21623	tuti	4000	cr44	a_	60	2.6065	3.4265	1.90E-06	-1.89E-06	3.80E-06	3.3865	4.2065	0.82
tuti_06669	21623	tuti	4000	cr44	b_	60	3.0165	3.4265	5.54E-07	-2.26E-06	2.81E-06	3.7965	4.2065	0.41
tuti_06669	21623	tuti	2500	cr44	a_	60	2.8935	3.7545	8.84E-07	-3.55E-06	4.44E-06	3.6735	4.5345	0.861
tuti_06669	21623	tuti	2500	cr44	b_	60	3.1395	3.6315	1.78E-06	-3.64E-06	5.43E-06	3.9195	4.4115	0.492
tuti_06669	21623	tuti	1000	cr44	a_	60	3.0985	3.5905	4.22E-07	-1.18E-06	1.60E-06	3.8785	4.3705	0.492
tuti_06669	21623	tuti	1000	cr44	b_	60	3.1805	4.2465	1.22E-07	-2.85E-06	2.97E-06	3.9605	5.0265	1.066
tuti_06669	21623	tuti	2000	cr44	a_	60	3.0575	3.7545	2.05E-06	-1.85E-06	3.90E-06	3.8375	4.5345	0.697
tuti_06669	21623	tuti	2000	cr44	b_	60	3.1805	4.0005	1.40E-06	-1.38E-06	2.79E-06	3.9605	4.7805	0.82
tuti_06669	21623	tuti	3150	cr44	a_	60	2.8115	3.5085	2.28E-06	-2.26E-06	4.54E-06	3.5915	4.2885	0.697
tuti_06669	21623	tuti	3150	cr44	b_	60	3.0165	3.4675	5.96E-07	-3.82E-06	4.41E-06	3.7965	4.2475	0.451
tuti_06669	21623	tuti	4000	cr54	a_	60	2.8935	3.4265	1.91E-06	-2.45E-07	2.16E-06	3.6735	4.2065	0.533
tuti_06669	21623	tuti	4000	cr54	b_	60	3.0575	3.4675	2.00E-06	-6.08E-07	2.61E-06	3.8375	4.2475	0.41
tuti_06669	21623	tuti	2500	cr54	a_	60	3.0165	3.5905	1.50E-06	-1.48E-06	2.98E-06	3.7965	4.3705	0.574
tuti_06669	21623	tuti	2500	cr54	b_	60	2.8115	3.6315	1.11E-06	-1.81E-06	2.92E-06	3.5915	4.4115	0.82
tuti_06669	21623	tuti	1000	cr54	a_	60	3.0985	3.8365	3.20E-07	-7.30E-07	1.05E-06	3.8785	4.6165	0.738
tuti_06669	21623	tuti	1000	cr54	b_	60	3.2215	3.7955	-4.54E-07	-1.75E-06	1.29E-06	4.0015	4.5755	0.574
tuti_06669	21623	tuti	2000	cr54	a_	60	3.1395	4.2055	8.00E-07	-2.02E-06	2.82E-06	3.9195	4.9855	1.066
tuti_06669	21623	tuti	2000	cr54	b_	60	2.9755	3.7135	1.39E-06	-1.17E-06	2.56E-06	3.7555	4.4935	0.738
tuti_06669	21623	tuti	3150	cr54	a_	60	3.0165	3.5085	6.76E-07	-2.06E-06	2.74E-06	3.7965	4.2885	0.492
tuti_06669	21623	tuti	3150	cr54	b_	60	2.9345	3.5495	1.17E-06	-1.77E-06	2.94E-06	3.7145	4.3295	0.615
tuti_06669	21623	tuti	4000	cr64	a_	60	3.3855	3.6725	7.55E-07	4.30E-08	7.12E-07	4.1655	4.4525	0.287
tuti_06669	21623	tuti	4000	cr64	b_	60	3.1395	3.8365	9.54E-07	-2.48E-07	1.20E-06	3.9195	4.6165	0.697
tuti_06669	21623	tuti	2500	cr64	a_	60	3.3035	4.0415	-1.04E-09	-2.43E-06	2.43E-06	4.0835	4.8215	0.738
tuti_06669	21623	tuti	2500	cr64	b_	60	3.0985	4.1645	1.66E-07	-2.11E-06	2.28E-06	3.8785	4.9445	1.066
tuti_06669	21623	tuti	1000	cr64	a_	60	3.4675	3.8365	-6.86E-07	-1.16E-06	4.71E-07	4.2475	4.6165	0.369
tuti_06669	21623	tuti	1000	cr64	b_	60	3.0575	3.8775	7.31E-07	-9.37E-07	1.67E-06	3.8375	4.6575	0.82
tuti_06669	21623	tuti	2000	cr64	a_	60	3.2215	4.1235	6.29E-07	-1.22E-06	1.85E-06	4.0015	4.9035	0.902
tuti_06669	21623	tuti	2000	cr64	b_	60	3.3035	4.0415	1.24E-06	-1.38E-06	2.62E-06	4.0835	4.8215	0.738
tuti_06669	21623	tuti	3150	cr64	a_	60	3.3035	3.8365	8.06E-07	-2.14E-06	2.95E-06	4.0835	4.6165	0.533
tuti_06669	21623	tuti	3150	cr64	b_	60	2.8525	3.8775	7.16E-07	-1.56E-06	2.27E-06	3.6325	4.6575	1.025
tuti_06670	22123	tuti	4000	cr00	a_	60	2.7705	3.5495	1.38E-06	-3.23E-06	4.62E-06	3.5505	4.3295	0.779
tuti_06670	22123	tuti	4000	cr00	b_	60	2.8115	3.5495	1.62E-06	-3.05E-06	4.68E-06	3.5915	4.3295	0.738
tuti_06670	22123	tuti	2500	cr00	a_	60	2.8525	3.8365	2.14E-06	-5.80E-06	7.95E-06	3.6325	4.6165	0.984
tuti_06670	22123	tuti	2500	cr00	b_	60	2.9755	3.8365	2.74E-06	-5.31E-06	8.05E-06	3.7555	4.6165	0.861
tuti_06670	22123	tuti	1000	cr00	a_	60	3.2215	4.1645	4.54E-07	-2.22E-06	2.67E-06	4.0015	4.9445	0.943
tuti_06670	22123	tuti	1000	cr00	b_	60	3.3035	4.1235	7.85E-07	-1.90E-06	2.68E-06	4.0835	4.9035	0.82
tuti_06670	22123	tuti	2000	cr00	a_	60	2.8115	3.7545	2.51E-06	-5.17E-06	7.68E-06	3.5915	4.5345	0.943
tuti_06670	22123	tuti	2000	cr00	b_	60	2.8935	3.7955	2.18E-06	-5.36E-06	7.54E-06	3.6735	4.5755	0.902
tuti_06670	22123	tuti	3150	cr00	a_	60	2.8115	3.6315	3.20E-06	-4.96E-06	8.17E-06	3.5915	4.4115	0.82
tuti_06670	22123	tuti	3150	cr00	b_	60	2.8115	3.5905	1.89E-06	-5.56E-06	7.45E-06	3.5915	4.3705	0.779
tuti_06670	22123	tuti	4000	cr44	a_	60	2.8115	3.6725	1.23E-06	-1.73E-06	2.96E-06	3.5915	4.4525	0.861
tuti_06670	22123	tuti	4000	cr44	b_	60	2.8525	3.7135	1.36E-06	-1.99E-06	3.35E-06	3.6325	4.4935	0.861
tuti_06670	22123	tuti	2500	cr44	a_	60	3.2215	4.1235	1.48E-06	-6.00E-06	7.48E-06	4.0015	4.9035	0.902
tuti_06670	22123	tuti	2500	cr44	b_	60	3.0985	4.1235	1.89E-06	-5.78E-06	7.67E-06	3.8785	4.9035	1.025
tuti_06670	22123	tuti	1000	cr44	a_	60	3.6315	4.6565	1.52E-06	-2.57E-06	4.09E-06	4.4115	5.4365	1.025
tuti_06670	22123	tuti	1000	cr44	b_	60	3.7545	4.6565	8.52E-07	-2.24E-06	3.09E-06	4.5345	5.4365	0.902
tuti_06670	22123	tuti	2000	cr44	a_	60	3.3445	4.2465	1.67E-06	-4.14E-06	5.81E-06	4.1245	5.0265	0.902
tuti_06670	22123	tuti	2000	cr44	b_	60	3.0985	4.2465	1.50E-06	-4.22E-06	5.73E-06	3.8785	5.0265	1.148
tuti_06670	22123	tuti	3150	cr44	a_	60	2.9755	3.8775	2.04E-06	-3.99E-06	6.03E-06	3.7555	4.6575	0.902
tuti_06670	22123	tuti	3150	cr44	b_	60	2.9755	3.8365	1.85E-06	-3.93E-06	5.78E-06	3.7555	4.6165	0.861
tuti_06670	22123	tuti	4000	cr54	a_	60	3.0165	3.7955	1.15E-06	-1.12E-06	2.26E-06	3.7965	4.5755	0.779
tuti_06670	22123	tuti	4000	cr54	b_	60	3.0985	3.7955	1.33E-06	-1.10E-06	2.43E-06	3.8785	4.5755	0.697
tuti_06670	22123	tuti	2500	cr54	a_	60	3.1395	4.2875	1.52E-06	-4.39E-06	5.91E-06	3.9195	5.0675	1.148
tuti_06670	22123	tuti	2500	cr54	b_	60	3.3445	4.3285	1.74E-06	-4.33E-06	6.07E-06	4.1245	5.1085	0.984
tuti_06670	22123	tuti	1000	cr54	a_	60	3.6315	4.7795	1.11E-06	-2.31E-06	3.42E-06	4.4115	5.5595	1.148
tuti_06670	22123	tuti	1000	cr54	b_	60	3.7135	4.8615	6.33E-07	-2.29E-06	2.92E-06	4.4935	5.6415	1.148
tuti_06670	22123	tuti	2000	cr54	a_	60	3.3035	4.3285	1.68E-06	-3.07E-06	4.74E-06	4.0835	5.1085	1.025
tuti_06670	22123	tuti	2000	cr54	b_	60	3.4675	4.4105	1.24E-06	-3.09E-06	4.33E-06	4.2475	5.1905	0.943
tuti_06670	22123	tuti	3150	cr54	a_	60	3.1395	3.9185	1.55E-06	-2.68E-06	4.22E-06	3.9195	4.6985	0.779

tuti_06670	22123	tuti	3150	cr54	b_	60	3.1805	3.9185	1.33E-06	-2.99E-06	4.32E-06	3.9605	4.6985	0.738
tuti_06670	22123	tuti	4000	cr64	a_	60	2.9345	3.5905	3.46E-07	-1.56E-06	1.91E-06	3.7145	4.3705	0.656
tuti_06670	22123	tuti	4000	cr64	b_	60	3.0165	3.5085	4.79E-07	-1.02E-06	1.50E-06	3.7965	4.2885	0.492
tuti_06670	22123	tuti	2500	cr64	a_	60	3.1395	4.0415	1.24E-06	-1.66E-06	2.90E-06	3.9195	4.8215	0.902
tuti_06670	22123	tuti	2500	cr64	b_	60	3.0575	4.1645	1.02E-06	-2.18E-06	3.20E-06	3.8375	4.9445	1.107
tuti_06670	22123	tuti	1000	cr64	a_	60	3.3855	4.6565	6.37E-07	-1.25E-06	1.89E-06	4.1655	5.4365	1.271
tuti_06670	22123	tuti	2000	cr64	a_	60	3.3035	4.0005	8.90E-07	-9.92E-07	1.88E-06	4.0835	4.7805	0.697
tuti_06670	22123	tuti	2000	cr64	b_	60	3.2625	4.0825	4.77E-07	-9.58E-07	1.44E-06	4.0425	4.8625	0.82
tuti_06670	22123	tuti	3150	cr64	a_	60	3.0165	3.9595	1.00E-06	-9.16E-07	1.92E-06	3.7965	4.7395	0.943
tuti_06670	22123	tuti	3150	cr64	b_	60	3.0165	3.7135	1.02E-06	-1.32E-06	2.33E-06	3.7965	4.4935	0.697
wbnu_06671	30223	wbnu	4000	cr00	a_	60	2.9345	3.5905	1.87E-06	-6.78E-07	2.55E-06	3.7145	4.3705	0.656
wbnu_06671	30223	wbnu	4000	cr00	b_	60	3.1805	3.6725	1.52E-06	-8.90E-07	2.41E-06	3.9605	4.4525	0.492
wbnu_06671	30223	wbnu	2500	cr00	a_	60	3.0165	4.0005	4.30E-06	-4.04E-06	8.34E-06	3.7965	4.7805	0.984
wbnu_06671	30223	wbnu	2500	cr00	b_	60	3.0575	4.0005	4.15E-06	-4.35E-06	8.50E-06	3.8375	4.7805	0.943
wbnu_06671	30223	wbnu	1000	cr00	a_	60	3.0985	4.0415	2.69E-06	-1.44E-06	4.13E-06	3.8785	4.8215	0.943
wbnu_06671	30223	wbnu	1000	cr00	b_	60	3.3035	4.3695	2.95E-06	-1.48E-06	4.43E-06	4.0835	5.1495	1.066
wbnu_06671	30223	wbnu	2000	cr00	a_	60	3.1395	4.0415	4.68E-06	-3.87E-06	8.55E-06	3.9195	4.8215	0.902
wbnu_06671	30223	wbnu	2000	cr00	b_	60	3.0575	3.9595	4.66E-06	-3.76E-06	8.42E-06	3.8375	4.7395	0.902
wbnu_06671	30223	wbnu	3150	cr00	a_	60	3.0165	3.9185	3.41E-06	-2.49E-06	5.90E-06	3.7965	4.6985	0.902
wbnu_06671	30223	wbnu	3150	cr00	b_	60	3.0165	3.9595	3.25E-06	-2.45E-06	5.69E-06	3.7965	4.7395	0.943
wbnu_06671	30223	wbnu	4000	cr44	a_	60	3.0165	3.5495	1.32E-06	-6.51E-09	1.33E-06	3.7965	4.3295	0.533
wbnu_06671	30223	wbnu	4000	cr44	b_	60	2.6475	3.5905	9.34E-07	-4.72E-07	1.41E-06	3.4275	4.3705	0.943
wbnu_06671	30223	wbnu	2500	cr44	a_	60	2.9755	3.7955	2.24E-06	-1.62E-06	3.86E-06	3.7555	4.5755	0.82
wbnu_06671	30223	wbnu	2500	cr44	b_	60	3.0575	3.9185	1.80E-06	-1.84E-06	3.64E-06	3.8375	4.6985	0.861
wbnu_06671	30223	wbnu	1000	cr44	a_	60	3.0575	4.4515	1.54E-06	-1.24E-06	2.77E-06	3.8375	5.2315	1.394
wbnu_06671	30223	wbnu	1000	cr44	b_	60	3.3035	4.2055	1.63E-06	-8.41E-07	2.47E-06	4.0835	4.9855	0.902
wbnu_06671	30223	wbnu	2000	cr44	a_	60	3.1805	4.1235	2.19E-06	-1.29E-06	3.49E-06	3.9605	4.9035	0.943
wbnu_06671	30223	wbnu	2000	cr44	b_	60	3.0985	4.2875	2.34E-06	-9.14E-07	3.26E-06	3.8785	5.0675	1.189
wbnu_06671	30223	wbnu	3150	cr44	a_	60	2.8935	3.9185	1.98E-06	-7.56E-07	2.74E-06	3.6735	4.6985	1.025
wbnu_06671	30223	wbnu	3150	cr44	b_	60	3.0985	3.7955	1.59E-06	-1.31E-06	2.91E-06	3.8785	4.5755	0.697
wbnu_06671	30223	wbnu	4000	cr54	b_	60	3.0165	3.8365	-9.53E-07	-2.13E-06	1.17E-06	3.7965	4.6165	0.82
wbnu_06671	30223	wbnu	2500	cr54	a_	60	3.1395	4.0005	1.79E-06	-1.55E-06	3.34E-06	3.9195	4.7805	0.861
wbnu_06671	30223	wbnu	2500	cr54	b_	60	1.7045	4.0415	2.76E-06	-5.24E-07	3.28E-06	2.4845	4.8215	2.337
wbnu_06671	30223	wbnu	1000	cr54	b_	60	3.9185	4.3285	1.34E-06	-8.96E-07	2.23E-06	4.6985	5.1085	0.41
wbnu_06671	30223	wbnu	2000	cr54	a_	60	3.0985	4.0415	1.01E-06	-1.36E-06	2.37E-06	3.8785	4.8215	0.943
wbnu_06671	30223	wbnu	2000	cr54	b_	60	3.4675	4.8205	9.49E-07	-1.81E-06	2.76E-06	4.2475	5.6005	1.353
wbnu_06671	30223	wbnu	3150	cr54	a_	60	2.7705	4.0415	1.95E-06	-2.77E-06	4.73E-06	3.5505	4.8215	1.271
wbnu_06671	30223	wbnu	3150	cr54	b_	60	3.4675	4.6155	1.92E-06	-4.85E-07	2.40E-06	4.2475	5.3955	1.148
wbnu_06671	30223	wbnu	4000	cr64	a_	60	3.2215	3.8365	8.18E-07	-2.28E-07	1.05E-06	4.0015	4.6165	0.615
wbnu_06671	30223	wbnu	4000	cr64	b_	60	2.9755	3.8775	6.34E-07	-3.36E-07	9.70E-07	3.7555	4.6575	0.902
wbnu_06671	30223	wbnu	2500	cr64	a_	60	3.0985	4.2465	7.61E-07	-5.39E-07	1.30E-06	3.8785	5.0265	1.148
wbnu_06671	30223	wbnu	2500	cr64	b_	60	3.1395	4.0825	4.92E-07	-7.72E-07	1.26E-06	3.9195	4.8625	0.943
wbnu_06671	30223	wbnu	1000	cr64	a_	60	3.5905	4.6565	9.30E-07	-2.35E-07	1.16E-06	4.3705	5.4365	1.066
wbnu_06671	30223	wbnu	1000	cr64	b_	60	3.8775	4.3285	6.31E-07	-4.19E-07	1.05E-06	4.6575	5.1085	0.451
wbnu_06671	30223	wbnu	2000	cr64	a_	60	3.7135	3.9595	9.02E-07	-7.29E-07	1.63E-06	4.4935	4.7395	0.246
wbnu_06671	30223	wbnu	2000	cr64	b_	60	2.6885	3.9595	6.86E-07	-1.58E-06	2.27E-06	3.4685	4.7395	1.271
wbnu_06671	30223	wbnu	3150	cr64	a_	60	3.3035	4.0825	7.67E-07	-7.22E-07	1.49E-06	4.0835	4.8625	0.779
wbnu_06671	30223	wbnu	3150	cr64	b_	60	3.5495	4.0825	1.43E-06	-7.00E-07	2.13E-06	4.3295	4.8625	0.533
wbnu_06672	32123	wbnu	2500	cr00	a_	60	3.0985	4.5335	4.43E-06	-4.28E-06	8.70E-06	3.8785	5.3135	1.435
wbnu_06672	32123	wbnu	2500	cr00	b_	60	3.0985	4.4925	4.15E-06	-4.00E-06	8.15E-06	3.8785	5.2725	1.394
wbnu_06672	32123	wbnu	1000	cr00	a_	60	2.6475	4.5745	3.34E-06	-2.62E-06	5.96E-06	3.4275	5.3545	1.927
wbnu_06672	32123	wbnu	1000	cr00	b_	60	2.8935	4.6155	3.35E-06	-2.89E-06	6.24E-06	3.6735	5.3955	1.722
wbnu_06672	32123	wbnu	2000	cr00	a_	60	2.9755	4.5745	4.58E-06	-3.93E-06	8.51E-06	3.7555	5.3545	1.599
wbnu_06672	32123	wbnu	2000	cr00	b_	60	3.0985	4.7385	4.87E-06	-3.21E-06	8.07E-06	3.8785	5.5185	1.64
wbnu_06672	32123	wbnu	3150	cr00	a_	60	3.0165	4.6975	3.57E-06	-1.79E-06	5.35E-06	3.7965	5.4775	1.681
wbnu_06672	32123	wbnu	3150	cr00	b_	60	3.3445	4.7795	3.13E-06	-1.86E-06	4.99E-06	4.1245	5.5595	1.435
wbnu_06672	32123	wbnu	4000	cr00	a_	60	3.2215	4.5745	1.86E-06	-5.78E-08	1.92E-06	4.0015	5.3545	1.353
wbnu_06672	32123	wbnu	4000	cr00	b_	60	2.9755	4.7795	1.69E-06	2.15E-07	1.47E-06	3.7555	5.5595	1.804
wbnu_06672	32123	wbnu	4000	cr44	a_	60	3.0985	4.6975	1.62E-06	-1.58E-07	1.78E-06	3.8785	5.4775	1.599

wbnu_06672	32123	wbnu	4000	cr44	b_	60	3.8775	4.3285	1.76E-06	2.40E-07	1.52E-06	4.6575	5.1085	0.451
wbnu_06672	32123	wbnu	2500	cr44	a_	60	2.9755	4.3695	1.89E-06	-1.53E-06	3.43E-06	3.7555	5.1495	1.394
wbnu_06672	32123	wbnu	2500	cr44	b_	60	3.1395	4.6155	1.80E-06	-1.69E-06	3.49E-06	3.9195	5.3955	1.476
wbnu_06672	32123	wbnu	1000	cr44	a_	60	3.1805	5.1895	1.27E-06	-3.63E-07	1.63E-06	3.9605	5.9695	2.009
wbnu_06672	32123	wbnu	1000	cr44	b_	60	2.5655	4.8205	1.41E-06	-1.30E-06	2.72E-06	3.3455	5.6005	2.255
wbnu_06672	32123	wbnu	2000	cr44	a_	60	3.0165	4.5745	2.07E-06	-1.19E-06	3.25E-06	3.7965	5.3545	1.558
wbnu_06672	32123	wbnu	2000	cr44	b_	60	3.1395	4.6155	1.97E-06	-7.45E-07	2.71E-06	3.9195	5.3955	1.476
wbnu_06672	32123	wbnu	3150	cr44	a_	60	3.4265	4.6565	1.43E-06	-6.70E-07	2.10E-06	4.2065	5.4365	1.23
wbnu_06672	32123	wbnu	3150	cr44	b_	60	3.2215	4.5745	1.50E-06	-5.53E-07	2.05E-06	4.0015	5.3545	1.353
wbnu_06672	32123	wbnu	2500	cr54	a_	60	3.1805	4.6975	1.15E-06	-1.59E-06	2.74E-06	3.9605	5.4775	1.517
wbnu_06672	32123	wbnu	2500	cr54	b_	60	3.2625	4.7385	1.29E-06	-1.45E-06	2.75E-06	4.0425	5.5185	1.476
wbnu_06672	32123	wbnu	1000	cr54	a_	60	4.0005	5.0665	1.05E-06	-7.77E-07	1.83E-06	4.7805	5.8465	1.066
wbnu_06672	32123	wbnu	1000	cr54	b_	60	3.5905	5.1485	1.05E-06	-7.15E-07	1.76E-06	4.3705	5.9285	1.558
wbnu_06672	32123	wbnu	3150	cr54	a_	60	3.0985	4.5335	1.10E-06	-2.79E-07	1.38E-06	3.8785	5.3135	1.435
wbnu_06672	32123	wbnu	3150	cr54	b_	60	3.0575	4.1235	1.13E-06	-5.73E-07	1.71E-06	3.8375	4.9035	1.066
wbnu_06672	32123	wbnu	4000	cr54	a_	60	3.5085	3.9595	9.13E-07	3.62E-07	5.51E-07	4.2885	4.7395	0.451
wbnu_06672	32123	wbnu	2000	cr54	a_	60	3.0575	4.3285	1.18E-06	-4.25E-07	1.61E-06	3.8375	5.1085	1.271
wbnu_06672	32123	wbnu	2000	cr54	b_	60	3.1395	4.7795	1.14E-06	-3.76E-07	1.52E-06	3.9195	5.5595	1.64
wbnu_06672	32123	wbnu	4000	cr64	a_	60	2.6475	3.3445	8.36E-07	-3.63E-07	1.20E-06	3.4275	4.1245	0.697
wbnu_06672	32123	wbnu	4000	cr64	b_	60	3.2215	3.8775	6.36E-07	1.56E-07	4.80E-07	4.0015	4.6575	0.656
wbnu_06672	32123	wbnu	2500	cr64	a_	60	3.3035	4.3695	5.80E-07	-1.00E-06	1.58E-06	4.0835	5.1495	1.066
wbnu_06672	32123	wbnu	2500	cr64	b_	60	3.1395	4.0415	8.68E-07	-8.67E-07	1.74E-06	3.9195	4.8215	0.902
wbnu_06672	32123	wbnu	1000	cr64	a_	60	3.1395	4.1235	5.07E-07	-2.36E-07	7.43E-07	3.9195	4.9035	0.984
wbnu_06672	32123	wbnu	1000	cr64	b_	60	2.4015	3.3855	5.89E-07	-2.09E-07	7.98E-07	3.1815	4.1655	0.984
wbnu_06672	32123	wbnu	2000	cr64	a_	60	3.4675	4.4105	9.52E-07	-4.56E-07	1.41E-06	4.2475	5.1905	0.943
wbnu_06672	32123	wbnu	2000	cr64	b_	60	3.3445	2.7705	7.03E-07	-6.36E-07	1.34E-06	4.1245	3.5505	-0.574
wbnu_06672	32123	wbnu	3150	cr64	a_	60	1.3355	1.9095	9.61E-07	-5.29E-07	1.49E-06	2.1155	2.6895	0.574
wbnu_06672	32123	wbnu	3150	cr64	b_	60	2.1965	2.7295	3.85E-07	-8.83E-07	1.27E-06	2.9765	3.5095	0.533
wbnu_06673	33023	wbnu	4000	cr00	a_	60	3.1395	4.0415	1.72E-06	-9.56E-08	1.82E-06	3.9195	4.8215	0.902
wbnu_06673	33023	wbnu	4000	cr00	b_	60	3.3855	3.9595	1.28E-06	-2.78E-07	1.56E-06	4.1655	4.7395	0.574
wbnu_06673	33023	wbnu	2500	cr00	a_	60	3.3035	4.4515	2.68E-06	-3.51E-06	6.19E-06	4.0835	5.2315	1.148
wbnu_06673	33023	wbnu	2500	cr00	b_	60	3.3855	4.5745	2.94E-06	-3.25E-06	6.19E-06	4.1655	5.3545	1.189
wbnu_06673	33023	wbnu	2000	cr00	a_	60	3.3855	4.4515	3.71E-06	-3.68E-06	7.38E-06	4.1655	5.2315	1.066
wbnu_06673	33023	wbnu	2000	cr00	b_	60	3.3855	4.4925	3.70E-06	-3.45E-06	7.15E-06	4.1655	5.2725	1.107
wbnu_06673	33023	wbnu	3150	cr00	a_	60	3.0575	4.1645	2.13E-06	-1.48E-06	3.62E-06	3.8375	4.9445	1.107
wbnu_06673	33023	wbnu	3150	cr00	b_	60	3.1395	4.1235	1.84E-06	-1.73E-06	3.57E-06	3.9195	4.9035	0.984
wbnu_06673	33023	wbnu	1000	cr00	a_	60	3.3445	4.7385	2.94E-06	-1.54E-06	4.47E-06	4.1245	5.5185	1.394
wbnu_06673	33023	wbnu	1000	cr00	b_	60	3.5495	4.8615	2.97E-06	-1.34E-06	4.31E-06	4.3295	5.6415	1.312
wbnu_06673	33023	wbnu	4000	cr44	a_	60	2.6885	3.7135	1.00E-06	-3.35E-07	1.34E-06	3.4685	4.4935	1.025
wbnu_06673	33023	wbnu	4000	cr44	b_	60	3.3035	3.6725	7.76E-07	-7.67E-08	8.53E-07	4.0835	4.4525	0.369
wbnu_06673	33023	wbnu	2500	cr44	a_	60	3.0575	4.0005	1.72E-06	-1.15E-06	2.87E-06	3.8375	4.7805	0.943
wbnu_06673	33023	wbnu	2500	cr44	b_	60	3.0985	4.1235	1.50E-06	-1.42E-06	2.92E-06	3.8785	4.9035	1.025
wbnu_06673	33023	wbnu	1000	cr44	a_	60	3.5905	5.1895	1.67E-06	-8.78E-07	2.55E-06	4.3705	5.9695	1.599
wbnu_06673	33023	wbnu	1000	cr44	b_	60	3.5495	4.9435	1.42E-06	-6.91E-07	2.11E-06	4.3295	5.7235	1.394
wbnu_06673	33023	wbnu	2000	cr44	a_	60	3.2215	4.1645	1.95E-06	-1.21E-06	3.16E-06	4.0015	4.9445	0.943
wbnu_06673	33023	wbnu	2000	cr44	b_	60	3.2625	3.9595	2.06E-06	-1.19E-06	3.26E-06	4.0425	4.7395	0.697
wbnu_06673	33023	wbnu	3150	cr44	a_	60	2.9345	3.9595	1.54E-06	-5.70E-07	2.11E-06	3.7145	4.7395	1.025
wbnu_06673	33023	wbnu	3150	cr44	b_	60	3.3035	3.9595	1.36E-06	-6.40E-07	2.00E-06	4.0835	4.7395	0.656
wbnu_06673	33023	wbnu	4000	cr54	a_	60	2.7705	4.0005	7.22E-07	-4.86E-07	1.21E-06	3.5505	4.7805	1.23
wbnu_06673	33023	wbnu	4000	cr54	b_	60	3.0575	4.0005	6.66E-07	-3.12E-07	9.78E-07	3.8375	4.7805	0.943
wbnu_06673	33023	wbnu	2500	cr54	a_	60	3.0165	4.3695	1.00E-06	-1.38E-06	2.38E-06	3.7965	5.1495	1.353
wbnu_06673	33023	wbnu	2500	cr54	b_	60	3.4265	4.1235	1.12E-06	-1.04E-06	2.16E-06	4.2065	4.9035	0.697
wbnu_06673	33023	wbnu	1000	cr54	a_	60	3.5905	4.6565	9.35E-07	-4.73E-07	1.41E-06	4.3705	5.4365	1.066
wbnu_06673	33023	wbnu	1000	cr54	b_	60	3.3445	4.6975	7.83E-07	-3.56E-07	1.14E-06	4.1245	5.4775	1.353
wbnu_06673	33023	wbnu	2000	cr54	a_	60	3.2215	4.2055	6.48E-07	-9.30E-07	1.58E-06	4.0015	4.9855	0.984
wbnu_06673	33023	wbnu	2000	cr54	b_	60	3.4265	4.2055	9.97E-07	-1.01E-06	2.01E-06	4.2065	4.9855	0.779
wbnu_06673	33023	wbnu	3150	cr54	a_	60	3.2215	4.2465	1.12E-06	-8.32E-07	1.95E-06	4.0015	5.0265	1.025
wbnu_06673	33023	wbnu	3150	cr54	b_	60	2.9755	4.4105	7.69E-07	-6.01E-07	1.37E-06	3.7555	5.1905	1.435
wbnu_06673	33023	wbnu	4000	cr64	a_	60	2.8935	4.1235	1.93E-07	-5.42E-07	7.35E-07	3.6735	4.9035	1.23

wbnu_06673	33023	wbnu	4000	cr64	b_	60	3.1805	4.1645	3.07E-07	-3.16E-07	6.23E-07	3.9605	4.9445	0.984
wbnu_06673	33023	wbnu	2500	cr64	a_	60	3.0985	4.4515	7.26E-07	-1.29E-06	2.02E-06	3.8785	5.2315	1.353
wbnu_06673	33023	wbnu	2500	cr64	b_	60	3.2215	4.2055	8.03E-07	-6.19E-07	1.42E-06	4.0015	4.9855	0.984
wbnu_06673	33023	wbnu	1000	cr64	a_	60	3.7955	5.4355	5.89E-07	-3.26E-07	9.14E-07	4.5755	6.2155	1.64
wbnu_06673	33023	wbnu	1000	cr64	b_	60	3.8775	5.1075	5.26E-07	-3.35E-07	8.61E-07	4.6575	5.8875	1.23
wbnu_06673	33023	wbnu	2000	cr64	a_	60	3.5085	4.7795	6.75E-07	-8.07E-07	1.48E-06	4.2885	5.5595	1.271
wbnu_06673	33023	wbnu	2000	cr64	b_	60	3.7545	4.5745	5.29E-07	-5.58E-07	1.09E-06	4.5345	5.3545	0.82
wbnu_06673	33023	wbnu	3150	cr64	a_	60	3.0985	4.4515	7.14E-07	-3.60E-07	1.07E-06	3.8785	5.2315	1.353
wbnu_06673	33023	wbnu	3150	cr64	b_	60	2.9755	4.5745	6.22E-07	-5.65E-07	1.19E-06	3.7555	5.3545	1.599
wbnu_06663	32323	wbnu	4000	cr00	a_	60	2.8935	4.0005	1.96E-06	-1.07E-06	3.03E-06	3.6735	4.7805	1.107
wbnu_06663	32323	wbnu	4000	cr00	b_	60	3.0985	3.9185	1.60E-06	-1.26E-06	2.86E-06	3.8785	4.6985	0.82
wbnu_06663	32323	wbnu	2500	cr00	a_	60	2.9755	4.2055	4.30E-06	-4.98E-06	9.27E-06	3.7555	4.9855	1.23
wbnu_06663	32323	wbnu	2500	cr00	b_	60	2.9755	4.2055	3.87E-06	-4.60E-06	8.47E-06	3.7555	4.9855	1.23
wbnu_06663	32323	wbnu	1000	cr00	a_	60	3.2625	4.3695	3.09E-06	-1.99E-06	5.08E-06	4.0425	5.1495	1.107
wbnu_06663	32323	wbnu	1000	cr00	b_	60	3.3855	4.4105	3.51E-06	-1.78E-06	5.30E-06	4.1655	5.1905	1.025
wbnu_06663	32323	wbnu	2000	cr00	a_	60	2.9345	4.0825	5.15E-06	-4.62E-06	9.78E-06	3.7145	4.8625	1.148
wbnu_06663	32323	wbnu	2000	cr00	b_	60	2.9345	4.0825	4.92E-06	-4.02E-06	8.94E-06	3.7145	4.8625	1.148
wbnu_06663	32323	wbnu	3150	cr00	a_	60	2.8115	4.0005	3.31E-06	-2.79E-06	6.10E-06	3.5915	4.7805	1.189
wbnu_06663	32323	wbnu	3150	cr00	b_	60	2.8525	4.1235	2.94E-06	-2.33E-06	5.27E-06	3.6325	4.9035	1.271
wbnu_06663	32323	wbnu	4000	cr44	a_	60	2.8935	3.7135	8.73E-07	-1.77E-07	1.05E-06	3.6735	4.4935	0.82
wbnu_06663	32323	wbnu	4000	cr44	b_	60	3.0165	3.7135	1.06E-06	7.21E-08	9.83E-07	3.7965	4.4935	0.697
wbnu_06663	32323	wbnu	2500	cr44	a_	60	2.8935	4.2875	1.74E-06	-1.67E-06	3.40E-06	3.6735	5.0675	1.394
wbnu_06663	32323	wbnu	2500	cr44	b_	60	2.9755	4.2055	2.21E-06	-1.18E-06	3.39E-06	3.7555	4.9855	1.23
wbnu_06663	32323	wbnu	1000	cr44	a_	60	3.5085	4.5745	1.36E-06	-1.29E-06	2.65E-06	4.2885	5.3545	1.066
wbnu_06663	32323	wbnu	1000	cr44	b_	60	3.0575	4.5335	1.49E-06	-1.29E-06	2.78E-06	3.8375	5.3135	1.476
wbnu_06663	32323	wbnu	2000	cr44	a_	60	2.9345	4.4105	1.77E-06	-1.38E-06	3.15E-06	3.7145	5.1905	1.476
wbnu_06663	32323	wbnu	2000	cr44	b_	60	3.0165	4.3695	2.38E-06	-1.33E-06	3.71E-06	3.7965	5.1495	1.353
wbnu_06663	32323	wbnu	3150	cr44	a_	60	2.9345	4.1235	1.77E-06	-5.97E-07	2.36E-06	3.7145	4.9035	1.189
wbnu_06663	32323	wbnu	3150	cr44	b_	60	3.0985	4.0825	1.22E-06	-5.67E-07	1.78E-06	3.8785	4.8625	0.984
wbnu_06663	32323	wbnu	4000	cr54	a_	60	3.0165	4.0415	5.31E-07	-4.43E-07	9.74E-07	3.7965	4.8215	1.025
wbnu_06663	32323	wbnu	4000	cr54	b_	60	3.3035	4.0005	6.11E-07	-5.29E-07	1.14E-06	4.0835	4.7805	0.697
wbnu_06663	32323	wbnu	2500	cr54	a_	60	2.8115	4.2875	1.31E-06	-9.66E-07	2.27E-06	3.5915	5.0675	1.476
wbnu_06663	32323	wbnu	2500	cr54	b_	60	3.0575	4.4515	9.12E-07	-1.63E-06	2.54E-06	3.8375	5.2315	1.394
wbnu_06663	32323	wbnu	1000	cr54	a_	60	3.3445	4.1645	9.39E-07	-8.54E-07	1.79E-06	4.1245	4.9445	0.82
wbnu_06663	32323	wbnu	1000	cr54	b_	60	3.3035	4.6565	1.06E-06	-1.57E-06	2.62E-06	4.0835	5.4365	1.353
wbnu_06663	32323	wbnu	2000	cr54	a_	60	3.0165	4.3285	1.21E-06	-1.28E-06	2.48E-06	3.7965	5.1085	1.312
wbnu_06663	32323	wbnu	2000	cr54	b_	60	3.2215	4.1645	1.61E-06	-6.72E-07	2.29E-06	4.0015	4.9445	0.943
wbnu_06663	32323	wbnu	3150	cr54	a_	60	3.2625	4.0415	1.01E-06	-7.34E-07	1.75E-06	4.0425	4.8215	0.779
wbnu_06663	32323	wbnu	3150	cr54	b_	60	2.8525	4.0005	9.56E-07	-3.22E-07	1.28E-06	3.6325	4.7805	1.148
wbnu_06663	32323	wbnu	4000	cr64	a_	60	3.3445	3.9595	6.60E-07	-8.06E-08	7.41E-07	4.1245	4.7395	0.615
wbnu_06663	32323	wbnu	4000	cr64	b_	60	3.3035	4.2465	5.65E-07	-4.03E-09	5.69E-07	4.0835	5.0265	0.943
wbnu_06663	32323	wbnu	2500	cr64	a_	60	3.5085	4.1645	4.48E-07	-4.73E-07	9.21E-07	4.2885	4.9445	0.656
wbnu_06663	32323	wbnu	2500	cr64	b_	60	3.2625	4.2055	7.16E-07	-6.93E-07	1.41E-06	4.0425	4.9855	0.943
wbnu_06663	32323	wbnu	1000	cr64	a_	60	3.3855	4.5745	6.09E-07	-5.53E-07	1.16E-06	4.1655	5.3545	1.189
wbnu_06663	32323	wbnu	1000	cr64	b_	60	3.7135	4.6155	4.64E-07	-6.03E-07	1.07E-06	4.4935	5.3955	0.902
wbnu_06663	32323	wbnu	2000	cr64	a_	60	3.2625	4.0825	8.07E-07	-5.81E-07	1.39E-06	4.0425	4.8625	0.82
wbnu_06663	32323	wbnu	2000	cr64	b_	60	3.4675	3.9185	2.11E-07	-7.02E-07	9.13E-07	4.2475	4.6985	0.451
wbnu_06663	32323	wbnu	3150	cr64	a_	60	3.6315	4.2055	6.26E-07	-5.54E-07	1.18E-06	4.4115	4.9855	0.574
wbnu_06663	32323	wbnu	3150	cr64	b_	60	3.0575	3.4265	4.37E-07	-3.02E-07	7.38E-07	3.8375	4.2065	0.369

Development • Supplementary information

**ID**	Bird ID Number
**Date**	Date
**Species**	Species (bcch, tuti, wbnu)
**Frequency**	Tone Frequency (1, 2, 2.5, 3.15, 4 kHz)
**Noise**	Noise Level (cr00, cr44, cr54, cr64)
**Rep**	Repetition (e.g. a or b given that there were two repetitions at each level)
**Intensity**	Sound Level of the tone (60 dB, showing superthreshold responses)
**Latency_Max**	Time to Positive Peak since onset of sound at the ear (ms)
**Latency_Min**	Time to Negative Peak since onset of sound at the ear (ms)
**Max**	Voltage for the Postiive Peak
**Min**	Voltage for the Negative Peak
**Amp**	Amplitude (difference between the Max and the Min)
**Latency_Max2**	Time to the Positive Peak since the beginning of the sound playback (ms)
**Latency_Min2**	Time to the Negative Peak since the beginning of the sound playback (ms)
**InterLatency**	Time between peaks (ms)

Script 1. SAS code used to create statistical models

```
proc mixed data = data;
title "FULL Threshold model with AR";
class ID species noise_level frequency;
model threshold = noise_level | species | frequency
/ ddfm = KR;
repeated frequency*noise_level/ subject = ID type=AR(1);
run;

proc mixed data = data;
title "REDUCED Threshold model with AR";
class ID species noise_level frequency;
model threshold = noise_level  species  frequency
noise_level*species
species*frequency
/ ddfm = KR;
repeated frequency*noise_level/ subject = ID type=AR(1);
lsmeans species  / diffs;
lsmeans noise_level  / diffs;
lsmeans frequency  / diffs;
lsmeans species*frequency  /  diffs;
lsmeans species*noise_level  /  diffs;
run;

data data_log;
set data;
log = log10(critical_ratio);
run;

proc mixed data = data_log;
title "FULL Log critical ratio model with AR";
class ID species noise_level frequency;
model log = noise_level | species | frequency
/ ddfm = KR;
repeated / subject = ID type=AR(1);
run;

proc mixed data = data_log;
title "REDUCED Log critical ratio model with AR";
class ID species noise_level frequency;
model log = species noise_level frequency
species*frequency
/ ddfm = KR;
repeated / subject = ID type=AR(1);
lsmeans species  / diffs;
lsmeans noise_level  / diffs;
lsmeans frequency  / diffs;
lsmeans species*frequency  / diffs;
run;

data LogAmp;
```

```
set AmpLat;
log = log10(amp);
run;

proc mixed data = data_log;
title "FULL Log critical ratio model with AR";
class ID species noise_level frequency;
model log = noise_level | species | frequency
/ ddfm = KR;
repeated / subject = ID type=AR(1);
run;

proc mixed data = LogAmp;
title "Reduced Amp model with AR";
class ID species noise frequency;
model log = species noise frequency
noise*species
species*frequency
noise*frequency
/ ddfm = KR;
repeated / subject = ID type=AR(1);
lsmeans species  / diffs;
lsmeans noise_level  / diffs;
lsmeans frequency  / diffs;
lsmeans noise*species  / diffs;
lsmeans species*frequency  / diffs;
lsmeans noise*frequency  / diffs;
run;

proc mixed data = AmpLat;
title "FULL Latency Max model with AR";
class ID species noise frequency;
model latency_max2 = noise | species | frequency
/ ddfm = KR;
repeated / subject = ID type=AR(1);
run;

proc mixed data = AmpLat;
title "Reduced Latency Max model with AR";
class ID species noise frequency;
model latency_max2 = species noise frequency
noise*frequency
species*frequency
/ ddfm = KR;
repeated / subject = ID type=AR(1);
lsmeans species  / diffs;
lsmeans noise  / diffs;
lsmeans frequency  / diffs;
lsmeans noise*frequency  / diffs;
lsmeans species*frequency  / diffs;
```

Development • Supplementary information

```
run;

proc mixed data = AmpLat;
title "FULL Latency Min model with AR";
class ID noise species frequency;
model latency_min2 = noise | species | frequency
/ ddfm = KR;
repeated / subject = ID type=AR(1);
run;

proc mixed data = AmpLat;
title "Reduced Latency Min model with AR";
class ID species noise frequency;
model latency_min2 = noise species frequency
species*frequency
noise*frequency
/ ddfm = KR;
repeated / subject = ID type=AR(1);
lsmeans species  / diffs;
lsmeans noise_level  / diffs;
lsmeans frequency  / diffs;
lsmeans noise*frequency  / diffs;
lsmeans species*frequency  / diffs;
run;
```

Development • Supplementary information
